# Optimizing the maximum reported cluster size for the multinomial-based spatial scan statistic

**DOI:** 10.1186/s12942-023-00353-4

**Published:** 2023-11-08

**Authors:** Jisu Moon, Minseok Kim, Inkyung Jung

**Affiliations:** Division of Biostatistics, Department of Biomedical Systems Informatics, Yonsei University College of Medicine, 50-1 Yonsei-ro, Seodaemun-gu, Seoul, 03722 Korea

**Keywords:** Information criterion, Gini coefficient, Maximum scanning window size, SaTScan, Spatial cluster detection

## Abstract

**Background:**

Correctly identifying spatial disease cluster is a fundamental concern in public health and epidemiology. The spatial scan statistic is widely used for detecting spatial disease clusters in spatial epidemiology and disease surveillance. Many studies default to a maximum reported cluster size (MRCS) set at 50% of the total population when searching for spatial clusters. However, this default setting can sometimes report clusters larger than true clusters, which include less relevant regions. For the Poisson, Bernoulli, ordinal, normal, and exponential models, a Gini coefficient has been developed to optimize the MRCS. Yet, no measure is available for the multinomial model.

**Results:**

We propose two versions of a spatial cluster information criterion (SCIC) for selecting the optimal MRCS value for the multinomial-based spatial scan statistic. Our simulation study suggests that SCIC improves the accuracy of reporting true clusters. Analysis of the Korea Community Health Survey (KCHS) data further demonstrates that our method identifies more meaningful small clusters compared to the default setting.

**Conclusions:**

Our method focuses on improving the performance of the spatial scan statistic by optimizing the MRCS value when using the multinomial model. In public health and disease surveillance, the proposed method can be used to provide more accurate and meaningful spatial cluster detection for multinomial data, such as disease subtypes.

**Supplementary Information:**

The online version contains supplementary material available at 10.1186/s12942-023-00353-4.

## Introduction

In public health and disease surveillance, the spatial scan statistic is a widely used method for identifying spatial clusters with significantly high or low risk of disease outcomes. This method is based on the likelihood ratio test statistic for each scanning window to compare its inside and outside. The scanning window that maximizes the test statistic is identified as the most likely cluster. Secondary clusters with high values of the test statistics are also identified. The statistical significance of the most likely cluster and secondary clusters is determined using the Monte Carlo hypothesis testing. The spatial scan statistic has been developed for various probability models such as Poisson [1], Bernoulli [1], exponential [2], ordinal [3], normal [4, 5], and multinomial [6]. SaTScan™ software is freely available for conducting spatial cluster detection analysis using various models of the spatial scan statistic.

The spatial scan statistic differs from spatial clustering methods such as ADCN [7] and STICC [8] in that the method is designed for identifying clusters rather than dividing spatial data into distinct subgroups. A cluster is defined as geographically and/or temporally bounded group of occurrences of sufficient size and concentration to be unlikely to have occurred by chance [9]. The clusters are characterized by the statistical distribution of outcome, not just by distance between geographic objects as in density-based clustering. Spatial clustering methods are commonly used in geodata mining [10–12], while the spatial scan statistic is widely utilized for detecting geographic disease clusters [13–15].

In SaTScan™, researchers are required to specify the scanning window shape and the maximum scanning window size (MSWS). In many studies, the MSWS value is set to the default setting, which is 50% of the total population. A simulation study by Ribeiro and Costa [16] revealed that spatial cluster detection results can vary depending on the MSWS value. Nevertheless, their findings do not suggest running the analysis multiple times with different MSWS values to find the best results, as it may lead to a multiple testing problem, as argued by Han et al. [17]. They proposed an alternative approach, suggesting that the analysis should be rerun with a fixed large MSWS value while adjusting the maximum reported cluster size (MRCS) values. Setting the MRCS value to the default 50% may result in the reporting of clusters larger than the true clusters, encompassing less meaningful regions. Therefore, it is advisable to carefully select an optimal MRCS value.

Several studies have recently developed criteria to select the optimal value of the MRCS. Han et al. [17] proposed an optimization criterion using the Gini coefficient [18] specifically for the Poisson-based spatial scan statistic. Their simulation study showed that the proposed Gini coefficient effectively identified the correct clusters. However, it is important to note that the Gini coefficient needs to be defined differently for different probability models. Kim and Jung [19], Yoo and Jung [20], and Lee et al. [21] developed the Gini coefficient for the ordinal-, normal-, and exponential-based spatial scan statistics, respectively. Yet, no Gini coefficient has been developed for the multinomial-based spatial scan statistic. The difficulty in defining a clear Gini coefficient for the multinomial-based spatial scan statistic arises from its inapplicability to nominal values.

Other studies [22–24] have proposed alternative criteria for selecting the optimal MRCS or MSWS. However, these studies only evaluated the performance of their methods for the Poisson-based spatial scan statistic. Because the methods are likelihood-based optimization criteria, they can potentially be extended to other probability models. Nevertheless, it remains crucial to carefully evaluate the effectiveness of these methods when applied to probability models other than the Poisson model.

In this study, we propose a spatial cluster information criterion (SCIC) inspired by the formulation of the Bayes Information Criterion (BIC) [25] to choose the optimal MRCS value for the multinomial-based spatial scan statistic. The SCIC can be defined for the spatial scan statistic irrespective of the underlying probability model, as its approach is rooted in the likelihood ratio test statistic. To assess the performance of our proposed method, we conducted a simulation study for both the multinomial-based and ordinal-based spatial scan statistics. We compared the performance of our proposed method with that of existing approaches. To exemplify the methodology, we utilized the Korea Community Health Survey (KCHS) data collected by the Korea Centers for Disease Control and Prevention.

## Methods

### Spatial scan statistic for multinomial data

The multinomial-based spatial scan statistic [6] is used to detect disease clusters with statistically different disease-type distributions. Let ${p}_{k}$ and ${q}_{k}$ denote the probabilities of category $k$ inside and outside the scanning window $z$, respectively. If we want to identify regions with different disease-type distributions, the null and alternative hypotheses are stated as

$${{H}_{0}}: {{p}_{1}}={{q}_{1}}, \ldots, {{p}_{K}}={{q}_{K}}\; for\;all\;z\in Z\quad v.s. \quad {{H}_{1}}: not \, {{H}_{0}}$$

where $Z$ denotes the set of all scanning windows and $K$ denotes the total number of categories. The likelihood ratio test statistic, given the scanning window *z*, is denoted as

$${\lambda }_{z}=\frac{\prod _{k}\left\{{\left(\frac{\sum _{i\in z}{c}_{ik}}{\sum _{k}\sum _{i\in z}{c}_{ik}}\right)}^{\sum _{i\in z}{c}_{ik}}\cdot {\left(\frac{\sum _{i\notin z}{c}_{ik}}{\sum _{k}\sum _{i\notin z}{c}_{ik}}\right)}^{\sum _{i\notin z}{c}_{ik}}\right\}}{\prod _{k}\left\{{\left(\frac{{C}_{k}}{C}\right)}^{{C}_{k}}\right\}}$$

where ${c}_{ik}$ is the number of cases belonging to category $k$ inside the region $i$, ${C}_{k}$ is the total number of cases belonging to category $k$ in the whole study area and $C$ is the total number of cases in the whole study area.

### Spatial cluster information criterion (SCIC)

Now we propose an optimization criterion called the spatial cluster information criterion (SCIC) for selecting the optimal MRCS value. Our criterion draws inspiration from the formulation of the Bayes information criterion (BIC) [25], which is a widely used criterion in statistical modeling for model selection. The BIC for a candidate model ${M}_{u}$ is defined as

$$BIC\left({M}_{u}\right)=-2\cdot logL\left(\widehat{{\theta }_{u}}|y\right)+u\cdot log\left(v\right),$$

where $y$ is observed data, $L\left({\theta }_{u}|y\right)$ is the likelihood of $y$ given the model ${M}_{u}$, $\widehat{{\theta }_{u}}$ is the maximum likelihood estimation (MLE) of ${\theta }_{u}$ that maximizes the $L\left({\theta }_{u}|y\right)$, $u$ is the number of parameters in the model ${M}_{u}$, and $v$ is the total number of observations. The BIC equation includes a penalty term as the second component, which penalizes models with additional parameters. The model exhibiting the minimum BIC value is considered the most appropriate selection [26].

We define the SCIC as the sum of the LLR test statistic for all significant clusters, along with a penalty term. In the multinomial-based spatial scan statistic, the LLR test statistic for each scanning window is used to measure the degree of heterogeneity in the spatial distribution of the categories. A higher LLR test statistic indicates a greater degree of heterogeneity within the scanning window compared to the surrounding area. However, as the scanning window size increases, there is a tendency for the LLR test statistic to rise due to the growing number of cases included within the window.

The spatial scan statistic has faced criticism for its tendency to identify clusters that are considerably larger than the actual clusters, often incorporating neighboring regions with no elevated risk of disease occurrence [27–29]. This tendency is mainly noticeable when the default settings of MSWS and MRCS, both set at 50%, are used with circular scanning windows. Optimizing the MRCS improves the spatial scan statistic’s ability to identify clusters with greater precision [17, 19–21]. To utilize the sum of the LRT statistics as an optimizing criterion, we need to offset the inflation of the test statistic due to a large number of observations within the window.

The penalty term in the SCIC is defined in two versions. In the first version, the penalty term is calculated by multiplying the logarithm of the number of cases within the significant clusters by the product of the number of categories and the number of significant clusters. In the second version, we substitute the number of regions inside the significant clusters for the number of cases. This is based on the understanding that the number of cases within a cluster tends to increase as the number of regions inside the cluster increases. Both versions serve as optimization criteria with similar implications. For the multinomial model, the algorithm for computing the SCIC is as follows:

- (Step 1) For a given MRCS $m$% ($m$=1, …, 50), denote ${J}_{m}$ significant clusters reported using the multinomial-based spatial scan statistic by ${Z}_{1}^{\left(m\right)}, \cdots , {Z}_{{J}_{m}}^{\left(m\right)}$.
- (Step 2) For each $m$, calculate the SCIC for all significant clusters as follows:
  Version 1 $${SCIC}_{1}\left(m\right)=-2\sum _{j=1}^{{J}_{m}}log\left({\lambda }_{{Z}_{j}^{\left(m\right)}}\right)+K\cdot {J}_{m}\cdot log\left({\tau }^{\left(m\right)}\right)$$
  Version 2 $${SCIC}_{2}\left(m\right)=-2\sum _{j=1}^{{J}_{m}}log\left({\lambda }_{{Z}_{j}^{\left(m\right)}}\right)+K\cdot {J}_{m}\cdot log\left({\delta }^{\left(m\right)}\right)$$
  where ${\lambda }_{{Z}_{j}^{\left(m\right)}}$ denotes the LRT statistic for the multinomial-based spatial statistic given the ${j}^{th}$ significant cluster ${Z}_{j}^{\left(m\right)}$, $K$ is the total number of categories, and ${\tau }^{\left(m\right)}$ and ${\delta }^{\left(m\right)}$ denote the sum of the number of total cases and the sum of the number of regions inside all significant clusters, respectively.
- (Step 3) Choose the MRCS which minimizes the SCIC as the optimal MRCS.

**Fig. 1:**
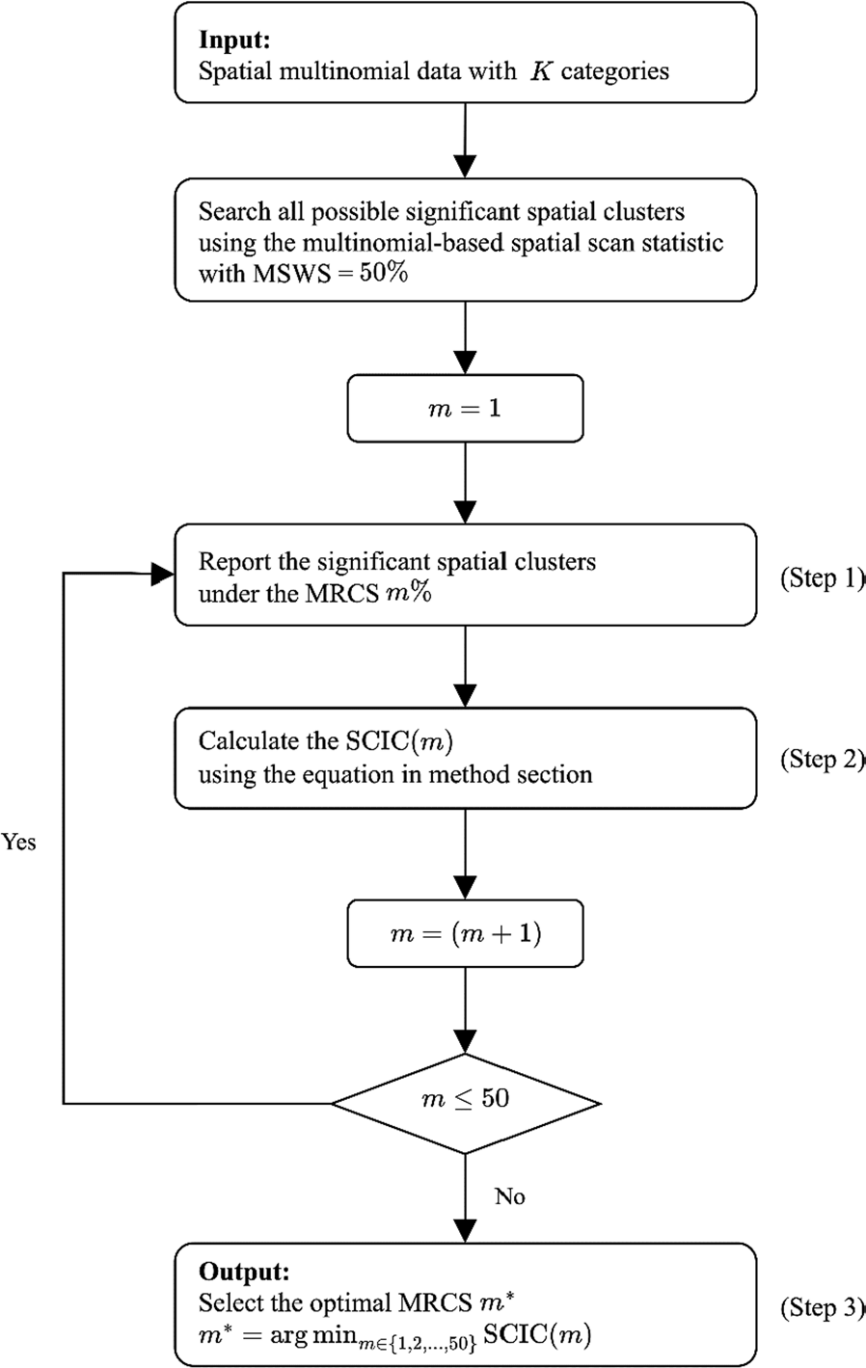
The flowchart of the proposed method

Figure 1 illustrates the flowchart of the proposed method.

### Elbow method, MCS-P, and MCHS-P

For the Poisson-based spatial scan statistic, optimization criteria such as the elbow method [22], the maximum clustering set–proportion (MCS-P) [23], and the maximum clustering heterogeneous set-proportion (MCHS-P) [24] have been proposed to determine the optimal value of MRCS or MSWS. Since these methods are likelihood-based optimization criteria, we have adapted them to the multinomial model in order to evaluate and compare their performance with our proposed approaches. The logical order is the same as the SCICs, with the only difference being the measure being calculated. It’s important to emphasize that we should consider optimizing MRCS, not MSWS, to avoid the multiple testing problem, as noted by Han et al. [17].

The elbow method [30] is commonly employed in unsupervised learning to determine the optimal number of clusters by identifying the elbow point. In the context of selecting the optimal MRCS value, Meysami et al. [22] proposed an optimization criterion for the Poisson model by adopting the method for finding the optimal elbow point as suggested by Delgado et al. [31]. We employ the method for the multinomial model by calculating the negative sum of the likelihood ratio test (LRT) statistic values over all ${J}_{m}$ significant clusters for each $m$ as

$$-LRT\left(m\right)=-\sum _{j=1}^{{J}_{m}}{\lambda }_{{Z}_{j}^{\left(m\right)}}$$

where ${\lambda }_{{Z}_{j}^{\left(m\right)}}$ denotes the LRT statistics value for the ${j}{\text{th}}$ significant cluster ${Z}_{j}^{\left(m\right)}$ ($j$= 1, …, ${J}_{m}$). If no significant cluster is present, use the maximum LRT statistic. The elbow plot is constructed by connecting the points ($m, -LRT\left(m\right)$) for $m$= 1, …, 50. For each $m$, we calculate the orthogonal distance between each point ($m, -LRT(m)$) and the line connecting the first and last points. The optimal MRCS is the one that maximizes this orthogonal distance.

Ma et al. [23] proposed the maximum clustering set–proportion (MCS-P) as an optimization criterion to determine the optimal value of the MSWS for the Poisson-based spatial scan statistic. This criterion assumes that all identified significant clusters are homogeneous clusters with the same relative risks. However, considering the issue of multiple testing, analyzing the data multiple times with different MSWS values to select the best result might not be appropriate. In our study, we adapt the MCS-P criterion to the multinomial model and utilize it to select the optimal MRCS, while keeping the MSWS value fixed at 50%. To apply the MCS-P to the multinomial model, we first define the union cluster set ${Z}_{A}^{\left(m\right)}$ by merging all ${J}_{m}$ clusters for each $m$ as

$${Z}_{A}^{\left(m\right)}={\bigcup }_{j=1}^{{J}_{m}}{Z}_{j}^{\left(m\right)}$$

where ${Z}_{j}^{\left(m\right)}$ is the ${j}{\text{th}}$ detected significant cluster ($j$= 1, …, ${J}_{m}$). Then, we calculate the union log-likelihood ratio (LLR) test statistic $log{\lambda }_{{Z}_{A}^{\left(m\right)}}$ given the union cluster set ${Z}_{A}^{\left(m\right)}$ as

$$log{\lambda }_{{Z}_{A}^{\left(m\right)}}=\sum _{k}\left\{\sum _{i\in {Z}_{A}^{\left(m\right)}}{c}_{ik}\cdot log\left(\frac{\sum _{i\in {Z}_{A}^{\left(m\right)}}{c}_{ik}}{\sum _{i\in {Z}_{A}^{\left(m\right)}}{c}_{i}}\right)+\left({C}_{k}-\sum _{i\in {Z}_{A}^{\left(m\right)}}{c}_{ik}\right)\cdot log\left(\frac{{C}_{k}-\sum _{i\in {Z}_{A}^{\left(m\right)}}{c}_{ik}}{C-\sum _{i\in {Z}_{A}^{\left(m\right)}}{c}_{i}}\right)\right\}+\sum _{k}{C}_{k}\cdot log\left(\frac{{C}_{k}}{C}\right)$$

where ${c}_{ik}$, ${C}_{k}$, and $C$ were as defined previously and ${c}_{i}$ is the number of cases inside the region $i$. The optimal MRCS is the one that maximizes the union LLR test statistic $log{\lambda }_{{Z}_{A}^{\left(m\right)}}$.

Considering the possibility of detected significant clusters being heterogeneous with varying relative risks, Wang et al. [24] introduced the maximum clustering heterogeneous set-proportion (MCHS-P) as an optimization criterion to determine the optimal value of the MSWS. As previously discussed, we employ the MCS-P criterion in the multinomial model and utilize it to select the optimal MRCS, while maintaining a fixed MSWS value of 50%. For each $m$, we define the heterogeneous cluster set ${Z}_{B}^{\left(m\right)}$ by merging ${J}_{m}$ detected significant clusters into ${W}_{m} ({W}_{m}\le {J}_{m})$ merged clusters according to their spatial contiguity.

$${Z}_{B}^{\left(m\right)}=\left\{{Z}_{{B}_{1}}^{\left(m\right)}, {\ldots , Z}_{{B}_{{W}_{m}}}^{\left(m\right)}\right\}$$

Then we calculate the union LLR test statistic $log{\lambda }_{{Z}_{B}^{\left(m\right)}}$ given the heterogeneous cluster set ${Z}_{B}^{\left(m\right)}$ as

$$log{\lambda }_{{Z}_{B}^{\left(m\right)}}=\sum _{k}\left\{\sum _{i\in {Z}_{{B}_{1}}^{\left(m\right)}}{c}_{ik}\cdot log\left(\frac{\sum _{i\in {Z}_{{B}_{1}}^{\left(m\right)}}{c}_{ik}}{\sum _{i\in {Z}_{{B}_{1}}^{\left(m\right)}}{c}_{i}}\right)+\cdots +\sum _{i\in {Z}_{{B}_{{W}_{m}}}^{\left(m\right)}}{c}_{ik}\cdot log\left(\frac{\sum _{i\in {Z}_{{B}_{{W}_{m}}}^{\left(m\right)}}{c}_{ik}}{\sum _{i\in {Z}_{{B}_{{W}_{m}}}^{\left(m\right)}}{c}_{i}}\right)+\left({C}_{k}-\sum _{i\in {Z}_{B}^{\left(m\right)}}{c}_{ik}\right)\cdot log\left(\frac{{C}_{k}-\sum _{i\in {Z}_{B}^{\left(m\right)}}{c}_{ik}}{C-\sum _{i\in {Z}_{B}^{\left(m\right)}}{c}_{i}}\right)\right\}+\sum _{k}{C}_{k}\cdot log\left(\frac{{C}_{k}}{C}\right)$$

The optimal MRCS is the one that maximizes the union LLR test statistic $log{\lambda }_{{Z}_{B}^{\left(m\right)}}$.

### Simulation study

**Fig. 2:**
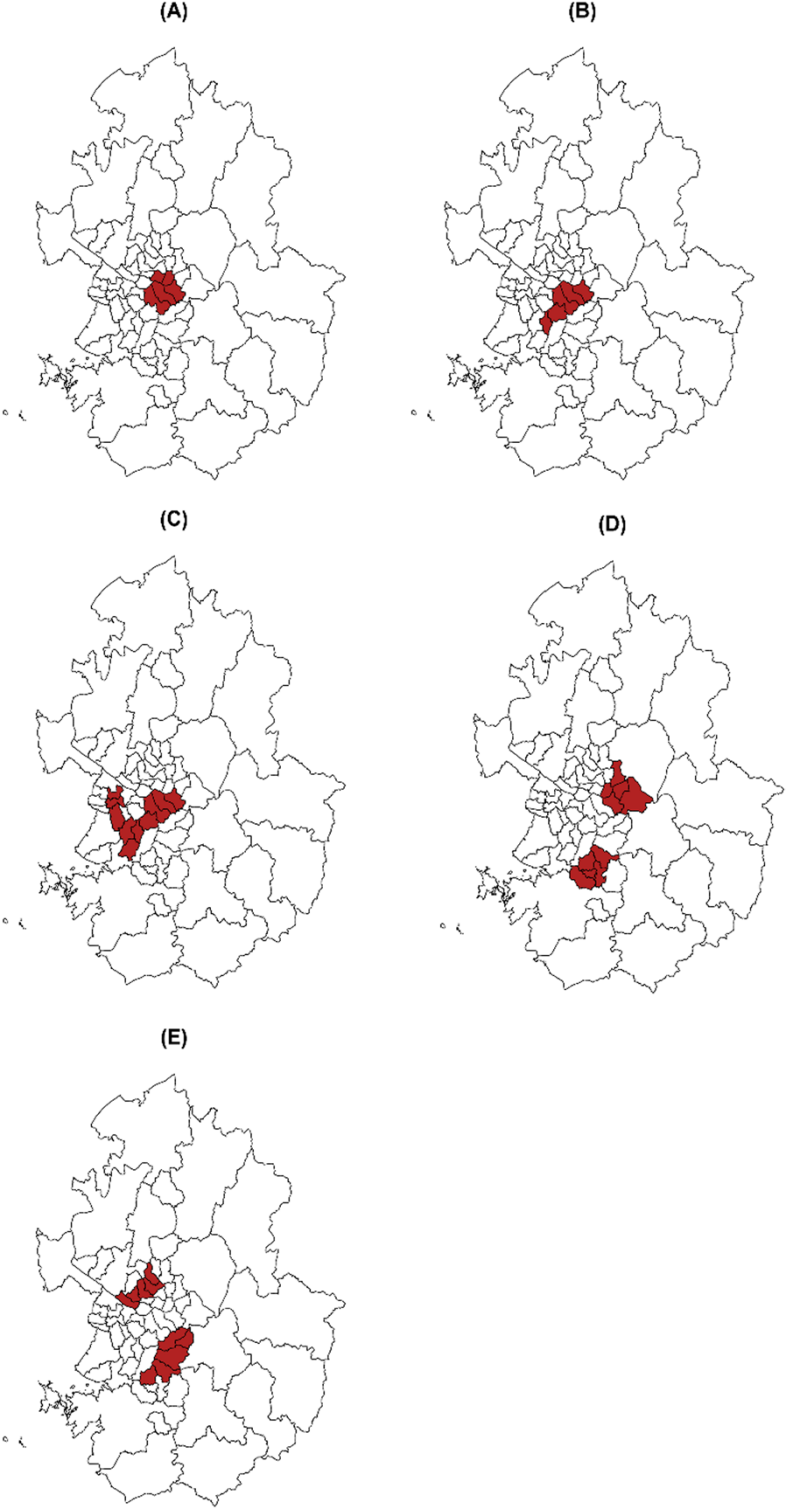
True cluster models in the simulation study

We conducted a simulation study to evaluate the performance of the proposed method for the multinomial model in comparison to other existing methods. The study region comprised Seoul and Gyeonggi Province in South Korea, consisting of 69 districts. For the simulation, we considered five different true cluster models as depicted in Fig. 2. True cluster models (A) and (B) represented one circular-shaped and one elliptical-shaped true cluster, respectively, each consisting of 5 districts, which accounted for 8% of the entire study region. True cluster model (C) depicted one irregular-shaped true cluster with 10 districts, representing 15% of the entire study region. True cluster models (D) and (E) assumed two circular-shaped and two elliptical-shaped true clusters, respectively, each consisting of 5 districts.

For each true cluster model, we considered various scenarios of the alternative hypothesis, assuming four categories. The parameter setting for the alternative hypothesis was adopted from a previous study [6]. The null hypothesis was set to equal probabilities of 0.25 for each of four categories. In the previous study [6], several different alternative hypotheses were used to evaluate the multinomial-based spatial scan statistic and successfully showed that the multinomial-based spatial scan statistic worked well under those hypotheses. In this study, we aimed to assess a method for optimizing the MRCS for the multinomial-based spatial scan statistic and believe that it would be good to evaluate its performance under the same hypotheses. Furthermore, because the alternative hypotheses satisfy the likelihood ratio ordering, we were also able to evaluate the performance of the ordinal model [3]. For the true cluster models with two clusters, we included heterogeneous settings where different alternative hypotheses were assigned to each cluster, as well as homogeneous settings where the same alternative hypotheses were applied to both clusters. This allowed us to examine the performance of the proposed method in more plausible heterogeneous settings, where the relative risks of each category differ between the two clusters. We considered four alternative hypotheses for the true cluster models with one cluster and two homogeneous clusters, as well as three alternative hypotheses for the true cluster models with two heterogeneous clusters. This resulted in a total of 26 scenarios considered in combination. Table 1 presents the simulation scenarios for the true cluster model along with their respective alternative hypotheses.

**Table 1:** Simulation scenarios for the true cluster model and alternative hypothesis

Setting	True cluster model	Alternative hypothesis^a^
Single cluster	(A) One circular-shaped cluster (8%) (B) One elliptic-shaped cluster (8%) (C) One irregular-shaped cluster (15%)	(1) p^(1)^ = (0.05, 0.15, 0.35, 0.45)
		(2) p^(1)^ = (0.05, 0.25, 0.25, 0.45)
		(3) p^(1)^ = (0.10, 0.10, 0.40, 0.40)
		(4) p^(1)^ = (0.15, 0.15, 0.15, 0.55)
Two homogeneous clusters	(D) Two circular-shaped clusters (8% each) (E) Two elliptic-shaped clusters (8% each)	(1) p^(1)^ = p^(2)^ = (0.05, 0.15, 0.35, 0.45)
		(2) p^(1)^ = p^(2)^ = (0.05, 0.25, 0.25, 0.45)
		(3) p^(1)^ = p^(2)^ = (0.10, 0.10, 0.40, 0.40)
		(4) p^(1)^ = p^(2)^ = (0.15, 0.15, 0.15, 0.55)
Two heterogeneous clusters	(D) Two circular-shaped clusters (8% each) (E) Two elliptic-shaped clusters (8% each)	(5) p^(1)^ = (0.05, 0.15, 0.35, 0.45), p^(2)^ = (0.05, 0.25, 0.25, 0.45)
		(6) p^(1)^ = (0.05, 0.15, 0.35, 0.45), p^(2)^ = (0.10, 0.10, 0.40, 0.40)
		(7) p^(1)^= (0.05, 0.15, 0.35, 0.45), p^(2)^ = (0.15, 0.15, 0.15, 0.55)

^a^p^(1)^ is for cluster 1 and p^(2)^ is for cluster 2; p^(0)^ = (0.25, 0.25, 0.25, 0.25) was assumed for the remaining areas

Under each scenario, we generated 1000 datasets, each containing 1000 cases distributed among four categories. For each data set, we repeatedly identified clusters by varying the MRCS values. In SaTScan™, the MRCS value was set to 1%, 2%, 3%, 4%, 5%, 6%, 8%, 10%, 12%, 15%, 20%, 25%, 30%, 35%, 40%, 45%, and 50%. As SaTScan™ provides Gini coefficient values for these 17 candidate MRCS values in the Bernoulli and Poisson models, we computed the SCICs, Gini coefficient (for the ordinal model), Elbow method, MCS-P and MCHS-P values for these 17 candidate MRCS values for consistency. Then, we compared the clusters reported by each method using the optimal MRCS selected, with the true clusters. Regarding the scanning window shape, we presented the simulation results obtained when using the elliptical windows as the main results because Kulldorff et al. [32] found that the spatial scan statistic with elliptic windows exhibited good performance in terms of the power when the shape of the true cluster is elliptical or circular.

Over 1000 randomly generated datasets, we recorded the frequency at which each candidate MRCS value was selected as the optimal MRCS for each method. To compare the performance of the proposed method with other existing methods and default setting (MRCS value of 50%), we used sensitivity, positive predicted value (PPV) and misclassification as the performance measures, as per a previous study [33]. Sensitivity represents the proportion of correctly identified districts within the true cluster, while PPV represents the proportion of correctly identified districts within the detected cluster. A method with higher values of these measures indicates greater precision in identifying the true cluster. A lower sensitivity means that the method failed to identify some districts that belong to the true cluster. A lower PPV means that the method identified some districts that do not belong to the true cluster. Misclassification indicates the proportion of incorrectly identified districts within the true or detected cluster. Higher sensitivity and PPV values, along with lower misclassification values, indicate better performance in accurately identifying clusters. We calculated the average sensitivity, PPV, and misclassification over 1000 simulated datasets for two sets of MRCS values: (1) those selected by SCIC_1_, SCIC_2_, Gini coefficient (only for the ordinal model), Elbow method, MCS-P, and MCHS-P, and (2) the default value of 50%. The simulation was conducted using SaTScan™ version 10.0 and R software version 4.0.2, employing the ‘rsatscan’ package [34].

## Results

### Simulation study results

Tables 2, 3, 4, 5 present the simulation results for cluster model (B). The other results are provided in Additional file 1. For cluster models (A), (B), (D), and (E), all five methods most often selected the optimal MRCS value equal to the size of the true cluster from the 17 candidate MRCS values, regardless of the alternative hypothesis scenario. For cluster model (C) of irregular-shaped cluster, all five methods most often chose an optimal MRCS value of 12%, which is smaller than the size of the true cluster (30%), irrespective of the alternative hypothesis scenario. When using the optimal MRCS value instead of the default setting, the methods tend to report multiple informative smaller clusters instead of reporting a single larger cluster that contains the true irregular cluster.

**Table 2:** Multinomial model: simulation results for the true cluster model (B) and alternative hypothesis (1) using elliptical windows

		Maximum reported cluster size (MRCS)																		Default setting
		1%	2%	3%	4%	5%	6%	8%	10%	12%	15%	20%	25%	30%	35%	40%	45%	50%	Overall	
SCIC_1_	Freq^a^	0	2	0	9	32	61	**547**	123	85	39	20	4	3	1	0	0	1	927	927
	Sen^b^	NA	0.200	NA	0.444	0.556	0.695	**0.897**	0.927	0.972	0.949	0.890	0.850	0.933	0.000	NA	NA	1.000	0.878	0.888
	PPV^c^	NA	1.000	NA	1.000	0.927	0.939	**0.965**	0.773	0.655	0.519	0.356	0.237	0.254	0.000	NA	NA	0.088	0.869	0.803
	Mis^d^	NA	0.058	NA	0.040	0.035	0.027	**0.011**	0.026	0.043	0.071	0.133	0.210	0.208	0.406	NA	NA	0.754	0.026	0.044
SCIC_2_	Freq	0	2	0	13	32	66	**521**	115	86	39	24	8	10	2	4	2	3	927	927
	Sen	NA	0.200	NA	0.538	0.594	0.694	**0.899**	0.920	0.972	0.959	0.892	0.900	0.860	0.500	0.800	1.000	0.933	0.878	0.888
	PPV	NA	1.000	NA	0.981	0.947	0.933	**0.967**	0.770	0.649	0.513	0.339	0.233	0.195	0.081	0.126	0.132	0.105	0.850	0.803
	Mis	NA	0.058	NA	0.035	0.032	0.027	**0.010**	0.027	0.044	0.074	0.142	0.223	0.278	0.391	0.420	0.478	0.594	0.034	0.044
Elbow	Freq	0	1	2	24	45	69	**531**	117	80	27	20	5	4	1	1	0	0	927	927
	Sen	NA	0.200	0.800	0.692	0.667	0.707	**0.898**	0.916	0.968	0.956	0.850	0.920	0.850	0.000	0.600	NA	NA	0.874	0.888
	PPV	NA	1.000	1.000	0.976	0.944	0.928	**0.958**	0.764	0.651	0.501	0.282	0.216	0.199	0.000	0.094	NA	NA	0.868	0.803
	Mis	NA	0.058	0.014	0.024	0.027	0.027	**0.958**	0.028	0.044	0.079	0.174	0.249	0.272	0.406	0.449	NA	NA	0.027	0.044
MCS-P	Freq	0	0	3	25	49	60	**486**	129	77	56	27	5	5	3	2	0	0	927	927
	Sen	NA	NA	0.333	0.576	0.624	0.677	**0.893**	0.929	0.977	0.971	0.933	0.880	1.000	0.667	1.000	NA	NA	0.872	0.888
	PPV	NA	NA	0.333	0.939	0.861	0.916	**0.973**	0.787	0.679	0.555	0.411	0.285	0.271	0.145	0.192	NA	NA	0.856	0.803
	Mis	NA	NA	0.068	0.035	0.034	0.029	**0.010**	0.023	0.036	0.059	0.104	0.168	0.197	0.309	0.304	NA	NA	0.026	0.044
MCHS-P	Freq	0	0	2	19	44	60	**484**	131	80	58	32	6	6	3	2	0	0	927	927
	Sen	NA	NA	0.800	0.674	0.677	0.690	**0.895**	0.919	0.975	0.972	0.925	0.867	0.967	0.667	1.000	NA	NA	0.882	0.888
	PPV	NA	NA	1.000	1.000	0.910	0.910	**0.968**	0.770	0.663	0.549	0.390	0.273	0.257	0.145	0.192	NA	NA	0.848	0.803
	Mis	NA	NA	0.014	0.024	0.029	0.029	**0.010**	0.027	0.040	0.061	0.115	0.179	0.208	0.309	0.304	NA	NA	0.028	0.044

^a^Freq: frequency

^b^Sen: sensitivity

^c^PPV: positive predictive value

^d^Mis: misclassification

Sensitivity, PPV, and misclassification rate at the most frequently selected optimal MRCS are shown in bold

**Table 3:** Multinomial model: simulation results for the true cluster model (B) and alternative hypothesis (2) using elliptical windows

		Maximum reported cluster size (MRCS)																		Default setting
		1%	2%	3%	4%	5%	6%	8%	10%	12%	15%	20%	25%	30%	35%	40%	45%	50%	Overall	
SCIC_1_	Freq^a^	0	2	4	11	43	54	**403**	132	72	53	25	12	7	3	1	1	3	826	826
	Sen^b^	NA	0.100	0.350	0.400	0.567	0.641	**0.890**	0.923	0.961	0.970	0.840	0.817	1.000	0.933	1.000	1.000	1.000	0.862	0.876
	PPV^c^	NA	0.500	1.000	0.879	0.927	0.894	**0.959**	0.763	0.651	0.545	0.348	0.256	0.275	0.203	0.185	0.172	0.139	0.824	0.752
	Mis^d^	NA	0.072	0.047	0.049	0.035	0.032	**0.011**	0.028	0.043	0.065	0.130	0.200	0.193	0.271	0.319	0.348	0.469	0.035	0.060
SCIC_2_	Freq	0	2	3	10	40	53	**392**	130	68	51	26	15	11	8	4	5	8	826	826
	Sen	NA	0.100	0.333	0.380	0.585	0.649	**0.890**	0.932	0.956	0.969	0.838	0.853	0.945	0.825	0.800	0.960	0.975	0.865	0.876
	PPV	NA	0.500	1.000	0.867	0.938	0.910	**0.960**	0.772	0.646	0.544	0.340	0.240	0.244	0.155	0.131	0.146	0.118	0.805	0.752
	Mis	NA	0.072	0.048	0.051	0.034	0.031	**0.011**	0.027	0.044	0.066	0.134	0.220	0.223	0.346	0.402	0.414	0.567	0.046	0.060
Elbow	Freq	0	1	3	11	51	57	**412**	129	70	41	20	13	8	4	3	1	0	824	826
	Sen	NA	0.000	0.333	0.509	0.600	0.653	**0.891**	0.927	0.954	0.966	0.800	0.877	0.850	0.900	0.467	1.000	NA	0.859	0.876
	PPV	NA	0.000	1.000	0.879	0.913	0.908	**0.955**	0.761	0.648	0.539	0.312	0.240	0.219	0.164	0.083	0.161	NA	0.828	0.752
	Mis	NA	0.087	0.048	0.041	0.034	0.031	**0.012**	0.029	0.044	0.067	0.147	0.224	0.250	0.355	0.357	0.377	NA	0.035	0.060
MCS-P	Freq	0	2	5	11	46	51	**378**	121	74	60	36	18	11	3	5	3	2	826	826
	Sen	NA	0.000	0.280	0.473	0.565	0.655	**0.886**	0.924	0.962	0.967	0.878	0.911	0.855	0.933	0.960	1.000	1.000	0.862	0.876
	PPV	NA	0.000	0.800	0.788	0.870	0.899	**0.961**	0.775	0.671	0.551	0.380	0.302	0.235	0.203	0.187	0.167	0.155	0.802	0.752
	Mis	NA	0.087	0.058	0.046	0.038	0.031	**0.011**	0.025	0.037	0.062	0.115	0.159	0.213	0.271	0.304	0.362	0.399	0.038	0.060
MCHS-P	Freq	0	2	4	9	47	50	**376**	123	76	60	35	19	12	3	5	3	2	826	826
	Sen	NA	0.000	0.350	0.556	0.591	0.652	**0.887**	0.922	0.961	0.970	0.886	0.884	0.867	0.933	0.960	1.000	1.000	0.866	0.876
	PPV	NA	0.000	1.000	0.807	0.891	0.903	**0.961**	0.767	0.662	0.548	0.383	0.287	0.234	0.203	0.187	0.167	0.155	0.800	0.752
	Mis	NA	0.087	0.047	0.042	0.036	0.031	**0.011**	0.027	0.040	0.063	0.114	0.175	0.217	0.271	0.304	0.362	0.399	0.039	0.060

^a^Freq: frequency

^b^Sen: sensitivity

^c^PPV: positive predictive value

^d^Mis: misclassification

Sensitivity, PPV, and misclassification rate at the most frequently selected optimal MRCS are shown in bold

**Table 4:** Multinomial model: simulation results for the true cluster model (B) and alternative hypothesis (3) using elliptical windows

		Maximum reported cluster size (MRCS)																		Default setting
		1%	2%	3%	4%	5%	6%	8%	10%	12%	15%	20%	25%	30%	35%	40%	45%	50%	Overall	
SCIC_1_	Freq^a^	0	1	4	10	46	52	**424**	117	74	44	27	9	2	4	1	3	1	819	819
	Sen^b^	NA	0.200	0.250	0.380	0.552	0.650	**0.881**	0.916	0.924	0.973	0.874	0.911	1.000	0.750	0.400	0.733	0.800	0.850	0.868
	PPV^c^	NA	1.000	0.750	0.917	0.889	0.911	**0.954**	0.756	0.620	0.532	0.336	0.270	0.278	0.173	0.080	0.094	0.105	0.827	0.755
	Mis^d^	NA	0.058	0.062	0.048	0.039	0.031	**0.012**	0.029	0.050	0.068	0.145	0.193	0.188	0.279	0.377	0.531	0.507	0.036	0.055
SCIC_2_	Freq	0	1	4	13	46	52	**416**	112	73	41	28	11	4	6	5	6	1	819	819
	Sen	NA	0.200	0.250	0.523	0.578	0.654	**0.878**	0.913	0.921	0.976	0.857	0.909	1.000	0.833	0.880	0.833	0.800	0.850	0.868
	PPV	NA	1.000	0.750	0.946	0.903	0.900	**0.951**	0.754	0.618	0.533	0.322	0.251	0.236	0.161	0.140	0.112	0.105	0.814	0.755
	Mis	NA	0.058	0.062	0.037	0.036	0.031	**0.013**	0.030	0.051	0.068	0.152	0.212	0.246	0.341	0.394	0.493	0.507	0.042	0.055
Elbow	Freq	0	1	5	18	56	57	**429**	105	73	33	21	11	2	3	3	2	0	819	819
	Sen	NA	0.200	0.320	0.611	0.632	0.653	**0.882**	0.897	0.915	0.964	0.800	0.964	1.000	0.667	0.800	0.600	NA	0.844	0.868
	PPV	NA	1.000	0.800	0.961	0.899	0.891	**0.949**	0.738	0.609	0.513	0.295	0.262	0.225	0.118	0.113	0.094	NA	0.830	0.755
	Mis	NA	0.058	0.055	0.030	0.033	0.032	**0.013**	0.032	0.053	0.074	0.163	0.208	0.268	0.372	0.473	0.449	NA	0.036	0.055
MCS-P	Freq	0	3	5	13	50	56	**384**	104	80	53	41	14	7	5	2	2	0	819	819
	Sen	NA	0.133	0.160	0.492	0.616	0.632	**0.874**	0.919	0.940	0.962	0.927	0.957	0.971	0.800	0.700	0.600	NA	0.850	0.868
	PPV	NA	0.667	0.400	0.808	0.844	0.879	**0.955**	0.774	0.650	0.545	0.400	0.317	0.253	0.180	0.133	0.094	NA	0.804	0.755
	Mis	NA	0.068	0.078	0.046	0.036	0.034	**0.012**	0.025	0.042	0.063	0.108	0.153	0.211	0.278	0.348	0.449	NA	0.037	0.055
MCHS-P	Freq	0	1	3	11	47	54	**381**	107	81	54	46	17	7	5	3	2	0	819	819
	Sen	NA	0.200	0.267	0.655	0.647	0.644	**0.873**	0.920	0.936	0.967	0.904	0.953	0.971	0.800	0.800	0.600	NA	0.861	0.868
	PPV	NA	1.000	0.667	0.982	0.888	0.895	**0.950**	0.762	0.645	0.536	0.375	0.303	0.253	0.180	0.144	0.094	NA	0.799	0.755
	Mis	NA	0.058	0.063	0.026	0.033	0.032	**0.013**	0.027	0.044	0.066	0.124	0.166	0.211	0.278	0.353	0.449	NA	0.039	0.055

^a^Freq: frequency

^b^Sen: sensitivity

^c^PPV: positive predictive value

^d^Mis: misclassification

Sensitivity, PPV, and misclassification rate at the most frequently selected optimal MRCS are shown in bold

**Table 5:** Multinomial model: simulation results for the true cluster model (B) and alternative hypothesis (4) using elliptical windows

		Maximum reported cluster size (MRCS)																		Default setting
		1%	2%	3%	4%	5%	6%	8%	10%	12%	15%	20%	25%	30%	35%	40%	45%	50%	Overall	
SCIC_1_	Freq^a^	0	6	8	21	32	69	**428**	111	72	47	28	12	7	6	1	1	1	850	850
	Sen^b^	NA	0.167	0.325	0.381	0.519	0.661	**0.884**	0.901	0.942	0.962	0.914	0.800	0.886	0.633	0.800	1.000	1.000	0.839	0.860
	PPV^c^	NA	0.833	0.813	0.921	0.863	0.938	**0.950**	0.750	0.636	0.525	0.380	0.256	0.198	0.139	0.160	0.161	0.082	0.823	0.733
	Mis^d^	NA	0.063	0.054	0.048	0.042	0.028	**0.013**	0.030	0.046	0.070	0.119	0.188	0.273	0.324	0.319	0.377	0.812	0.037	0.068
SCIC_2_	Freq	0	7	8	26	35	69	**401**	101	65	44	27	21	16	11	5	6	8	850	850
	Sen	NA	0.171	0.350	0.446	0.549	0.658	**0.884**	0.895	0.935	0.959	0.911	0.819	0.900	0.727	0.800	0.900	1.000	0.835	0.860
	PPV	NA	0.857	0.813	0.913	0.864	0.938	**0.951**	0.744	0.634	0.513	0.363	0.228	0.191	0.146	0.130	0.131	0.114	0.792	0.733
	Mis	NA	0.062	0.053	0.045	0.040	0.028	**0.012**	0.032	0.046	0.074	0.135	0.222	0.289	0.331	0.406	0.442	0.576	0.054	0.068
Elbow	Freq	0	5	9	35	51	74	**423**	99	65	39	18	16	10	4	1	1	0	850	850
	Sen	NA	0.160	0.422	0.560	0.608	0.654	**0.887**	0.905	0.935	0.949	0.911	0.800	0.800	0.750	0.800	1.000	NA	0.833	0.860
	PPV	NA	0.800	0.833	0.914	0.869	0.932	**0.947**	0.755	0.638	0.493	0.320	0.216	0.164	0.173	0.160	0.161	NA	0.829	0.733
	Mis	NA	0.064	0.047	0.037	0.036	0.029	**0.013**	0.029	0.045	0.081	0.167	0.232	0.310	0.283	0.319	0.377	NA	0.038	0.068
MCS-P	Freq	0	4	6	25	41	66	**380**	106	86	56	39	13	12	9	4	3	0	850	850
	Sen	NA	0.100	0.333	0.568	0.600	0.630	**0.878**	0.900	0.944	0.971	0.949	0.923	0.800	0.867	0.950	1.000	NA	0.848	0.860
	PPV	NA	0.500	0.833	0.921	0.878	0.908	**0.952**	0.761	0.648	0.545	0.424	0.308	0.220	0.194	0.184	0.163	NA	0.801	0.733
	Mis	NA	0.072	0.053	0.035	0.036	0.032	**0.013**	0.028	0.042	0.062	0.099	0.156	0.220	0.272	0.308	0.372	NA	0.039	0.068
MCHS-P	Freq	0	3	5	21	41	64	**372**	106	86	59	41	18	15	12	4	3	0	850	850
	Sen	NA	0.133	0.320	0.562	0.610	0.644	**0.882**	0.900	0.942	0.966	0.946	0.889	0.827	0.817	0.950	1.000	NA	0.853	0.860
	PPV	NA	0.667	0.800	0.915	0.882	0.926	**0.950**	0.758	0.644	0.529	0.409	0.276	0.213	0.178	0.184	0.163	NA	0.787	0.733
	Mis	NA	0.068	0.055	0.036	0.035	0.030	**0.013**	0.029	0.043	0.068	0.107	0.187	0.236	0.292	0.308	0.372	NA	0.044	0.068

^a^Freq: frequency

^b^Sen: sensitivity

^c^PPV: positive predictive value

^d^Mis: misclassification

Sensitivity, PPV, and misclassification rate at the most frequently selected optimal MRCS are shown in bold

The proposed methods consistently exhibited higher sensitivity and positive predictive value (PPV) at the most frequently selected MRCS value than the default setting. Additionally, the rate of misclassification was much lower. The overall sensitivity of the proposed methods was slightly lower than that of the default setting. However, the overall PPV was higher than that of the default setting. Across all scenarios, it appears that all five methods yielded similar overall detection accuracy in terms of sensitivity, PPV, and misclassification. The overall sensitivity of SCIC_1_ was comparable to SCIC_2_, while the overall PPV of SCIC_1_ was slightly higher than that of SCIC_2_.

The simulation results for the ordinal model are provided in Additional file 2: Tables A23–A48). The proposed methods and the other three methods for the ordinal model have similar trends in simulation results for the multinomial model. The sensitivity and PPV of SCIC_1_ and SCIC_2_ at the most often selected MRCS value were higher than those of the default setting. The overall PPV of the proposed methods was higher than that of the default setting, while the sensitivity was comparable. Additionally, the misclassification rate was consistently lower. We noticed that the overall sensitivity of the SCIC_2_ was slightly higher than that of the SCIC_1_ in cluster models (D) and (E), which involve two clusters. The Gini coefficient exhibited higher sensitivity and PPV, and lower misclassification at the most often chosen MRCS value, but its overall performance was quite similar to that of the default setting.

### Application to Korea Community Health Survey data

We used the Korea Community Health Survey (KCHS) data to illustrate the usefulness of the proposed method. The KCHS is an annual survey conducted by the Korea Disease Control and Prevention Agency since 2008 to gather community-based health statistics. This survey was carried out across 253 community health centers, covering various aspects such as health behaviors, self-reported health indicators, and demographic characteristics. For our analysis, we used the ‘reason for starting to drink’ as the nominal categorical variable from the 2019 KCHS data. Subjects who had never consumed alcohol were excluded. The ‘reason for starting to drink’ was categorized into four groups: (1) recommended by people, (2) out of curiosity, (3) to promote friendship, and (4) other reasons. It would be valuable to examine the spatial autocorrelation to assess whether this outcome variable exhibits inherent spatial dependency. However, based on the literature search conducted thus far, it seems that there is no established method for calculating spatial autocorrelation in the context of multinomial data. The results of the spatial cluster detection analysis might provide insights into spatial autocorrelation. Using the multinomial-based spatial scan statistic with elliptical windows, we searched for regions in Seoul and Gyeonggi province that exhibited distinct distributions of the ‘reason for starting to drink’ among males in their 20 and 30 s.

**Fig. 3:**
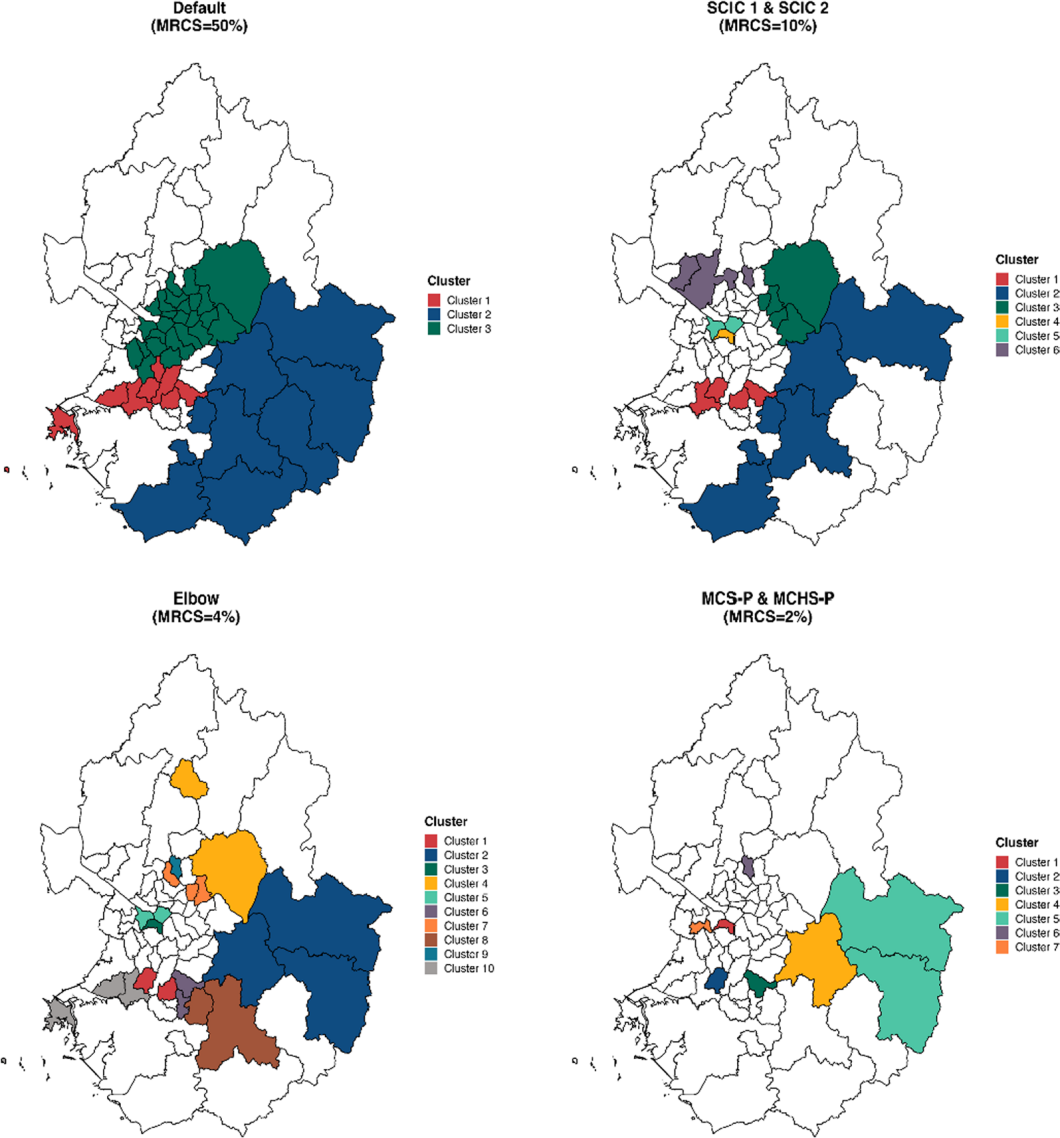
A map of the significant spatial clusters identified using the multinomial-based spatial scan statistic with elliptical windows at the MRCS suggested by (1) default setting, (2) SCIC_1_, (3) SCIC_2_, (4) elbow method, (5) MCS-P, and (6) MCHS-P

The reported clusters differed depending on the method used to optimize the MRCS value. Figure 3 shows a map of the significant spatial clusters reported by each method. A summary of those clusters is presented in Table 6. The SCIC_1_ and SCIC_2_ methods selected an optimal MRCS of 10%, which is smaller than the default setting. When using the default setting, three large clusters were reported. In contrast, the proposed methods identified six smaller clusters that seem to carry more meaningful information. Cluster 1 reported using the SCICs belongs to cluster 1 reported using the default setting. Similarly, cluster 2 reported using SCICs belongs to cluster 2 reported using the default setting. Clusters 3, 4, and 5 reported using the SCICs belong to cluster 3 reported using the default setting. The proposed methods seemed to reveal more meaningful smaller clusters that were not identified by the default setting. It is worth noting that cluster 4 reported using the SCICs was a hidden smaller cluster with the highest relative risk (RR) in category 3, rather than in category 1 as cluster 3 identified in the default setting. Additionally, the proposed methods reported another regions as cluster 6, which went unnoticed by the default setting.

**Table 6:** A summary of the significant spatial clusters identified using the multinomial-based spatial scan statistic with elliptical windows at the MRCS suggested by (1) default setting, (2) SCIC_1_, (3) SCIC_2_, (4) elbow method, (5) MCS-P, and (6) MCHS-P

	MRCS	Cluster	Districts^a^	LLR^b^	p-value	Obs^c^	RR^d^ of each category
Default	50	1	7	48.655	< 0.001	933	(0.68, 1.24, 1.45, 1.16)
		2	10	38.363	< 0.001	1200	(0.98, 1.60, 0.71, 0.91)
		3	25	40.119	< 0.001	3096	(1.19, 0.70, 0.87, 1.10)
SCIC_1_, SCIC_2_	10	1	4	50.148	< 0.001	501	(0.57, 1.24, 1.59, 1.47)
		2	6	37.323	< 0.001	798	(0.91, 1.76, 0.72, 0.96)
		3	5	28.589	< 0.001	694	(1.30, 0.77, 0.67, 0.75)
		4	1	19.396	< 0.001	126	(0.87, 0.27, 1.83, 0.43)
		5	2	19.119	< 0.001	237	(1.40, 0.69, 0.55, 0.61)
		6	3	17.032	0.015	385	(0.76, 1.00, 1.50, 0.80)
Elbow	4	1	2	26.842	< 0.001	240	(0.55, 1.25, 1.67, 1.07)
		2	3	22.751	< 0.001	274	(0.83, 2.01, 0.72, 0.87)
		3	1	19.396	< 0.001	126	(0.87, 0.27, 1.83, 0.43)
		4	2	23.128	< 0.001	318	(1.38, 0.80, 0.53, 0.63)
		5	2	19.119	< 0.001	237	(1.40, 0.69, 0.55, 0.61)
		6	2	17.539	0.002	269	(0.75, 0.72, 1.57, 1.46)
		7	3	15.558	0.016	322	(1.28, 0.98, 0.63, 0.45)
		8	2	13.309	0.017	220	(1.12, 1.45, 0.49, 1.09)
		9	1	12.712	0.025	108	(0.65, 0.69, 1.82, 1.19)
		10	2	12.000	0.046	299	(0.72, 1.44, 1.20, 1.18)
MCS-P, MCHS-P	2	1	1	19.396	< 0.001	126	(0.87, 0.27, 1.83, 0.43)
		2	1	19.383	< 0.001	130	(0.51, 1.15, 1.83, 0.99)
		3	1	19.061	< 0.001	116	(0.48, 1.04, 1.73, 2.09)
		4	1	14.115	0.011	139	(0.67, 2.05, 0.92, 1.06)
		5	2	12.870	0.022	135	(1.00, 1.89, 0.51, 0.68)
		6	1	12.712	0.025	108	(0.65, 0.69, 1.82, 1.19)
		7	1	11.991	0.039	109	(0.73, 1.32, 1.49, 0.00)

^a^Districts: number of districts

^b^LLR: log-likelihood ratio

^c^Obs: number of observations

^d^RR: relative risk

The Elbow method selected 4% as the optimal MRCS, while the MCS-P and MCHS-P selected 2% as optimal. These three methods identified clusters that either consisted of smaller clusters within the clusters detected by the default setting, smaller clusters partially overlapping with the default clusters, or smaller clusters in entirely new regions without any overlap with the default clusters. Those clusters could provide more informative and interpretable results compared to those identified using the default setting. However, the clusters obtained using these methods are primarily composed of very small clusters consisting of only one or two regions. Particularly when using the MCHS-P method, it might be difficult to consider them as clusters since some reported clusters consisting of one region are remote and not adjacent to other clusters.

## Discussion and conclusion

To select the optimal MRCS value when using the spatial scan statistics, several optimization criteria have been developed such as the Gini coefficient [17, 19–21], MCS-P [23], MCHS-P [24], and Elbow method [22]. However, the Gini coefficient for the multinomial model has not been developed. The other optimization criteria (i.e., MCS-P, MCHS-P and Elbow method) have been developed and evaluated only for the Poisson model. Thus, we have proposed the SCIC to choose the optimal MRCS value for the multinomial-based spatial scan statistic.

We have evaluated the performance of the proposed methods through an extensive simulation study. Particularly, in the scenarios with the two heterogeneous clusters, we observed consistent and robust results for both the multinomial and ordinal models: (1) the SCICs mostly selected the MRCS value that matched the size of the true cluster as the optimal MRCS, and (2) the detection accuracy achieved at the optimal MRCS using SCICs outperformed the results obtained with the default setting. We have also evaluated the performance of the existing methods by appropriately applying to the multinomial model. The overall detection accuracy obtained using the proposed methods was comparable to that of other existing methods. This might be because these methods are all defined based on the likelihood. While the sensitivity of the proposed methods at the selected optimal MRCS value was higher than the default setting, the overall sensitivity was slightly lower. This could be considered a limitation of our method, as it suggests the potential for missing certain regions of true clusters in some situations. However, this trend was observed across all evaluated methods.

Despite delivering comparable performance, the existing methods have certain limitations. The Gini coefficient cannot be applied to the multinomial model. The Elbow method assumes that the sum of the LRT statistic for significant clusters monotonically increases as the MRCS values increase. However, in certain cases, multiple significant clusters may be reported at small MRCS values, causing the sum of the LRT statistic to initially increase and then decrease. As a result, identifying the proper elbow point becomes challenging. The MCS-P and MCHS-P methods require distinct definitions of the union log-likelihood ratio test statistic for each probability model. Additionally, the MCHS-P method suffers from a lengthy computation time due to the necessity of calculating the spatial contiguity matrix.

We have introduced the SCICs for the multinomial model, which can be easily extended to all probability models based on likelihood. These criteria offer computational efficiency as they directly calculate the criteria without requiring any modification of the test statistics. Consequently, we propose that utilizing the SCICs when selecting the optimal MRCS for the multinomial- and ordinal-based spatial scan statistics would be beneficial. By employing the SCICs, we anticipate identifying more meaningful and interpretable clusters compared to using the default setting.

Between the two versions of the SCICs, we find that the SCIC_1_ appears more appropriate as it includes information of the number of cases in addition to the regional information. Through simulation results of the multinomial model, we observed that the SCIC_1_ outperformed the SCIC_2_ in terms of PPV. However, in the simulation results of the ordinal model, both the overall sensitivity and PPV were comparable between the SCIC_1_ and SCIC_2_ in the single cluster setting. In the two clusters setting, the overall sensitivity of SCIC_2_ was slightly higher than that of SCIC_1_. Nevertheless, the differences in overall sensitivity between the SCIC_1_ and SCIC_2_ were minimal and not deemed significant.

In summary, we propose a novel approach to optimizing the MRCS value for the multinomial-based spatial scan statistic. Compared to the default setting, our SCIC measures improve the accuracy of reported clusters. Also, the SCIC measures have the advantages of easily extending to other probability models over the existing measures. In public health and disease surveillance, our approach has the potential to enhance spatial cluster detection by providing greater accuracy and meaningful insights.

### Supplementary Information

** Additional file 1.** Simulation results for multinomial model (A1–A22).

** Additional file 2.** Simulation results for ordinal model (A23–A48).

## Data Availability

The datasets used and/or analyzed during the current study are available from the corresponding author on reasonable request.

## Abbreviations

BIC: Bayes information criterion
KCHS: Korea Community Health Survey
LRT: Likelihood ratio test
LLR: Log-likelihood ratio
MCS-P: Maximum clustering set-proportion
MRCS: Maximum reported cluster size
MSWS: Maximum scanning window size
PPV: Positive predicted value
SCIC: Spatial cluster information criterion
