# Supplementary material for: Optimizing the maximum reported cluster size for the multinomial-based spatial scan statistic
Source: Int J Health Geogr. 2023 Nov 8;22:30. doi: 10.1186/s12942-023-00353-4 (PMC10631089; doi:10.1186/s12942-023-00353-4)
Supplement: Supplementary file 1 — Additional file 1. Simulation results for multinomial model (A1–A22). [file 12942_2023_353_MOESM1_ESM.docx]

Table A1. Multinomial model: Simulation results for the true cluster model (A) and alternative hypothesis (1) using elliptical windows.

|  |  | Maximum reported cluster size (MRCS) | | | | | | | | | | | | | | | | | | Default  Setting |
| --- | --- | --- | --- | --- | --- | --- | --- | --- | --- | --- | --- | --- | --- | --- | --- | --- | --- | --- | --- | --- |
|  |  | 1% | 2% | 3% | 4% | 5% | 6% | 8% | 10% | 12% | 15% | 20% | 25% | 30% | 35% | 40% | 45% | 50% | Overall |  |
| SCIC_1_ | Freq^a^ | 0 | 0 | 2 | 10 | 47 | 33 | 543 | 180 | 63 | 35 | 25 | 2 | 7 | 3 | 2 | 1 | 1 | 954 | 954 |
|  | Sen^b^ | NA | NA | 0.400 | 0.480 | 0.562 | 0.685 | 0.940 | 0.969 | 0.971 | 0.994 | 0.976 | 1.000 | 0.971 | 1.000 | 1.000 | 1.000 | 1.000 | 0.918 | 0.945 |
|  | PPV^c^ | NA | NA | 1.000 | 0.940 | 0.933 | 0.968 | 0.976 | 0.791 | 0.637 | 0.536 | 0.371 | 0.279 | 0.224 | 0.176 | 0.156 | 0.167 | 0.135 | 0.871 | 0.826 |
|  | Mis^d^ | NA | NA | 0.043 | 0.042 | 0.035 | 0.025 | 0.007 | 0.022 | 0.049 | 0.069 | 0.130 | 0.188 | 0.248 | 0.377 | 0.391 | 0.362 | 0.464 | 0.025 | 0.042 |
| SCIC_2_ | Freq | 0 | 1 | 3 | 15 | 44 | 37 | 519 | 166 | 65 | 33 | 27 | 6 | 11 | 8 | 4 | 8 | 7 | 954 | 954 |
|  | Sen | NA | 0.200 | 0.467 | 0.573 | 0.600 | 0.686 | 0.941 | 0.967 | 0.972 | 0.994 | 0.978 | 0.967 | 0.982 | 1.000 | 1.000 | 0.975 | 0.943 | 0.919 | 0.945 |
|  | PPV | NA | 1.000 | 1.000 | 0.947 | 0.954 | 0.967 | 0.977 | 0.789 | 0.639 | 0.529 | 0.352 | 0.231 | 0.215 | 0.169 | 0.154 | 0.140 | 0.118 | 0.849 | 0.826 |
|  | Mis | NA | 0.058 | 0.039 | 0.035 | 0.032 | 0.025 | 0.006 | 0.023 | 0.048 | 0.071 | 0.141 | 0.237 | 0.271 | 0.366 | 0.399 | 0.438 | 0.522 | 0.037 | 0.042 |
| Elbow | Freq | 0 | 0 | 5 | 27 | 46 | 39 | 559 | 160 | 63 | 33 | 9 | 7 | 3 | 1 | 1 | 1 | 0 | 954 | 954 |
|  | Sen | NA | NA | 0.560 | 0.689 | 0.609 | 0.692 | 0.946 | 0.963 | 0.975 | 0.964 | 1.000 | 0.971 | 0.933 | 1.000 | 1.000 | 1.000 | NA | 0.916 | 0.945 |
|  | PPV | NA | NA | 0.933 | 0.963 | 0.954 | 0.947 | 0.972 | 0.784 | 0.641 | 0.476 | 0.300 | 0.231 | 0.169 | 0.116 | 0.147 | 0.167 | NA | 0.882 | 0.826 |
|  | Mis | NA | NA | 0.035 | 0.025 | 0.032 | 0.027 | 0.007 | 0.024 | 0.048 | 0.089 | 0.172 | 0.238 | 0.353 | 0.551 | 0.420 | 0.362 | NA | 0.024 | 0.042 |
| MCS  -P | Freq | 0 | 0 | 6 | 16 | 29 | 31 | 548 | 185 | 65 | 34 | 25 | 3 | 5 | 3 | 0 | 3 | 1 | 954 | 954 |
|  | Sen | NA | NA | 0.400 | 0.613 | 0.607 | 0.684 | 0.945 | 0.964 | 0.972 | 0.994 | 0.992 | 0.800 | 1.000 | 0.933 | NA | 1.000 | 1.000 | 0.926 | 0.945 |
|  | PPV | NA | NA | 0.833 | 0.988 | 0.932 | 0.946 | 0.985 | 0.797 | 0.677 | 0.566 | 0.414 | 0.247 | 0.256 | 0.222 | NA | 0.163 | 0.161 | 0.882 | 0.826 |
|  | Mis | NA | NA | 0.048 | 0.029 | 0.032 | 0.026 | 0.005 | 0.021 | 0.037 | 0.057 | 0.104 | 0.184 | 0.212 | 0.242 | NA | 0.372 | 0.377 | 0.021 | 0.042 |
| MCHS  -P | Freq | 0 | 0 | 2 | 14 | 24 | 29 | 542 | 188 | 70 | 39 | 28 | 4 | 6 | 3 | 1 | 3 | 1 | 954 | 954 |
|  | Sen | NA | NA | 0.700 | 0.614 | 0.633 | 0.710 | 0.943 | 0.962 | 0.974 | 0.995 | 0.993 | 0.950 | 0.900 | 0.933 | 0.800 | 1.000 | 1.000 | 0.932 | 0.945 |
|  | PPV | NA | NA | 1.000 | 0.943 | 0.939 | 0.957 | 0.977 | 0.786 | 0.656 | 0.540 | 0.401 | 0.249 | 0.223 | 0.222 | 0.129 | 0.163 | 0.161 | 0.865 | 0.826 |
|  | Mis | NA | NA | 0.022 | 0.032 | 0.031 | 0.024 | 0.006 | 0.023 | 0.043 | 0.066 | 0.111 | 0.217 | 0.258 | 0.242 | 0.406 | 0.372 | 0.377 | 0.025 | 0.042 |

^a^Freq: frequency. ^b^Sen: sensitivity. ^c^PPV: positive predictive value. ^d^Mis: misclassification.

Table A2. Multinomial model: Simulation results for the true cluster model (A) and alternative hypothesis (2) using elliptical windows.

|  |  | Maximum reported cluster size (MRCS) | | | | | | | | | | | | | | | | | | Default  Setting |
| --- | --- | --- | --- | --- | --- | --- | --- | --- | --- | --- | --- | --- | --- | --- | --- | --- | --- | --- | --- | --- |
|  |  | 1% | 2% | 3% | 4% | 5% | 6% | 8% | 10% | 12% | 15% | 20% | 25% | 30% | 35% | 40% | 45% | 50% | Overall |  |
| SCIC_1_ | Freq^a^ | 0 | 1 | 2 | 10 | 40 | 52 | 458 | 146 | 90 | 42 | 33 | 2 | 4 | 4 | 3 | 0 | 2 | 889 | 889 |
|  | Sen^b^ | NA | 0.000 | 0.200 | 0.360 | 0.575 | 0.658 | 0.924 | 0.962 | 0.971 | 0.981 | 0.970 | 1.000 | 1.000 | 0.450 | 1.000 | NA | 1.000 | 0.898 | 0.933 |
|  | PPV^c^ | NA | 0.000 | 0.500 | 0.850 | 0.942 | 0.933 | 0.967 | 0.785 | 0.650 | 0.541 | 0.403 | 0.236 | 0.255 | 0.098 | 0.192 | NA | 0.143 | 0.844 | 0.796 |
|  | Mis^d^ | NA | 0.087 | 0.072 | 0.054 | 0.034 | 0.029 | 0.008 | 0.023 | 0.044 | 0.065 | 0.112 | 0.254 | 0.217 | 0.337 | 0.304 | NA | 0.435 | 0.029 | 0.046 |
| SCIC_2_ | Freq | 0 | 0 | 2 | 10 | 40 | 57 | 438 | 142 | 88 | 38 | 37 | 5 | 9 | 8 | 7 | 4 | 4 | 889 | 889 |
|  | Sen | NA | NA | 0.200 | 0.440 | 0.580 | 0.660 | 0.928 | 0.962 | 0.970 | 0.979 | 0.962 | 0.960 | 0.978 | 0.800 | 1.000 | 1.000 | 1.000 | 0.902 | 0.933 |
|  | PPV | NA | NA | 0.500 | 0.950 | 0.931 | 0.934 | 0.974 | 0.782 | 0.642 | 0.536 | 0.372 | 0.222 | 0.211 | 0.157 | 0.163 | 0.125 | 0.138 | 0.827 | 0.796 |
|  | Mis | NA | NA | 0.072 | 0.043 | 0.035 | 0.029 | 0.007 | 0.024 | 0.046 | 0.066 | 0.134 | 0.255 | 0.282 | 0.322 | 0.400 | 0.525 | 0.453 | 0.039 | 0.046 |
| Elbow | Freq | 0 | 0 | 3 | 14 | 43 | 58 | 467 | 145 | 88 | 31 | 23 | 3 | 4 | 5 | 3 | 1 | 0 | 888 | 889 |
|  | Sen | NA | NA | 0.333 | 0.514 | 0.595 | 0.672 | 0.927 | 0.957 | 0.961 | 0.968 | 0.948 | 0.933 | 0.950 | 0.760 | 1.000 | 1.000 | NA | 0.896 | 0.933 |
|  | PPV | NA | NA | 0.667 | 0.879 | 0.931 | 0.926 | 0.966 | 0.773 | 0.633 | 0.520 | 0.329 | 0.203 | 0.182 | 0.152 | 0.161 | 0.094 | NA | 0.848 | 0.796 |
|  | Mis | NA | NA | 0.058 | 0.041 | 0.034 | 0.028 | 0.008 | 0.026 | 0.047 | 0.071 | 0.163 | 0.271 | 0.315 | 0.316 | 0.440 | 0.696 | NA | 0.031 | 0.046 |
| MCS  -P | Freq | 0 | 1 | 4 | 8 | 29 | 40 | 438 | 158 | 89 | 52 | 45 | 3 | 7 | 7 | 5 | 1 | 2 | 889 | 889 |
|  | Sen | NA | 0.000 | 0.000 | 0.350 | 0.586 | 0.610 | 0.933 | 0.965 | 0.980 | 0.985 | 0.978 | 1.000 | 0.943 | 0.686 | 1.000 | 1.000 | 1.000 | 0.911 | 0.933 |
|  | PPV | NA | 0.000 | 0.000 | 0.688 | 0.917 | 0.812 | 0.982 | 0.804 | 0.691 | 0.561 | 0.423 | 0.474 | 0.267 | 0.152 | 0.191 | 0.179 | 0.143 | 0.829 | 0.796 |
|  | Mis | NA | 0.087 | 0.101 | 0.062 | 0.035 | 0.040 | 0.006 | 0.020 | 0.034 | 0.058 | 0.101 | 0.121 | 0.193 | 0.300 | 0.307 | 0.333 | 0.435 | 0.030 | 0.046 |
| MCHS  -P | Freq | 0 | 1 | 1 | 6 | 26 | 37 | 436 | 162 | 95 | 54 | 47 | 2 | 7 | 7 | 5 | 1 | 2 | 889 | 889 |
|  | Sen | NA | 0.000 | 0.000 | 0.400 | 0.592 | 0.643 | 0.933 | 0.964 | 0.981 | 0.985 | 0.970 | 1.000 | 0.943 | 0.686 | 1.000 | 1.000 | 1.000 | 0.920 | 0.933 |
|  | PPV | NA | 0.000 | 0.000 | 0.750 | 0.915 | 0.850 | 0.976 | 0.788 | 0.672 | 0.557 | 0.415 | 0.294 | 0.267 | 0.152 | 0.191 | 0.179 | 0.143 | 0.823 | 0.796 |
|  | Mis | NA | 0.087 | 0.101 | 0.056 | 0.035 | 0.036 | 0.007 | 0.023 | 0.038 | 0.059 | 0.105 | 0.174 | 0.193 | 0.300 | 0.307 | 0.333 | 0.435 | 0.031 | 0.046 |

^a^Freq: frequency. ^b^Sen: sensitivity. ^c^PPV: positive predictive value. ^d^Mis: misclassification.

Table A3. Multinomial model: Simulation results for the true cluster model (A) and alternative hypothesis (3) using elliptical windows.

|  |  | Maximum reported cluster size (MRCS) | | | | | | | | | | | | | | | | | | Default  Setting |
| --- | --- | --- | --- | --- | --- | --- | --- | --- | --- | --- | --- | --- | --- | --- | --- | --- | --- | --- | --- | --- |
|  |  | 1% | 2% | 3% | 4% | 5% | 6% | 8% | 10% | 12% | 15% | 20% | 25% | 30% | 35% | 40% | 45% | 50% | Overall |  |
| SCIC_1_ | Freq^a^ | 0 | 1 | 4 | 4 | 50 | 53 | 435 | 165 | 78 | 46 | 25 | 10 | 2 | 1 | 3 | 0 | 2 | 879 | 879 |
|  | Sen^b^ | NA | 0.200 | 0.350 | 0.400 | 0.572 | 0.679 | 0.921 | 0.953 | 0.956 | 0.983 | 0.968 | 0.900 | 1.000 | 1.000 | 1.000 | NA | 1.000 | 0.895 | 0.928 |
|  | PPV^c^ | NA | 1.000 | 0.917 | 1.000 | 0.938 | 0.936 | 0.969 | 0.767 | 0.653 | 0.543 | 0.364 | 0.281 | 0.265 | 0.238 | 0.137 | NA | 0.152 | 0.845 | 0.802 |
|  | Mis^d^ | NA | 0.058 | 0.051 | 0.043 | 0.035 | 0.027 | 0.009 | 0.027 | 0.042 | 0.066 | 0.140 | 0.177 | 0.210 | 0.232 | 0.488 | NA | 0.406 | 0.030 | 0.043 |
| SCIC_2_ | Freq | 0 | 1 | 5 | 8 | 46 | 51 | 428 | 166 | 73 | 43 | 25 | 13 | 2 | 7 | 2 | 3 | 6 | 879 | 879 |
|  | Sen | NA | 0.200 | 0.400 | 0.650 | 0.578 | 0.686 | 0.921 | 0.949 | 0.953 | 0.981 | 0.992 | 0.908 | 1.000 | 1.000 | 1.000 | 0.933 | 0.933 | 0.897 | 0.928 |
|  | PPV | NA | 1.000 | 0.933 | 0.950 | 0.940 | 0.954 | 0.968 | 0.763 | 0.649 | 0.541 | 0.364 | 0.267 | 0.265 | 0.187 | 0.113 | 0.133 | 0.126 | 0.834 | 0.802 |
|  | Mis | NA | 0.058 | 0.046 | 0.029 | 0.034 | 0.026 | 0.009 | 0.028 | 0.043 | 0.066 | 0.141 | 0.193 | 0.210 | 0.333 | 0.572 | 0.444 | 0.481 | 0.036 | 0.043 |
| Elbow | Freq | 0 | 0 | 7 | 11 | 52 | 51 | 446 | 165 | 81 | 32 | 16 | 13 | 0 | 3 | 1 | 0 | 0 | 878 | 879 |
|  | Sen | NA | NA | 0.543 | 0.691 | 0.592 | 0.686 | 0.926 | 0.950 | 0.951 | 0.981 | 0.988 | 0.908 | NA | 1.000 | 1.000 | NA | NA | 0.896 | 0.928 |
|  | PPV | NA | NA | 0.952 | 0.945 | 0.929 | 0.917 | 0.966 | 0.767 | 0.626 | 0.522 | 0.342 | 0.243 | NA | 0.194 | 0.185 | NA | NA | 0.850 | 0.802 |
|  | Mis | NA | NA | 0.035 | 0.026 | 0.034 | 0.028 | 0.009 | 0.027 | 0.049 | 0.073 | 0.150 | 0.222 | NA | 0.343 | 0.319 | NA | NA | 0.028 | 0.043 |
| MCS  -P | Freq | 0 | 1 | 7 | 13 | 34 | 35 | 435 | 159 | 93 | 52 | 27 | 12 | 4 | 3 | 1 | 1 | 2 | 879 | 879 |
|  | Sen | NA | 0.000 | 0.343 | 0.523 | 0.535 | 0.640 | 0.925 | 0.965 | 0.963 | 0.985 | 0.978 | 0.917 | 1.000 | 1.000 | 1.000 | 1.000 | 1.000 | 0.904 | 0.928 |
|  | PPV | NA | 0.000 | 0.714 | 0.738 | 0.865 | 0.888 | 0.975 | 0.801 | 0.673 | 0.559 | 0.426 | 0.311 | 0.276 | 0.234 | 0.185 | 0.161 | 0.152 | 0.837 | 0.802 |
|  | Mis | NA | 0.087 | 0.056 | 0.047 | 0.040 | 0.033 | 0.008 | 0.020 | 0.037 | 0.059 | 0.098 | 0.155 | 0.192 | 0.237 | 0.319 | 0.377 | 0.406 | 0.027 | 0.043 |
| MCHS  -P | Freq | 0 | 0 | 2 | 10 | 29 | 32 | 435 | 164 | 94 | 54 | 32 | 15 | 5 | 3 | 1 | 1 | 2 | 879 | 879 |
|  | Sen | NA | NA | 0.600 | 0.720 | 0.586 | 0.681 | 0.926 | 0.963 | 0.964 | 0.985 | 0.950 | 0.920 | 1.000 | 1.000 | 1.000 | 1.000 | 1.000 | 0.919 | 0.928 |
|  | PPV | NA | NA | 1.000 | 0.940 | 0.928 | 0.940 | 0.968 | 0.785 | 0.664 | 0.552 | 0.393 | 0.295 | 0.249 | 0.234 | 0.185 | 0.161 | 0.152 | 0.830 | 0.802 |
|  | Mis | NA | NA | 0.029 | 0.025 | 0.034 | 0.027 | 0.008 | 0.023 | 0.040 | 0.061 | 0.116 | 0.171 | 0.241 | 0.237 | 0.319 | 0.377 | 0.406 | 0.030 | 0.043 |

^a^Freq: frequency. ^b^Sen: sensitivity. ^c^PPV: positive predictive value. ^d^Mis: misclassification.

Table A4. Multinomial model: Simulation results for the true cluster model (A) and alternative hypothesis (4) using elliptical windows.

|  |  | Maximum reported cluster size (MRCS) | | | | | | | | | | | | | | | | | | Default  Setting |
| --- | --- | --- | --- | --- | --- | --- | --- | --- | --- | --- | --- | --- | --- | --- | --- | --- | --- | --- | --- | --- |
|  |  | 1% | 2% | 3% | 4% | 5% | 6% | 8% | 10% | 12% | 15% | 20% | 25% | 30% | 35% | 40% | 45% | 50% | Overall |  |
| SCIC_1_ | Freq^a^ | 0 | 4 | 6 | 11 | 44 | 48 | 452 | 160 | 94 | 43 | 23 | 7 | 7 | 2 | 1 | 0 | 1 | 903 | 903 |
|  | Sen^b^ | NA | 0.200 | 0.300 | 0.400 | 0.536 | 0.654 | 0.916 | 0.948 | 0.962 | 0.958 | 0.922 | 1.000 | 0.857 | 0.800 | 1.000 | NA | 1.000 | 0.883 | 0.920 |
|  | PPV^c^ | NA | 1.000 | 0.750 | 1.000 | 0.866 | 0.953 | 0.966 | 0.773 | 0.655 | 0.526 | 0.375 | 0.256 | 0.224 | 0.159 | 0.192 | NA | 0.147 | 0.842 | 0.785 |
|  | Mis^d^ | NA | 0.058 | 0.058 | 0.043 | 0.040 | 0.028 | 0.009 | 0.026 | 0.041 | 0.070 | 0.124 | 0.219 | 0.224 | 0.341 | 0.304 | NA | 0.420 | 0.029 | 0.051 |
| SCIC_2_ | Freq | 0 | 4 | 7 | 12 | 42 | 42 | 431 | 157 | 91 | 41 | 29 | 12 | 12 | 8 | 3 | 6 | 6 | 903 | 903 |
|  | Sen | NA | 0.200 | 0.314 | 0.433 | 0.538 | 0.662 | 0.916 | 0.946 | 0.954 | 0.956 | 0.931 | 0.967 | 0.883 | 0.925 | 0.933 | 0.967 | 0.933 | 0.885 | 0.920 |
|  | PPV | NA | 1.000 | 0.786 | 0.983 | 0.877 | 0.976 | 0.965 | 0.770 | 0.652 | 0.520 | 0.345 | 0.242 | 0.192 | 0.151 | 0.164 | 0.137 | 0.123 | 0.814 | 0.785 |
|  | Mis | NA | 0.058 | 0.056 | 0.042 | 0.039 | 0.026 | 0.009 | 0.027 | 0.042 | 0.073 | 0.146 | 0.228 | 0.281 | 0.402 | 0.353 | 0.447 | 0.495 | 0.043 | 0.051 |
| Elbow | Freq | 0 | 3 | 8 | 13 | 52 | 50 | 458 | 148 | 96 | 35 | 21 | 10 | 6 | 2 | 1 | 0 | 0 | 903 | 903 |
|  | Sen | NA | 0.200 | 0.400 | 0.554 | 0.573 | 0.672 | 0.923 | 0.942 | 0.950 | 0.937 | 0.914 | 0.960 | 0.800 | 1.000 | 1.000 | NA | NA | 0.883 | 0.920 |
|  | PPV | NA | 1.000 | 0.875 | 0.985 | 0.873 | 0.948 | 0.967 | 0.764 | 0.639 | 0.504 | 0.302 | 0.246 | 0.138 | 0.119 | 0.192 | NA | NA | 0.842 | 0.785 |
|  | Mis | NA | 0.058 | 0.047 | 0.033 | 0.038 | 0.027 | 0.008 | 0.028 | 0.046 | 0.077 | 0.170 | 0.225 | 0.353 | 0.536 | 0.304 | NA | NA | 0.032 | 0.051 |
| MCS  -P | Freq | 0 | 3 | 7 | 3 | 30 | 41 | 445 | 158 | 106 | 49 | 37 | 7 | 8 | 1 | 2 | 3 | 3 | 903 | 903 |
|  | Sen | NA | 0.133 | 0.286 | 0.800 | 0.593 | 0.620 | 0.916 | 0.952 | 0.958 | 0.967 | 0.951 | 0.971 | 0.875 | 1.000 | 1.000 | 1.000 | 1.000 | 0.901 | 0.920 |
|  | PPV | NA | 0.667 | 0.714 | 0.933 | 0.878 | 0.902 | 0.970 | 0.795 | 0.676 | 0.545 | 0.422 | 0.322 | 0.223 | 0.217 | 0.189 | 0.183 | 0.147 | 0.830 | 0.785 |
|  | Mis | NA | 0.068 | 0.060 | 0.019 | 0.036 | 0.033 | 0.009 | 0.022 | 0.037 | 0.062 | 0.102 | 0.151 | 0.232 | 0.261 | 0.312 | 0.333 | 0.420 | 0.030 | 0.051 |
| MCHS  -P | Freq | 0 | 1 | 3 | 3 | 29 | 37 | 443 | 162 | 109 | 52 | 38 | 8 | 8 | 1 | 3 | 3 | 3 | 903 | 903 |
|  | Sen | NA | 0.200 | 0.267 | 0.800 | 0.593 | 0.654 | 0.916 | 0.951 | 0.960 | 0.965 | 0.953 | 0.975 | 0.875 | 1.000 | 0.933 | 1.000 | 1.000 | 0.908 | 0.920 |
|  | PPV | NA | 1.000 | 0.667 | 0.933 | 0.874 | 0.931 | 0.965 | 0.786 | 0.666 | 0.533 | 0.403 | 0.312 | 0.229 | 0.217 | 0.169 | 0.183 | 0.147 | 0.822 | 0.785 |
|  | Mis | NA | 0.058 | 0.063 | 0.019 | 0.036 | 0.030 | 0.009 | 0.024 | 0.039 | 0.066 | 0.110 | 0.161 | 0.221 | 0.261 | 0.343 | 0.333 | 0.420 | 0.032 | 0.051 |

^a^Freq: frequency. ^b^Sen: sensitivity. ^c^PPV: positive predictive value. ^d^Mis: misclassification.

Table A5. Multinomial model: Simulation results for the true cluster model (C) and alternative hypothesis (1) using elliptical windows.

|  |  | Maximum reported cluster size (MRCS) | | | | | | | | | | | | | | | | | | Default  Setting |
| --- | --- | --- | --- | --- | --- | --- | --- | --- | --- | --- | --- | --- | --- | --- | --- | --- | --- | --- | --- | --- |
|  |  | 1% | 2% | 3% | 4% | 5% | 6% | 8% | 10% | 12% | 15% | 20% | 25% | 30% | 35% | 40% | 45% | 50% | Overall |  |
| SCIC_1_ | Freq^a^ | 0 | 3 | 1 | 6 | 10 | 34 | 148 | 213 | 396 | 73 | 49 | 19 | 6 | 10 | 8 | 5 | 2 | 983 | 983 |
|  | Sen^b^ | NA | 0.100 | 0.200 | 0.300 | 0.330 | 0.426 | 0.576 | 0.677 | 0.738 | 0.714 | 0.739 | 0.779 | 0.850 | 0.830 | 0.988 | 0.720 | 0.900 | 0.683 | 0.715 |
|  | PPV^c^ | NA | 1.000 | 1.000 | 1.000 | 0.980 | 0.955 | 0.953 | 0.931 | 0.926 | 0.703 | 0.533 | 0.463 | 0.415 | 0.336 | 0.289 | 0.195 | 0.259 | 0.869 | 0.738 |
|  | Mis^d^ | NA | 0.130 | 0.116 | 0.101 | 0.099 | 0.086 | 0.067 | 0.056 | 0.050 | 0.095 | 0.147 | 0.175 | 0.215 | 0.317 | 0.395 | 0.496 | 0.406 | 0.076 | 0.128 |
| SCIC_2_ | Freq | 0 | 4 | 1 | 9 | 24 | 58 | 146 | 191 | 342 | 63 | 50 | 31 | 15 | 23 | 8 | 15 | 3 | 983 | 983 |
|  | Sen | NA | 0.100 | 0.200 | 0.344 | 0.496 | 0.571 | 0.615 | 0.691 | 0.743 | 0.725 | 0.724 | 0.739 | 0.787 | 0.791 | 0.925 | 0.767 | 0.833 | 0.692 | 0.715 |
|  | PPV | NA | 1.000 | 1.000 | 0.950 | 0.978 | 0.963 | 0.939 | 0.925 | 0.922 | 0.702 | 0.480 | 0.374 | 0.318 | 0.274 | 0.240 | 0.201 | 0.185 | 0.833 | 0.738 |
|  | Mis | NA | 0.130 | 0.116 | 0.098 | 0.075 | 0.065 | 0.063 | 0.055 | 0.050 | 0.096 | 0.173 | 0.230 | 0.290 | 0.370 | 0.457 | 0.484 | 0.565 | 0.093 | 0.128 |
| Elbow | Freq | 0 | 1 | 2 | 14 | 61 | 104 | 179 | 212 | 294 | 60 | 26 | 20 | 5 | 5 | 0 | 0 | 0 | 983 | 983 |
|  | Sen | NA | 0.100 | 0.300 | 0.379 | 0.579 | 0.614 | 0.617 | 0.683 | 0.737 | 0.703 | 0.692 | 0.680 | 0.700 | 0.920 | NA | NA | NA | 0.670 | 0.715 |
|  | PPV | NA | 1.000 | 1.000 | 0.968 | 0.970 | 0.945 | 0.928 | 0.916 | 0.907 | 0.634 | 0.413 | 0.325 | 0.239 | 0.248 | NA | NA | NA | 0.873 | 0.738 |
|  | Mis | NA | 0.130 | 0.101 | 0.092 | 0.064 | 0.062 | 0.065 | 0.058 | 0.054 | 0.117 | 0.211 | 0.264 | 0.365 | 0.446 | NA | NA | NA | 0.075 | 0.128 |
| MCS  -P | Freq | 0 | 4 | 4 | 17 | 45 | 97 | 145 | 180 | 340 | 59 | 31 | 23 | 17 | 11 | 4 | 2 | 4 | 983 | 983 |
|  | Sen | NA | 0.025 | 0.050 | 0.335 | 0.509 | 0.630 | 0.626 | 0.699 | 0.755 | 0.703 | 0.726 | 0.822 | 0.865 | 0.936 | 0.975 | 0.950 | 1.000 | 0.693 | 0.715 |
|  | PPV | NA | 0.250 | 0.250 | 0.871 | 0.984 | 0.960 | 0.946 | 0.943 | 0.945 | 0.759 | 0.625 | 0.534 | 0.462 | 0.444 | 0.380 | 0.311 | 0.299 | 0.890 | 0.738 |
|  | Mis | NA | 0.152 | 0.159 | 0.101 | 0.072 | 0.058 | 0.061 | 0.050 | 0.043 | 0.076 | 0.104 | 0.138 | 0.167 | 0.194 | 0.236 | 0.312 | 0.341 | 0.064 | 0.128 |
| MCHS  -P | Freq | 0 | 1 | 1 | 14 | 42 | 93 | 140 | 183 | 329 | 67 | 39 | 32 | 20 | 13 | 4 | 2 | 3 | 983 | 983 |
|  | Sen | NA | 0.100 | 0.200 | 0.400 | 0.543 | 0.617 | 0.644 | 0.711 | 0.745 | 0.691 | 0.723 | 0.772 | 0.845 | 0.900 | 0.975 | 0.650 | 1.000 | 0.699 | 0.715 |
|  | PPV | NA | 1.000 | 1.000 | 0.976 | 0.980 | 0.937 | 0.933 | 0.936 | 0.925 | 0.709 | 0.574 | 0.463 | 0.424 | 0.414 | 0.380 | 0.194 | 0.301 | 0.866 | 0.738 |
|  | Mis | NA | 0.130 | 0.116 | 0.089 | 0.068 | 0.062 | 0.060 | 0.051 | 0.048 | 0.093 | 0.127 | 0.180 | 0.206 | 0.217 | 0.236 | 0.500 | 0.338 | 0.072 | 0.128 |

^a^Freq: frequency. ^b^Sen: sensitivity. ^c^PPV: positive predictive value. ^d^Mis: misclassification.

Table A6. Multinomial model: Simulation results for the true cluster model (C) and alternative hypothesis (2) using elliptical windows.

|  |  | Maximum reported cluster size (MRCS) | | | | | | | | | | | | | | | | | | Default  Setting |
| --- | --- | --- | --- | --- | --- | --- | --- | --- | --- | --- | --- | --- | --- | --- | --- | --- | --- | --- | --- | --- |
|  |  | 1% | 2% | 3% | 4% | 5% | 6% | 8% | 10% | 12% | 15% | 20% | 25% | 30% | 35% | 40% | 45% | 50% | Overall |  |
| SCIC_1_ | Freq^a^ | 0 | 0 | 2 | 7 | 30 | 41 | 131 | 180 | 319 | 92 | 58 | 26 | 20 | 8 | 9 | 14 | 1 | 938 | 938 |
|  | Sen^b^ | NA | NA | 0.100 | 0.271 | 0.313 | 0.417 | 0.507 | 0.642 | 0.692 | 0.676 | 0.714 | 0.808 | 0.825 | 0.900 | 0.811 | 0.864 | 1.000 | 0.640 | 0.693 |
|  | PPV^c^ | NA | NA | 0.500 | 0.943 | 0.956 | 0.902 | 0.931 | 0.916 | 0.920 | 0.664 | 0.582 | 0.460 | 0.400 | 0.353 | 0.262 | 0.256 | 0.313 | 0.829 | 0.712 |
|  | Mis^d^ | NA | NA | 0.145 | 0.110 | 0.102 | 0.092 | 0.079 | 0.062 | 0.055 | 0.106 | 0.122 | 0.183 | 0.222 | 0.274 | 0.367 | 0.410 | 0.319 | 0.090 | 0.135 |
| SCIC_2_ | Freq | 0 | 0 | 1 | 7 | 31 | 52 | 131 | 171 | 289 | 82 | 59 | 34 | 28 | 17 | 14 | 20 | 2 | 938 | 938 |
|  | Sen | NA | NA | 0.200 | 0.271 | 0.352 | 0.465 | 0.523 | 0.640 | 0.701 | 0.674 | 0.710 | 0.765 | 0.814 | 0.800 | 0.736 | 0.825 | 0.750 | 0.646 | 0.693 |
|  | PPV | NA | NA | 1.000 | 0.943 | 0.960 | 0.908 | 0.922 | 0.911 | 0.921 | 0.651 | 0.552 | 0.402 | 0.389 | 0.282 | 0.220 | 0.233 | 0.224 | 0.800 | 0.712 |
|  | Mis | NA | NA | 0.116 | 0.110 | 0.096 | 0.085 | 0.078 | 0.063 | 0.054 | 0.110 | 0.137 | 0.219 | 0.227 | 0.344 | 0.428 | 0.440 | 0.428 | 0.104 | 0.135 |
| Elbow | Freq | 0 | 0 | 2 | 12 | 45 | 76 | 160 | 187 | 288 | 80 | 40 | 23 | 15 | 2 | 4 | 3 | 0 | 937 | 938 |
|  | Sen | NA | NA | 0.100 | 0.333 | 0.420 | 0.507 | 0.527 | 0.634 | 0.692 | 0.665 | 0.715 | 0.722 | 0.733 | 0.750 | 0.700 | 0.967 | NA | 0.620 | 0.693 |
|  | PPV | NA | NA | 0.500 | 0.967 | 0.948 | 0.906 | 0.906 | 0.902 | 0.911 | 0.627 | 0.536 | 0.364 | 0.324 | 0.292 | 0.257 | 0.333 | NA | 0.840 | 0.712 |
|  | Mis | NA | NA | 0.145 | 0.099 | 0.088 | 0.080 | 0.079 | 0.066 | 0.057 | 0.120 | 0.147 | 0.246 | 0.288 | 0.319 | 0.330 | 0.285 | NA | 0.087 | 0.135 |
| MCS  -P | Freq | 0 | 1 | 6 | 13 | 42 | 69 | 113 | 169 | 292 | 75 | 62 | 31 | 25 | 9 | 15 | 12 | 4 | 938 | 938 |
|  | Sen | NA | 0.000 | 0.067 | 0.238 | 0.374 | 0.512 | 0.536 | 0.649 | 0.695 | 0.661 | 0.715 | 0.787 | 0.840 | 0.922 | 0.907 | 0.958 | 0.950 | 0.644 | 0.693 |
|  | PPV | NA | 0.000 | 0.250 | 0.769 | 0.907 | 0.916 | 0.940 | 0.920 | 0.927 | 0.713 | 0.605 | 0.516 | 0.458 | 0.401 | 0.343 | 0.332 | 0.288 | 0.829 | 0.712 |
|  | Mis | NA | 0.159 | 0.159 | 0.118 | 0.096 | 0.078 | 0.073 | 0.060 | 0.053 | 0.090 | 0.110 | 0.139 | 0.168 | 0.213 | 0.265 | 0.286 | 0.348 | 0.084 | 0.135 |
| MCHS  -P | Freq | 0 | 0 | 1 | 9 | 33 | 63 | 121 | 166 | 288 | 80 | 65 | 32 | 35 | 10 | 16 | 14 | 5 | 938 | 938 |
|  | Sen | NA | NA | 0.200 | 0.322 | 0.430 | 0.510 | 0.550 | 0.644 | 0.692 | 0.664 | 0.717 | 0.781 | 0.811 | 0.870 | 0.894 | 0.886 | 0.860 | 0.655 | 0.693 |
|  | PPV | NA | NA | 1.000 | 0.956 | 0.954 | 0.891 | 0.923 | 0.902 | 0.910 | 0.686 | 0.591 | 0.481 | 0.404 | 0.376 | 0.335 | 0.303 | 0.257 | 0.808 | 0.712 |
|  | Mis | NA | NA | 0.116 | 0.101 | 0.087 | 0.081 | 0.074 | 0.064 | 0.057 | 0.100 | 0.118 | 0.166 | 0.217 | 0.233 | 0.274 | 0.318 | 0.386 | 0.092 | 0.135 |

^a^Freq: frequency. ^b^Sen: sensitivity. ^c^PPV: positive predictive value. ^d^Mis: misclassification.

Table A7. Multinomial model: Simulation results for the true cluster model (C) and alternative hypothesis (3) using elliptical windows.

|  |  | Maximum reported cluster size (MRCS) | | | | | | | | | | | | | | | | | | Default  Setting |
| --- | --- | --- | --- | --- | --- | --- | --- | --- | --- | --- | --- | --- | --- | --- | --- | --- | --- | --- | --- | --- |
|  |  | 1% | 2% | 3% | 4% | 5% | 6% | 8% | 10% | 12% | 15% | 20% | 25% | 30% | 35% | 40% | 45% | 50% | Overall |  |
| SCIC_1_ | Freq^a^ | 0 | 0 | 3 | 15 | 36 | 37 | 133 | 181 | 322 | 85 | 65 | 33 | 10 | 9 | 13 | 7 | 3 | 952 | 952 |
|  | Sen^b^ | NA | NA | 0.100 | 0.247 | 0.325 | 0.403 | 0.506 | 0.625 | 0.714 | 0.661 | 0.720 | 0.742 | 0.830 | 0.844 | 0.885 | 0.786 | 1.000 | 0.635 | 0.696 |
|  | PPV^c^ | NA | NA | 0.667 | 0.933 | 0.951 | 0.946 | 0.934 | 0.927 | 0.922 | 0.666 | 0.546 | 0.437 | 0.359 | 0.344 | 0.299 | 0.198 | 0.323 | 0.834 | 0.699 |
|  | Mis^d^ | NA | NA | 0.140 | 0.112 | 0.100 | 0.092 | 0.078 | 0.063 | 0.052 | 0.105 | 0.142 | 0.194 | 0.270 | 0.275 | 0.350 | 0.532 | 0.304 | 0.091 | 0.139 |
| SCIC_2_ | Freq | 0 | 0 | 3 | 19 | 45 | 47 | 122 | 178 | 302 | 71 | 63 | 43 | 16 | 12 | 16 | 11 | 4 | 952 | 952 |
|  | Sen | NA | NA | 0.100 | 0.268 | 0.369 | 0.462 | 0.511 | 0.631 | 0.718 | 0.669 | 0.722 | 0.719 | 0.763 | 0.792 | 0.825 | 0.773 | 0.975 | 0.637 | 0.696 |
|  | PPV | NA | NA | 0.667 | 0.937 | 0.936 | 0.951 | 0.929 | 0.913 | 0.916 | 0.646 | 0.509 | 0.392 | 0.292 | 0.304 | 0.258 | 0.182 | 0.298 | 0.808 | 0.699 |
|  | Mis | NA | NA | 0.140 | 0.109 | 0.095 | 0.083 | 0.078 | 0.065 | 0.053 | 0.112 | 0.159 | 0.222 | 0.322 | 0.307 | 0.399 | 0.563 | 0.344 | 0.103 | 0.139 |
| Elbow | Freq | 0 | 0 | 2 | 21 | 61 | 73 | 157 | 197 | 285 | 65 | 50 | 28 | 9 | 1 | 2 | 0 | 0 | 951 | 952 |
|  | Sen | NA | NA | 0.100 | 0.290 | 0.438 | 0.503 | 0.512 | 0.631 | 0.704 | 0.657 | 0.712 | 0.743 | 0.678 | 0.700 | 0.950 | NA | NA | 0.613 | 0.696 |
|  | PPV | NA | NA | 0.500 | 0.933 | 0.948 | 0.936 | 0.910 | 0.907 | 0.894 | 0.617 | 0.442 | 0.362 | 0.210 | 0.333 | 0.365 | NA | NA | 0.840 | 0.699 |
|  | Mis | NA | NA | 0.145 | 0.106 | 0.085 | 0.078 | 0.080 | 0.066 | 0.060 | 0.120 | 0.192 | 0.251 | 0.417 | 0.246 | 0.246 | NA | NA | 0.089 | 0.139 |
| MCS  -P | Freq | 0 | 0 | 4 | 13 | 41 | 69 | 105 | 167 | 309 | 80 | 66 | 34 | 19 | 16 | 13 | 8 | 8 | 952 | 952 |
|  | Sen | NA | NA | 0.050 | 0.277 | 0.446 | 0.488 | 0.517 | 0.637 | 0.721 | 0.644 | 0.732 | 0.785 | 0.863 | 0.925 | 0.977 | 0.950 | 0.988 | 0.657 | 0.696 |
|  | PPV | NA | NA | 0.250 | 0.831 | 0.962 | 0.936 | 0.925 | 0.916 | 0.932 | 0.697 | 0.616 | 0.518 | 0.439 | 0.420 | 0.373 | 0.326 | 0.312 | 0.833 | 0.699 |
|  | Mis | NA | NA | 0.159 | 0.113 | 0.082 | 0.079 | 0.078 | 0.062 | 0.049 | 0.094 | 0.106 | 0.138 | 0.185 | 0.197 | 0.243 | 0.293 | 0.319 | 0.083 | 0.139 |
| MCHS  -P | Freq | 0 | 0 | 2 | 12 | 38 | 67 | 102 | 155 | 306 | 83 | 76 | 42 | 25 | 14 | 13 | 9 | 8 | 952 | 952 |
|  | Sen | NA | NA | 0.100 | 0.292 | 0.455 | 0.503 | 0.539 | 0.630 | 0.714 | 0.655 | 0.737 | 0.740 | 0.800 | 0.929 | 0.977 | 0.889 | 0.988 | 0.661 | 0.696 |
|  | PPV | NA | NA | 0.500 | 0.900 | 0.967 | 0.934 | 0.921 | 0.899 | 0.915 | 0.666 | 0.578 | 0.455 | 0.383 | 0.417 | 0.373 | 0.299 | 0.312 | 0.807 | 0.699 |
|  | Mis | NA | NA | 0.145 | 0.107 | 0.082 | 0.077 | 0.076 | 0.066 | 0.054 | 0.104 | 0.126 | 0.183 | 0.234 | 0.199 | 0.243 | 0.337 | 0.319 | 0.092 | 0.139 |

^a^Freq: frequency. ^b^Sen: sensitivity. ^c^PPV: positive predictive value. ^d^Mis: misclassification.

Table A8. Multinomial model: Simulation results for the true cluster model (C) and alternative hypothesis (4) using elliptical windows.

|  |  | Maximum reported cluster size (MRCS) | | | | | | | | | | | | | | | | | | Default  Setting |
| --- | --- | --- | --- | --- | --- | --- | --- | --- | --- | --- | --- | --- | --- | --- | --- | --- | --- | --- | --- | --- |
|  |  | 1% | 2% | 3% | 4% | 5% | 6% | 8% | 10% | 12% | 15% | 20% | 25% | 30% | 35% | 40% | 45% | 50% | Overall |  |
| SCIC_1_ | Freq^a^ | 0 | 3 | 6 | 9 | 30 | 34 | 156 | 170 | 298 | 79 | 62 | 38 | 20 | 13 | 17 | 9 | 5 | 949 | 949 |
|  | Sen^b^ | NA | 0.067 | 0.217 | 0.267 | 0.343 | 0.371 | 0.510 | 0.638 | 0.704 | 0.662 | 0.703 | 0.803 | 0.835 | 0.808 | 0.894 | 0.889 | 0.980 | 0.639 | 0.707 |
|  | PPV^c^ | NA | 0.667 | 0.917 | 1.000 | 0.958 | 0.911 | 0.940 | 0.931 | 0.913 | 0.668 | 0.542 | 0.460 | 0.406 | 0.302 | 0.294 | 0.247 | 0.237 | 0.819 | 0.649 |
|  | Mis^d^ | NA | 0.140 | 0.118 | 0.106 | 0.099 | 0.097 | 0.077 | 0.060 | 0.055 | 0.103 | 0.141 | 0.181 | 0.220 | 0.314 | 0.357 | 0.436 | 0.499 | 0.097 | 0.164 |
| SCIC_2_ | Freq | 0 | 2 | 6 | 10 | 35 | 49 | 149 | 147 | 246 | 74 | 66 | 44 | 38 | 27 | 23 | 24 | 9 | 949 | 949 |
|  | Sen | NA | 0.100 | 0.217 | 0.290 | 0.391 | 0.459 | 0.521 | 0.641 | 0.708 | 0.676 | 0.692 | 0.786 | 0.768 | 0.763 | 0.874 | 0.808 | 0.944 | 0.648 | 0.707 |
|  | PPV | NA | 1.000 | 0.917 | 1.000 | 0.957 | 0.928 | 0.933 | 0.927 | 0.906 | 0.645 | 0.504 | 0.425 | 0.321 | 0.261 | 0.270 | 0.210 | 0.221 | 0.766 | 0.649 |
|  | Mis | NA | 0.130 | 0.118 | 0.103 | 0.092 | 0.084 | 0.076 | 0.060 | 0.057 | 0.111 | 0.159 | 0.205 | 0.296 | 0.360 | 0.386 | 0.485 | 0.514 | 0.124 | 0.164 |
| Elbow | Freq | 0 | 2 | 6 | 17 | 57 | 76 | 186 | 178 | 240 | 81 | 52 | 30 | 18 | 2 | 3 | 0 | 0 | 948 | 949 |
|  | Sen | NA | 0.100 | 0.183 | 0.376 | 0.430 | 0.492 | 0.530 | 0.638 | 0.700 | 0.657 | 0.694 | 0.743 | 0.772 | 0.900 | 0.900 | NA | NA | 0.612 | 0.707 |
|  | PPV | NA | 1.000 | 1.000 | 0.957 | 0.950 | 0.917 | 0.921 | 0.913 | 0.888 | 0.609 | 0.469 | 0.355 | 0.295 | 0.410 | 0.277 | NA | NA | 0.829 | 0.649 |
|  | Mis | NA | 0.130 | 0.118 | 0.093 | 0.087 | 0.081 | 0.077 | 0.063 | 0.062 | 0.124 | 0.177 | 0.256 | 0.333 | 0.203 | 0.386 | NA | NA | 0.093 | 0.164 |
| MCS  -P | Freq | 0 | 2 | 4 | 19 | 47 | 59 | 150 | 148 | 245 | 79 | 67 | 45 | 32 | 12 | 22 | 10 | 8 | 949 | 949 |
|  | Sen | NA | 0.100 | 0.100 | 0.289 | 0.423 | 0.531 | 0.559 | 0.647 | 0.714 | 0.652 | 0.716 | 0.784 | 0.863 | 0.917 | 0.968 | 0.950 | 0.975 | 0.657 | 0.707 |
|  | PPV | NA | 1.000 | 0.500 | 0.753 | 0.945 | 0.944 | 0.953 | 0.933 | 0.934 | 0.709 | 0.605 | 0.522 | 0.462 | 0.404 | 0.376 | 0.327 | 0.294 | 0.824 | 0.649 |
|  | Mis | NA | 0.130 | 0.145 | 0.114 | 0.087 | 0.073 | 0.069 | 0.058 | 0.050 | 0.090 | 0.110 | 0.136 | 0.166 | 0.209 | 0.238 | 0.291 | 0.344 | 0.086 | 0.164 |
| MCHS  -P | Freq | 0 | 1 | 2 | 12 | 40 | 53 | 139 | 150 | 238 | 83 | 77 | 54 | 41 | 18 | 23 | 11 | 7 | 949 | 949 |
|  | Sen | NA | 0.100 | 0.300 | 0.367 | 0.450 | 0.528 | 0.564 | 0.639 | 0.706 | 0.648 | 0.705 | 0.776 | 0.805 | 0.783 | 0.939 | 0.955 | 0.971 | 0.663 | 0.707 |
|  | PPV | NA | 1.000 | 1.000 | 0.850 | 0.967 | 0.926 | 0.935 | 0.911 | 0.911 | 0.667 | 0.565 | 0.477 | 0.403 | 0.331 | 0.360 | 0.325 | 0.294 | 0.785 | 0.649 |
|  | Mis | NA | 0.130 | 0.101 | 0.100 | 0.083 | 0.075 | 0.071 | 0.062 | 0.056 | 0.104 | 0.130 | 0.169 | 0.216 | 0.269 | 0.255 | 0.295 | 0.344 | 0.099 | 0.164 |

^a^Freq: frequency. ^b^Sen: sensitivity. ^c^PPV: positive predictive value. ^d^Mis: misclassification.

Table A9. Multinomial model: Simulation results for the true cluster model (D) and alternative hypothesis (1) using elliptical windows.

|  |  | Maximum reported cluster size (MRCS) | | | | | | | | | | | | | | | | | | Default  Setting |
| --- | --- | --- | --- | --- | --- | --- | --- | --- | --- | --- | --- | --- | --- | --- | --- | --- | --- | --- | --- | --- |
|  |  | 1% | 2% | 3% | 4% | 5% | 6% | 8% | 10% | 12% | 15% | 20% | 25% | 30% | 35% | 40% | 45% | 50% | Overall |  |
| SCIC_1_ | Freq^a^ | 0 | 0 | 0 | 3 | 15 | 26 | 397 | 143 | 74 | 38 | 47 | 108 | 43 | 24 | 14 | 25 | 31 | 988 | 988 |
|  | Sen^b^ | NA | NA | NA | 0.200 | 0.400 | 0.469 | 0.789 | 0.858 | 0.868 | 0.766 | 0.813 | 0.963 | 0.965 | 0.929 | 0.886 | 0.980 | 0.903 | 0.829 | 0.835 |
|  | PPV^c^ | NA | NA | NA | 1.000 | 0.949 | 0.944 | 0.978 | 0.852 | 0.756 | 0.614 | 0.589 | 0.582 | 0.462 | 0.333 | 0.235 | 0.235 | 0.182 | 0.774 | 0.568 |
|  | Mis^d^ | NA | NA | NA | 0.116 | 0.091 | 0.081 | 0.034 | 0.043 | 0.061 | 0.114 | 0.125 | 0.125 | 0.195 | 0.323 | 0.438 | 0.503 | 0.599 | 0.106 | 0.241 |
| SCIC_2_ | Freq | 0 | 0 | 0 | 1 | 13 | 26 | 384 | 147 | 76 | 45 | 36 | 61 | 36 | 32 | 28 | 40 | 63 | 988 | 988 |
|  | Sen | NA | NA | NA | 0.200 | 0.431 | 0.477 | 0.801 | 0.900 | 0.878 | 0.840 | 0.850 | 0.944 | 0.883 | 0.859 | 0.821 | 0.875 | 0.892 | 0.834 | 0.835 |
|  | PPV | NA | NA | NA | 1.000 | 0.920 | 0.949 | 0.973 | 0.843 | 0.749 | 0.558 | 0.535 | 0.454 | 0.331 | 0.270 | 0.221 | 0.211 | 0.190 | 0.720 | 0.568 |
|  | Mis | NA | NA | NA | 0.116 | 0.090 | 0.080 | 0.033 | 0.042 | 0.062 | 0.129 | 0.152 | 0.204 | 0.296 | 0.377 | 0.442 | 0.502 | 0.563 | 0.143 | 0.241 |
| Elbow | Freq | 0 | 0 | 0 | 3 | 27 | 33 | 598 | 160 | 66 | 37 | 35 | 16 | 10 | 2 | 1 | 0 | 0 | 988 | 988 |
|  | Sen | NA | NA | NA | 0.467 | 0.441 | 0.494 | 0.826 | 0.873 | 0.885 | 0.827 | 0.846 | 0.894 | 0.830 | 0.750 | 1.000 | NA | NA | 0.816 | 0.835 |
|  | PPV | NA | NA | NA | 1.000 | 0.951 | 0.944 | 0.966 | 0.827 | 0.694 | 0.517 | 0.443 | 0.378 | 0.293 | 0.187 | 0.208 | NA | NA | 0.870 | 0.568 |
|  | Mis | NA | NA | NA | 0.077 | 0.086 | 0.079 | 0.031 | 0.048 | 0.078 | 0.147 | 0.195 | 0.250 | 0.336 | 0.493 | 0.551 | NA | NA | 0.058 | 0.241 |
| MCS  -P | Freq | 0 | 0 | 3 | 6 | 20 | 25 | 496 | 208 | 116 | 40 | 25 | 16 | 17 | 14 | 1 | 0 | 1 | 988 | 988 |
|  | Sen | NA | NA | 0.067 | 0.383 | 0.380 | 0.496 | 0.816 | 0.890 | 0.902 | 0.898 | 0.892 | 0.938 | 0.965 | 0.929 | 1.000 | NA | 1.000 | 0.831 | 0.835 |
|  | PPV | NA | NA | 0.333 | 1.000 | 0.883 | 0.932 | 0.982 | 0.880 | 0.775 | 0.683 | 0.716 | 0.682 | 0.560 | 0.431 | 0.435 | NA | 0.294 | 0.891 | 0.568 |
|  | Mis | NA | NA | 0.155 | 0.089 | 0.096 | 0.079 | 0.029 | 0.033 | 0.052 | 0.075 | 0.071 | 0.080 | 0.123 | 0.194 | 0.188 | NA | 0.348 | 0.044 | 0.241 |
| MCHS  -P | Freq | 0 | 0 | 0 | 2 | 11 | 15 | 440 | 215 | 124 | 53 | 51 | 27 | 26 | 15 | 6 | 2 | 1 | 988 | 988 |
|  | Sen | NA | NA | NA | 0.450 | 0.473 | 0.453 | 0.808 | 0.899 | 0.902 | 0.885 | 0.851 | 0.948 | 0.885 | 0.893 | 0.567 | 0.550 | 1.000 | 0.841 | 0.835 |
|  | PPV | NA | NA | NA | 1.000 | 0.894 | 0.887 | 0.978 | 0.859 | 0.767 | 0.622 | 0.568 | 0.512 | 0.418 | 0.452 | 0.198 | 0.298 | 0.294 | 0.841 | 0.568 |
|  | Mis | NA | NA | NA | 0.080 | 0.087 | 0.092 | 0.031 | 0.037 | 0.055 | 0.101 | 0.131 | 0.166 | 0.217 | 0.198 | 0.420 | 0.326 | 0.348 | 0.060 | 0.241 |

^a^Freq: frequency. ^b^Sen: sensitivity. ^c^PPV: positive predictive value. ^d^Mis: misclassification.

Table A10. Multinomial model: Simulation results for the true cluster model (D) and alternative hypothesis (2) using elliptical windows.

|  |  | Maximum reported cluster size (MRCS) | | | | | | | | | | | | | | | | | | Default  Setting |
| --- | --- | --- | --- | --- | --- | --- | --- | --- | --- | --- | --- | --- | --- | --- | --- | --- | --- | --- | --- | --- |
|  |  | 1% | 2% | 3% | 4% | 5% | 6% | 8% | 10% | 12% | 15% | 20% | 25% | 30% | 35% | 40% | 45% | 50% | Overall |  |
| SCIC_1_ | Freq^a^ | 0 | 0 | 3 | 8 | 25 | 36 | 371 | 115 | 76 | 46 | 44 | 114 | 59 | 24 | 8 | 9 | 18 | 956 | 956 |
|  | Sen^b^ | NA | NA | 0.200 | 0.175 | 0.324 | 0.414 | 0.702 | 0.782 | 0.776 | 0.728 | 0.805 | 0.928 | 0.959 | 0.921 | 0.838 | 0.944 | 0.950 | 0.753 | 0.778 |
|  | PPV^c^ | NA | NA | 1.000 | 0.875 | 0.920 | 0.980 | 0.969 | 0.827 | 0.704 | 0.623 | 0.598 | 0.595 | 0.480 | 0.358 | 0.260 | 0.193 | 0.195 | 0.778 | 0.616 |
|  | Mis^d^ | NA | NA | 0.116 | 0.125 | 0.103 | 0.087 | 0.047 | 0.056 | 0.080 | 0.108 | 0.119 | 0.112 | 0.177 | 0.272 | 0.388 | 0.576 | 0.581 | 0.100 | 0.202 |
| SCIC_2_ | Freq | 0 | 0 | 4 | 7 | 23 | 37 | 341 | 122 | 86 | 46 | 34 | 75 | 52 | 29 | 33 | 22 | 45 | 956 | 956 |
|  | Sen | NA | NA | 0.175 | 0.171 | 0.357 | 0.430 | 0.728 | 0.785 | 0.814 | 0.785 | 0.788 | 0.904 | 0.929 | 0.852 | 0.824 | 0.805 | 0.836 | 0.760 | 0.778 |
|  | PPV | NA | NA | 1.000 | 0.857 | 0.913 | 0.973 | 0.962 | 0.825 | 0.710 | 0.590 | 0.519 | 0.544 | 0.407 | 0.284 | 0.217 | 0.179 | 0.180 | 0.723 | 0.616 |
|  | Mis | NA | NA | 0.120 | 0.126 | 0.098 | 0.085 | 0.044 | 0.056 | 0.075 | 0.120 | 0.158 | 0.141 | 0.235 | 0.350 | 0.454 | 0.551 | 0.569 | 0.138 | 0.202 |
| Elbow | Freq | 0 | 0 | 2 | 8 | 30 | 40 | 470 | 146 | 86 | 45 | 46 | 46 | 20 | 11 | 3 | 2 | 0 | 955 | 956 |
|  | Sen | NA | NA | 0.200 | 0.175 | 0.350 | 0.420 | 0.731 | 0.771 | 0.806 | 0.760 | 0.796 | 0.889 | 0.900 | 0.718 | 0.933 | 0.500 | NA | 0.729 | 0.778 |
|  | PPV | NA | NA | 1.000 | 0.875 | 0.924 | 0.968 | 0.962 | 0.809 | 0.679 | 0.553 | 0.505 | 0.510 | 0.357 | 0.254 | 0.201 | 0.238 | NA | 0.824 | 0.616 |
|  | Mis | NA | NA | 0.116 | 0.125 | 0.099 | 0.087 | 0.044 | 0.061 | 0.086 | 0.136 | 0.163 | 0.165 | 0.284 | 0.357 | 0.546 | 0.290 | NA | 0.081 | 0.202 |
| MCS  -P | Freq | 0 | 1 | 5 | 9 | 16 | 29 | 433 | 152 | 105 | 50 | 34 | 58 | 38 | 15 | 6 | 4 | 1 | 956 | 956 |
|  | Sen | NA | 0.000 | 0.040 | 0.178 | 0.275 | 0.397 | 0.715 | 0.820 | 0.839 | 0.758 | 0.747 | 0.916 | 0.947 | 0.953 | 0.883 | 0.925 | 1.000 | 0.750 | 0.778 |
|  | PPV | NA | 0.000 | 0.200 | 0.778 | 0.729 | 0.947 | 0.977 | 0.848 | 0.747 | 0.634 | 0.584 | 0.614 | 0.524 | 0.436 | 0.372 | 0.325 | 0.303 | 0.832 | 0.616 |
|  | Mis | NA | 0.159 | 0.162 | 0.127 | 0.118 | 0.091 | 0.044 | 0.047 | 0.064 | 0.097 | 0.114 | 0.098 | 0.136 | 0.186 | 0.237 | 0.290 | 0.333 | 0.068 | 0.202 |
| MCHS  -P | Freq | 0 | 0 | 1 | 5 | 11 | 26 | 376 | 155 | 120 | 61 | 51 | 66 | 44 | 20 | 11 | 5 | 4 | 956 | 956 |
|  | Sen | NA | NA | 0.200 | 0.160 | 0.300 | 0.400 | 0.711 | 0.819 | 0.853 | 0.774 | 0.763 | 0.898 | 0.909 | 0.910 | 0.745 | 0.840 | 0.625 | 0.763 | 0.778 |
|  | PPV | NA | NA | 1.000 | 0.800 | 0.779 | 0.895 | 0.973 | 0.842 | 0.735 | 0.617 | 0.532 | 0.569 | 0.464 | 0.386 | 0.262 | 0.289 | 0.174 | 0.792 | 0.616 |
|  | Mis | NA | NA | 0.116 | 0.130 | 0.115 | 0.095 | 0.045 | 0.049 | 0.067 | 0.104 | 0.141 | 0.125 | 0.177 | 0.233 | 0.353 | 0.333 | 0.500 | 0.083 | 0.202 |

^a^Freq: frequency. ^b^Sen: sensitivity. ^c^PPV: positive predictive value. ^d^Mis: misclassification.

Table A11. Multinomial model: Simulation results for the true cluster model (D) and alternative hypothesis (3) using elliptical windows.

|  |  | Maximum reported cluster size (MRCS) | | | | | | | | | | | | | | | | | | Default  Setting |
| --- | --- | --- | --- | --- | --- | --- | --- | --- | --- | --- | --- | --- | --- | --- | --- | --- | --- | --- | --- | --- |
|  |  | 1% | 2% | 3% | 4% | 5% | 6% | 8% | 10% | 12% | 15% | 20% | 25% | 30% | 35% | 40% | 45% | 50% | Overall |  |
| SCIC_1_ | Freq^a^ | 0 | 1 | 6 | 4 | 22 | 41 | 333 | 96 | 72 | 45 | 63 | 140 | 62 | 20 | 18 | 15 | 21 | 959 | 959 |
|  | Sen^b^ | NA | 0.100 | 0.183 | 0.200 | 0.323 | 0.439 | 0.671 | 0.725 | 0.800 | 0.709 | 0.794 | 0.946 | 0.913 | 0.925 | 0.883 | 0.947 | 0.852 | 0.746 | 0.773 |
|  | PPV^c^ | NA | 1.000 | 1.000 | 1.000 | 0.906 | 0.970 | 0.972 | 0.810 | 0.730 | 0.622 | 0.597 | 0.579 | 0.447 | 0.338 | 0.259 | 0.213 | 0.175 | 0.748 | 0.572 |
|  | Mis^d^ | NA | 0.130 | 0.118 | 0.116 | 0.106 | 0.083 | 0.051 | 0.064 | 0.072 | 0.113 | 0.118 | 0.125 | 0.197 | 0.314 | 0.409 | 0.523 | 0.603 | 0.117 | 0.228 |
| SCIC_2_ | Freq | 0 | 1 | 5 | 4 | 21 | 37 | 335 | 105 | 86 | 48 | 60 | 68 | 57 | 21 | 35 | 28 | 48 | 959 | 959 |
|  | Sen | NA | 0.100 | 0.180 | 0.250 | 0.343 | 0.462 | 0.703 | 0.779 | 0.844 | 0.777 | 0.797 | 0.882 | 0.877 | 0.852 | 0.823 | 0.846 | 0.810 | 0.751 | 0.773 |
|  | PPV | NA | 1.000 | 1.000 | 1.000 | 0.917 | 0.969 | 0.967 | 0.809 | 0.726 | 0.581 | 0.518 | 0.474 | 0.372 | 0.265 | 0.223 | 0.201 | 0.171 | 0.705 | 0.572 |
|  | Mis | NA | 0.130 | 0.119 | 0.109 | 0.102 | 0.080 | 0.047 | 0.060 | 0.071 | 0.124 | 0.156 | 0.184 | 0.257 | 0.384 | 0.448 | 0.507 | 0.589 | 0.149 | 0.228 |
| Elbow | Freq | 0 | 1 | 6 | 9 | 29 | 41 | 488 | 131 | 88 | 47 | 48 | 41 | 17 | 5 | 6 | 1 | 0 | 958 | 959 |
|  | Sen | NA | 0.100 | 0.183 | 0.311 | 0.341 | 0.444 | 0.729 | 0.767 | 0.810 | 0.760 | 0.777 | 0.793 | 0.676 | 0.700 | 1.000 | 1.000 | NA | 0.717 | 0.773 |
|  | PPV | NA | 1.000 | 1.000 | 0.978 | 0.917 | 0.956 | 0.958 | 0.785 | 0.685 | 0.548 | 0.453 | 0.385 | 0.256 | 0.232 | 0.315 | 0.196 | NA | 0.817 | 0.572 |
|  | Mis | NA | 0.130 | 0.118 | 0.101 | 0.101 | 0.084 | 0.045 | 0.066 | 0.087 | 0.143 | 0.185 | 0.242 | 0.346 | 0.368 | 0.360 | 0.594 | NA | 0.086 | 0.228 |
| MCS  -P | Freq | 0 | 0 | 7 | 5 | 28 | 28 | 414 | 171 | 122 | 38 | 41 | 34 | 42 | 17 | 9 | 1 | 2 | 959 | 959 |
|  | Sen | NA | NA | 0.114 | 0.180 | 0.311 | 0.421 | 0.720 | 0.796 | 0.841 | 0.766 | 0.756 | 0.903 | 0.921 | 0.906 | 1.000 | 0.900 | 0.500 | 0.746 | 0.773 |
|  | PPV | NA | NA | 0.571 | 0.600 | 0.893 | 0.972 | 0.981 | 0.835 | 0.757 | 0.655 | 0.615 | 0.612 | 0.510 | 0.424 | 0.392 | 0.321 | 0.167 | 0.839 | 0.572 |
|  | Mis | NA | NA | 0.141 | 0.133 | 0.105 | 0.086 | 0.043 | 0.051 | 0.062 | 0.092 | 0.105 | 0.100 | 0.145 | 0.194 | 0.227 | 0.290 | 0.464 | 0.068 | 0.228 |
| MCHS  -P | Freq | 0 | 0 | 3 | 2 | 13 | 26 | 367 | 164 | 127 | 57 | 58 | 44 | 58 | 23 | 12 | 1 | 4 | 959 | 959 |
|  | Sen | NA | NA | 0.200 | 0.250 | 0.331 | 0.404 | 0.712 | 0.817 | 0.836 | 0.781 | 0.771 | 0.857 | 0.881 | 0.835 | 0.800 | 0.900 | 0.500 | 0.758 | 0.773 |
|  | PPV | NA | NA | 1.000 | 1.000 | 0.962 | 0.952 | 0.973 | 0.837 | 0.746 | 0.588 | 0.530 | 0.532 | 0.446 | 0.369 | 0.309 | 0.321 | 0.149 | 0.790 | 0.572 |
|  | Mis | NA | NA | 0.116 | 0.109 | 0.100 | 0.090 | 0.045 | 0.050 | 0.065 | 0.118 | 0.145 | 0.148 | 0.197 | 0.244 | 0.307 | 0.290 | 0.507 | 0.085 | 0.228 |

^a^Freq: frequency. ^b^Sen: sensitivity. ^c^PPV: positive predictive value. ^d^Mis: misclassification.

Table A12. Multinomial model: Simulation results for the true cluster model (D) and alternative hypothesis (4) using elliptical windows.

|  |  | Maximum reported cluster size (MRCS) | | | | | | | | | | | | | | | | | | Default  Setting |
| --- | --- | --- | --- | --- | --- | --- | --- | --- | --- | --- | --- | --- | --- | --- | --- | --- | --- | --- | --- | --- |
|  |  | 1% | 2% | 3% | 4% | 5% | 6% | 8% | 10% | 12% | 15% | 20% | 25% | 30% | 35% | 40% | 45% | 50% | Overall |  |
| SCIC_1_ | Freq^a^ | 0 | 1 | 9 | 13 | 31 | 36 | 313 | 57 | 47 | 36 | 79 | 156 | 86 | 25 | 15 | 22 | 45 | 971 | 971 |
|  | Sen^b^ | NA | 0.100 | 0.200 | 0.215 | 0.358 | 0.442 | 0.672 | 0.665 | 0.700 | 0.647 | 0.780 | 0.950 | 0.956 | 0.936 | 0.820 | 0.914 | 0.909 | 0.746 | 0.781 |
|  | PPV^c^ | NA | 1.000 | 1.000 | 0.974 | 0.952 | 0.964 | 0.969 | 0.797 | 0.678 | 0.571 | 0.587 | 0.580 | 0.469 | 0.354 | 0.227 | 0.206 | 0.182 | 0.711 | 0.516 |
|  | Mis^d^ | NA | 0.130 | 0.116 | 0.115 | 0.097 | 0.084 | 0.052 | 0.074 | 0.095 | 0.128 | 0.117 | 0.124 | 0.192 | 0.296 | 0.443 | 0.540 | 0.604 | 0.140 | 0.271 |
| SCIC_2_ | Freq | 0 | 0 | 6 | 11 | 31 | 36 | 310 | 67 | 53 | 36 | 60 | 93 | 66 | 32 | 31 | 43 | 96 | 971 | 971 |
|  | Sen | NA | NA | 0.200 | 0.236 | 0.403 | 0.450 | 0.711 | 0.770 | 0.777 | 0.736 | 0.750 | 0.906 | 0.888 | 0.847 | 0.700 | 0.879 | 0.848 | 0.750 | 0.781 |
|  | PPV | NA | NA | 1.000 | 0.970 | 0.957 | 0.956 | 0.958 | 0.792 | 0.700 | 0.541 | 0.488 | 0.503 | 0.400 | 0.271 | 0.191 | 0.195 | 0.174 | 0.648 | 0.516 |
|  | Mis | NA | NA | 0.116 | 0.112 | 0.091 | 0.085 | 0.048 | 0.065 | 0.084 | 0.136 | 0.165 | 0.170 | 0.242 | 0.374 | 0.465 | 0.550 | 0.597 | 0.191 | 0.271 |
| Elbow | Freq | 0 | 1 | 9 | 17 | 38 | 47 | 500 | 88 | 62 | 39 | 70 | 58 | 31 | 4 | 4 | 3 | 0 | 971 | 971 |
|  | Sen | NA | 0.200 | 0.211 | 0.218 | 0.358 | 0.477 | 0.731 | 0.722 | 0.787 | 0.767 | 0.734 | 0.831 | 0.868 | 0.950 | 0.550 | 1.000 | NA | 0.706 | 0.781 |
|  | PPV | NA | 1.000 | 1.000 | 0.951 | 0.929 | 0.952 | 0.956 | 0.741 | 0.643 | 0.506 | 0.446 | 0.432 | 0.348 | 0.327 | 0.291 | 0.415 | NA | 0.803 | 0.516 |
|  | Mis | NA | 0.116 | 0.114 | 0.115 | 0.099 | 0.081 | 0.045 | 0.081 | 0.100 | 0.155 | 0.189 | 0.217 | 0.292 | 0.322 | 0.333 | 0.213 | NA | 0.094 | 0.271 |
| MCS  -P | Freq | 0 | 3 | 6 | 12 | 34 | 38 | 428 | 143 | 81 | 44 | 43 | 61 | 49 | 15 | 9 | 4 | 1 | 971 | 971 |
|  | Sen | NA | 0.067 | 0.183 | 0.225 | 0.321 | 0.447 | 0.719 | 0.801 | 0.830 | 0.768 | 0.742 | 0.941 | 0.969 | 0.967 | 0.878 | 0.975 | 1.000 | 0.741 | 0.781 |
|  | PPV | NA | 0.667 | 0.833 | 0.868 | 0.912 | 0.940 | 0.977 | 0.831 | 0.744 | 0.618 | 0.603 | 0.641 | 0.533 | 0.497 | 0.373 | 0.394 | 0.313 | 0.836 | 0.516 |
|  | Mis | NA | 0.140 | 0.123 | 0.118 | 0.103 | 0.085 | 0.043 | 0.052 | 0.066 | 0.101 | 0.110 | 0.087 | 0.132 | 0.157 | 0.238 | 0.232 | 0.319 | 0.069 | 0.271 |
| MCHS  -P | Freq | 0 | 0 | 2 | 7 | 23 | 33 | 374 | 133 | 95 | 56 | 67 | 82 | 60 | 20 | 10 | 6 | 3 | 971 | 971 |
|  | Sen | NA | NA | 0.200 | 0.257 | 0.322 | 0.424 | 0.723 | 0.820 | 0.827 | 0.788 | 0.727 | 0.855 | 0.920 | 0.880 | 0.780 | 0.717 | 0.667 | 0.753 | 0.781 |
|  | PPV | NA | NA | 1.000 | 1.000 | 0.972 | 0.868 | 0.969 | 0.824 | 0.718 | 0.592 | 0.498 | 0.525 | 0.478 | 0.389 | 0.319 | 0.241 | 0.184 | 0.774 | 0.516 |
|  | Mis | NA | NA | 0.116 | 0.108 | 0.100 | 0.095 | 0.044 | 0.053 | 0.076 | 0.112 | 0.164 | 0.152 | 0.173 | 0.241 | 0.293 | 0.394 | 0.512 | 0.092 | 0.271 |

^a^Freq: frequency. ^b^Sen: sensitivity. ^c^PPV: positive predictive value. ^d^Mis: misclassification.

Table A13. Multinomial model: Simulation results for the true cluster model (D) and alternative hypothesis (5) using elliptical windows.

|  |  | Maximum reported cluster size (MRCS) | | | | | | | | | | | | | | | | | | Default  Setting |
| --- | --- | --- | --- | --- | --- | --- | --- | --- | --- | --- | --- | --- | --- | --- | --- | --- | --- | --- | --- | --- |
|  |  | 1% | 2% | 3% | 4% | 5% | 6% | 8% | 10% | 12% | 15% | 20% | 25% | 30% | 35% | 40% | 45% | 50% | Overall |  |
| SCIC_1_ | Freq^a^ | 0 | 0 | 4 | 6 | 20 | 27 | 437 | 150 | 89 | 36 | 40 | 71 | 47 | 21 | 8 | 17 | 13 | 986 | 986 |
|  | Sen^b^ | NA | NA | 0.175 | 0.183 | 0.395 | 0.441 | 0.742 | 0.848 | 0.812 | 0.731 | 0.785 | 0.939 | 0.966 | 0.957 | 0.738 | 0.935 | 0.769 | 0.778 | 0.793 |
|  | PPV^c^ | NA | NA | 0.875 | 0.833 | 0.947 | 0.962 | 0.971 | 0.857 | 0.732 | 0.630 | 0.556 | 0.591 | 0.479 | 0.334 | 0.206 | 0.217 | 0.164 | 0.807 | 0.629 |
|  | Mis^d^ | NA | NA | 0.123 | 0.126 | 0.091 | 0.084 | 0.041 | 0.043 | 0.071 | 0.105 | 0.131 | 0.116 | 0.180 | 0.325 | 0.440 | 0.519 | 0.585 | 0.090 | 0.198 |
| SCIC_2_ | Freq | 0 | 0 | 2 | 6 | 19 | 22 | 405 | 151 | 84 | 45 | 37 | 48 | 43 | 27 | 23 | 39 | 35 | 986 | 986 |
|  | Sen | NA | NA | 0.150 | 0.183 | 0.416 | 0.373 | 0.752 | 0.850 | 0.851 | 0.784 | 0.830 | 0.900 | 0.926 | 0.870 | 0.796 | 0.872 | 0.780 | 0.785 | 0.793 |
|  | PPV | NA | NA | 0.750 | 0.917 | 0.936 | 0.959 | 0.966 | 0.842 | 0.734 | 0.576 | 0.482 | 0.485 | 0.398 | 0.278 | 0.207 | 0.208 | 0.176 | 0.747 | 0.629 |
|  | Mis | NA | NA | 0.130 | 0.121 | 0.088 | 0.094 | 0.041 | 0.046 | 0.068 | 0.121 | 0.170 | 0.178 | 0.245 | 0.364 | 0.461 | 0.504 | 0.553 | 0.126 | 0.198 |
| Elbow | Freq | 0 | 1 | 2 | 4 | 27 | 29 | 582 | 149 | 71 | 41 | 39 | 26 | 9 | 3 | 3 | 0 | 0 | 986 | 986 |
|  | Sen | NA | 0.100 | 0.150 | 0.325 | 0.433 | 0.438 | 0.777 | 0.838 | 0.800 | 0.715 | 0.838 | 0.792 | 0.911 | 1.000 | 0.667 | NA | NA | 0.767 | 0.793 |
|  | PPV | NA | 1.000 | 0.750 | 1.000 | 0.951 | 0.964 | 0.962 | 0.834 | 0.682 | 0.511 | 0.470 | 0.411 | 0.379 | 0.240 | 0.195 | NA | NA | 0.859 | 0.629 |
|  | Mis | NA | 0.130 | 0.130 | 0.098 | 0.085 | 0.084 | 0.038 | 0.049 | 0.087 | 0.148 | 0.178 | 0.221 | 0.258 | 0.459 | 0.444 | NA | NA | 0.066 | 0.198 |
| MCS  -P | Freq | 0 | 0 | 8 | 9 | 25 | 29 | 477 | 189 | 117 | 35 | 31 | 27 | 26 | 7 | 3 | 2 | 1 | 986 | 986 |
|  | Sen | NA | NA | 0.063 | 0.233 | 0.308 | 0.431 | 0.762 | 0.862 | 0.846 | 0.814 | 0.803 | 0.919 | 0.954 | 0.986 | 0.667 | 1.000 | 1.000 | 0.774 | 0.793 |
|  | PPV | NA | NA | 0.313 | 0.806 | 0.821 | 0.896 | 0.981 | 0.870 | 0.765 | 0.658 | 0.612 | 0.598 | 0.529 | 0.464 | 0.246 | 0.364 | 0.303 | 0.867 | 0.629 |
|  | Mis | NA | NA | 0.156 | 0.121 | 0.108 | 0.088 | 0.037 | 0.038 | 0.059 | 0.089 | 0.106 | 0.109 | 0.134 | 0.168 | 0.343 | 0.254 | 0.333 | 0.056 | 0.198 |
| MCHS  -P | Freq | 0 | 0 | 1 | 4 | 12 | 13 | 426 | 199 | 135 | 56 | 43 | 37 | 36 | 13 | 5 | 3 | 3 | 986 | 986 |
|  | Sen | NA | NA | 0.100 | 0.200 | 0.308 | 0.385 | 0.758 | 0.863 | 0.862 | 0.816 | 0.770 | 0.881 | 0.922 | 0.923 | 0.580 | 0.833 | 0.600 | 0.795 | 0.793 |
|  | PPV | NA | NA | 0.500 | 0.875 | 0.864 | 0.962 | 0.973 | 0.853 | 0.759 | 0.605 | 0.513 | 0.522 | 0.461 | 0.384 | 0.207 | 0.292 | 0.169 | 0.824 | 0.629 |
|  | Mis | NA | NA | 0.145 | 0.123 | 0.109 | 0.091 | 0.039 | 0.042 | 0.060 | 0.107 | 0.150 | 0.155 | 0.186 | 0.241 | 0.394 | 0.333 | 0.507 | 0.070 | 0.198 |

^a^Freq: frequency. ^b^Sen: sensitivity. ^c^PPV: positive predictive value. ^d^Mis: misclassification.

Table A14. Multinomial model: Simulation results for the true cluster model (D) and alternative hypothesis (6) using elliptical windows.

|  |  | Maximum reported cluster size (MRCS) | | | | | | | | | | | | | | | | | | Default  Setting |
| --- | --- | --- | --- | --- | --- | --- | --- | --- | --- | --- | --- | --- | --- | --- | --- | --- | --- | --- | --- | --- |
|  |  | 1% | 2% | 3% | 4% | 5% | 6% | 8% | 10% | 12% | 15% | 20% | 25% | 30% | 35% | 40% | 45% | 50% | Overall |  |
| SCIC_1_ | Freq^a^ | 0 | 1 | 1 | 8 | 24 | 29 | 415 | 123 | 94 | 26 | 40 | 94 | 48 | 26 | 12 | 17 | 25 | 983 | 983 |
|  | Sen^b^ | NA | 0.100 | 0.200 | 0.225 | 0.379 | 0.428 | 0.739 | 0.803 | 0.817 | 0.692 | 0.785 | 0.931 | 0.975 | 0.935 | 0.708 | 0.876 | 0.872 | 0.772 | 0.790 |
|  | PPV^c^ | NA | 1.000 | 1.000 | 1.000 | 0.958 | 0.994 | 0.968 | 0.823 | 0.728 | 0.589 | 0.582 | 0.582 | 0.486 | 0.369 | 0.185 | 0.196 | 0.175 | 0.783 | 0.607 |
|  | Mis^d^ | NA | 0.130 | 0.116 | 0.112 | 0.094 | 0.083 | 0.042 | 0.054 | 0.072 | 0.117 | 0.123 | 0.121 | 0.176 | 0.294 | 0.493 | 0.536 | 0.602 | 0.104 | 0.214 |
| SCIC_2_ | Freq | 0 | 1 | 1 | 5 | 20 | 25 | 382 | 132 | 96 | 34 | 37 | 58 | 52 | 30 | 20 | 33 | 57 | 983 | 983 |
|  | Sen | NA | 0.100 | 0.200 | 0.200 | 0.355 | 0.456 | 0.741 | 0.827 | 0.833 | 0.797 | 0.859 | 0.893 | 0.929 | 0.877 | 0.725 | 0.882 | 0.844 | 0.782 | 0.790 |
|  | PPV | NA | 1.000 | 1.000 | 1.000 | 0.958 | 0.988 | 0.967 | 0.819 | 0.723 | 0.563 | 0.473 | 0.453 | 0.377 | 0.295 | 0.194 | 0.205 | 0.181 | 0.722 | 0.607 |
|  | Mis | NA | 0.130 | 0.116 | 0.116 | 0.098 | 0.081 | 0.042 | 0.053 | 0.072 | 0.127 | 0.179 | 0.199 | 0.264 | 0.362 | 0.475 | 0.511 | 0.571 | 0.143 | 0.214 |
| Elbow | Freq | 0 | 1 | 1 | 3 | 25 | 40 | 538 | 147 | 89 | 35 | 47 | 38 | 11 | 3 | 0 | 4 | 0 | 982 | 983 |
|  | Sen | NA | 0.100 | 0.200 | 0.200 | 0.364 | 0.518 | 0.767 | 0.829 | 0.783 | 0.803 | 0.800 | 0.824 | 0.891 | 1.000 | NA | 0.625 | NA | 0.761 | 0.790 |
|  | PPV | NA | 1.000 | 1.000 | 1.000 | 0.938 | 0.966 | 0.966 | 0.804 | 0.677 | 0.518 | 0.432 | 0.411 | 0.342 | 0.320 | NA | 0.503 | NA | 0.841 | 0.607 |
|  | Mis | NA | 0.130 | 0.116 | 0.116 | 0.099 | 0.074 | 0.039 | 0.056 | 0.090 | 0.147 | 0.195 | 0.223 | 0.306 | 0.372 | NA | 0.243 | NA | 0.073 | 0.214 |
| MCS  -P | Freq | 0 | 1 | 3 | 7 | 22 | 28 | 481 | 202 | 111 | 41 | 23 | 17 | 29 | 14 | 0 | 1 | 3 | 983 | 983 |
|  | Sen | NA | 0.100 | 0.000 | 0.100 | 0.323 | 0.375 | 0.755 | 0.852 | 0.849 | 0.832 | 0.774 | 0.906 | 0.986 | 0.986 | NA | 1.000 | 0.667 | 0.774 | 0.790 |
|  | PPV | NA | 1.000 | 0.000 | 0.500 | 0.869 | 0.935 | 0.983 | 0.858 | 0.768 | 0.662 | 0.624 | 0.606 | 0.524 | 0.463 | NA | 0.357 | 0.208 | 0.870 | 0.607 |
|  | Mis | NA | 0.130 | 0.174 | 0.149 | 0.106 | 0.094 | 0.038 | 0.042 | 0.059 | 0.087 | 0.103 | 0.101 | 0.137 | 0.169 | NA | 0.261 | 0.425 | 0.056 | 0.214 |
| MCHS  -P | Freq | 0 | 1 | 0 | 4 | 16 | 21 | 416 | 202 | 132 | 57 | 40 | 27 | 35 | 17 | 5 | 3 | 7 | 983 | 983 |
|  | Sen | NA | 0.100 | NA | 0.250 | 0.319 | 0.395 | 0.750 | 0.838 | 0.857 | 0.840 | 0.818 | 0.885 | 0.926 | 0.935 | 0.680 | 0.800 | 0.529 | 0.784 | 0.790 |
|  | PPV | NA | 1.000 | NA | 0.781 | 0.833 | 0.976 | 0.978 | 0.835 | 0.754 | 0.624 | 0.503 | 0.507 | 0.478 | 0.452 | 0.194 | 0.246 | 0.151 | 0.823 | 0.607 |
|  | Mis | NA | 0.130 | NA | 0.123 | 0.111 | 0.090 | 0.040 | 0.048 | 0.062 | 0.100 | 0.162 | 0.159 | 0.181 | 0.195 | 0.443 | 0.401 | 0.520 | 0.073 | 0.214 |

^a^Freq: frequency. ^b^Sen: sensitivity. ^c^PPV: positive predictive value. ^d^Mis: misclassification.

Table A15. Multinomial model: Simulation results for the true cluster model (D) and alternative hypothesis (7) using elliptical windows.

|  |  | Maximum reported cluster size (MRCS) | | | | | | | | | | | | | | | | | | Default  Setting |
| --- | --- | --- | --- | --- | --- | --- | --- | --- | --- | --- | --- | --- | --- | --- | --- | --- | --- | --- | --- | --- |
|  |  | 1% | 2% | 3% | 4% | 5% | 6% | 8% | 10% | 12% | 15% | 20% | 25% | 30% | 35% | 40% | 45% | 50% | Overall |  |
| SCIC_1_ | Freq^a^ | 0 | 1 | 3 | 7 | 18 | 30 | 457 | 154 | 94 | 52 | 45 | 49 | 32 | 14 | 7 | 9 | 19 | 991 | 991 |
|  | Sen^b^ | NA | 0.100 | 0.200 | 0.243 | 0.361 | 0.500 | 0.759 | 0.846 | 0.823 | 0.842 | 0.851 | 0.914 | 0.941 | 0.829 | 0.786 | 0.822 | 0.811 | 0.783 | 0.798 |
|  | PPV^c^ | NA | 1.000 | 1.000 | 1.000 | 0.981 | 0.980 | 0.969 | 0.852 | 0.738 | 0.612 | 0.551 | 0.564 | 0.508 | 0.319 | 0.222 | 0.201 | 0.172 | 0.821 | 0.631 |
|  | Mis^d^ | NA | 0.130 | 0.116 | 0.110 | 0.093 | 0.074 | 0.039 | 0.044 | 0.069 | 0.105 | 0.137 | 0.137 | 0.158 | 0.306 | 0.445 | 0.515 | 0.588 | 0.084 | 0.195 |
| SCIC_2_ | Freq | 0 | 0 | 2 | 5 | 13 | 30 | 412 | 140 | 86 | 54 | 48 | 47 | 32 | 33 | 19 | 32 | 38 | 991 | 991 |
|  | Sen | NA | NA | 0.200 | 0.320 | 0.354 | 0.500 | 0.761 | 0.837 | 0.851 | 0.856 | 0.863 | 0.887 | 0.931 | 0.873 | 0.816 | 0.803 | 0.800 | 0.792 | 0.798 |
|  | PPV | NA | NA | 1.000 | 1.000 | 0.974 | 0.969 | 0.964 | 0.848 | 0.731 | 0.583 | 0.484 | 0.421 | 0.370 | 0.285 | 0.220 | 0.194 | 0.178 | 0.747 | 0.631 |
|  | Mis | NA | NA | 0.116 | 0.099 | 0.095 | 0.075 | 0.040 | 0.046 | 0.069 | 0.115 | 0.172 | 0.216 | 0.269 | 0.348 | 0.443 | 0.505 | 0.561 | 0.125 | 0.195 |
| Elbow | Freq | 0 | 0 | 1 | 10 | 26 | 36 | 583 | 143 | 77 | 41 | 35 | 28 | 2 | 4 | 2 | 3 | 0 | 991 | 991 |
|  | Sen | NA | NA | 0.200 | 0.430 | 0.369 | 0.536 | 0.790 | 0.838 | 0.821 | 0.788 | 0.829 | 0.825 | 0.850 | 0.875 | 0.750 | 0.933 | NA | 0.778 | 0.798 |
|  | PPV | NA | NA | 1.000 | 1.000 | 0.981 | 0.951 | 0.962 | 0.825 | 0.664 | 0.529 | 0.424 | 0.384 | 0.472 | 0.229 | 0.172 | 0.404 | NA | 0.859 | 0.631 |
|  | Mis | NA | NA | 0.116 | 0.083 | 0.093 | 0.073 | 0.036 | 0.051 | 0.092 | 0.138 | 0.205 | 0.242 | 0.159 | 0.438 | 0.543 | 0.275 | NA | 0.066 | 0.195 |
| MCS  -P | Freq | 0 | 0 | 4 | 11 | 28 | 38 | 489 | 188 | 107 | 51 | 21 | 19 | 22 | 8 | 1 | 1 | 3 | 991 | 991 |
|  | Sen | NA | NA | 0.050 | 0.264 | 0.321 | 0.526 | 0.775 | 0.839 | 0.818 | 0.884 | 0.867 | 0.905 | 0.936 | 1.000 | 1.000 | 1.000 | 0.900 | 0.777 | 0.798 |
|  | PPV | NA | NA | 0.250 | 0.841 | 0.860 | 0.922 | 0.979 | 0.857 | 0.753 | 0.678 | 0.634 | 0.618 | 0.558 | 0.551 | 0.417 | 0.357 | 0.284 | 0.876 | 0.631 |
|  | Mis | NA | NA | 0.159 | 0.115 | 0.106 | 0.074 | 0.035 | 0.043 | 0.064 | 0.077 | 0.094 | 0.101 | 0.127 | 0.134 | 0.203 | 0.261 | 0.343 | 0.054 | 0.195 |
| MCHS  -P | Freq | 0 | 0 | 0 | 5 | 9 | 16 | 443 | 199 | 129 | 77 | 37 | 29 | 23 | 16 | 5 | 1 | 2 | 991 | 991 |
|  | Sen | NA | NA | NA | 0.240 | 0.322 | 0.500 | 0.772 | 0.844 | 0.848 | 0.892 | 0.835 | 0.862 | 0.878 | 0.869 | 0.680 | 1.000 | 0.900 | 0.804 | 0.798 |
|  | PPV | NA | NA | NA | 0.900 | 0.963 | 0.930 | 0.972 | 0.845 | 0.747 | 0.641 | 0.511 | 0.478 | 0.443 | 0.382 | 0.236 | 0.357 | 0.281 | 0.831 | 0.631 |
|  | Mis | NA | NA | NA | 0.116 | 0.100 | 0.080 | 0.037 | 0.045 | 0.064 | 0.091 | 0.155 | 0.181 | 0.202 | 0.258 | 0.388 | 0.261 | 0.348 | 0.067 | 0.195 |

^a^Freq: frequency. ^b^Sen: sensitivity. ^c^PPV: positive predictive value. ^d^Mis: misclassification.

Table A16. Multinomial model: Simulation results for the true cluster model (E) and alternative hypothesis (1) using elliptical windows.

|  |  | Maximum reported cluster size (MRCS) | | | | | | | | | | | | | | | | | | Default  Setting |
| --- | --- | --- | --- | --- | --- | --- | --- | --- | --- | --- | --- | --- | --- | --- | --- | --- | --- | --- | --- | --- |
|  |  | 1% | 2% | 3% | 4% | 5% | 6% | 8% | 10% | 12% | 15% | 20% | 25% | 30% | 35% | 40% | 45% | 50% | Overall |  |
| SCIC_1_ | Freq^a^ | 0 | 0 | 2 | 2 | 19 | 32 | 545 | 224 | 75 | 41 | 13 | 8 | 3 | 4 | 6 | 3 | 4 | 981 | 981 |
|  | Sen^b^ | NA | NA | 0.200 | 0.200 | 0.363 | 0.388 | 0.751 | 0.837 | 0.843 | 0.844 | 0.777 | 0.750 | 0.567 | 0.800 | 0.833 | 0.833 | 0.950 | 0.762 | 0.781 |
|  | PPV^c^ | NA | NA | 1.000 | 1.000 | 1.000 | 0.962 | 0.968 | 0.843 | 0.708 | 0.595 | 0.425 | 0.338 | 0.260 | 0.311 | 0.327 | 0.267 | 0.277 | 0.879 | 0.784 |
|  | Mis^d^ | NA | NA | 0.116 | 0.116 | 0.092 | 0.092 | 0.040 | 0.046 | 0.077 | 0.113 | 0.187 | 0.245 | 0.300 | 0.286 | 0.275 | 0.362 | 0.388 | 0.060 | 0.093 |
| SCIC_2_ | Freq | 0 | 0 | 2 | 3 | 20 | 32 | 511 | 202 | 76 | 51 | 32 | 20 | 14 | 6 | 5 | 2 | 5 | 981 | 981 |
|  | Sen | NA | NA | 0.200 | 0.367 | 0.370 | 0.400 | 0.751 | 0.839 | 0.841 | 0.853 | 0.859 | 0.830 | 0.836 | 0.833 | 0.840 | 0.800 | 0.920 | 0.768 | 0.781 |
|  | PPV | NA | NA | 1.000 | 1.000 | 0.982 | 0.958 | 0.969 | 0.838 | 0.698 | 0.582 | 0.407 | 0.336 | 0.300 | 0.306 | 0.306 | 0.286 | 0.298 | 0.848 | 0.784 |
|  | Mis | NA | NA | 0.116 | 0.092 | 0.093 | 0.091 | 0.040 | 0.048 | 0.080 | 0.118 | 0.207 | 0.261 | 0.303 | 0.300 | 0.304 | 0.319 | 0.354 | 0.071 | 0.093 |
| Elbow | Freq | 0 | 0 | 1 | 5 | 22 | 36 | 591 | 188 | 58 | 32 | 32 | 5 | 4 | 1 | 2 | 3 | 0 | 980 | 981 |
|  | Sen | NA | NA | 0.200 | 0.620 | 0.436 | 0.444 | 0.766 | 0.821 | 0.819 | 0.831 | 0.834 | 0.660 | 0.825 | 0.900 | 0.900 | 0.833 | NA | 0.764 | 0.781 |
|  | PPV | NA | NA | 1.000 | 1.000 | 0.935 | 0.946 | 0.963 | 0.815 | 0.656 | 0.543 | 0.393 | 0.300 | 0.302 | 0.391 | 0.354 | 0.294 | NA | 0.873 | 0.784 |
|  | Mis | NA | NA | 0.116 | 0.055 | 0.088 | 0.086 | 0.039 | 0.055 | 0.095 | 0.135 | 0.213 | 0.270 | 0.308 | 0.217 | 0.254 | 0.314 | NA | 0.061 | 0.093 |
| MCS  -P | Freq | 0 | 0 | 2 | 9 | 18 | 37 | 505 | 237 | 88 | 46 | 12 | 4 | 3 | 2 | 5 | 3 | 10 | 981 | 981 |
|  | Sen | NA | NA | 0.100 | 0.444 | 0.367 | 0.405 | 0.751 | 0.849 | 0.865 | 0.878 | 0.900 | 0.750 | 0.733 | 0.850 | 0.840 | 0.900 | 0.950 | 0.772 | 0.781 |
|  | PPV | NA | NA | 0.500 | 0.889 | 0.896 | 0.923 | 0.973 | 0.854 | 0.759 | 0.674 | 0.539 | 0.417 | 0.360 | 0.356 | 0.316 | 0.310 | 0.292 | 0.883 | 0.784 |
|  | Mis | NA | NA | 0.145 | 0.085 | 0.099 | 0.092 | 0.040 | 0.043 | 0.059 | 0.079 | 0.129 | 0.181 | 0.227 | 0.246 | 0.287 | 0.304 | 0.342 | 0.055 | 0.093 |
| MCHS  -P | Freq | 0 | 0 | 0 | 6 | 16 | 33 | 473 | 242 | 102 | 67 | 13 | 6 | 3 | 2 | 5 | 4 | 9 | 981 | 981 |
|  | Sen | NA | NA | NA | 0.583 | 0.419 | 0.418 | 0.744 | 0.845 | 0.864 | 0.864 | 0.815 | 0.717 | 0.733 | 0.850 | 0.840 | 0.800 | 0.956 | 0.776 | 0.781 |
|  | PPV | NA | NA | NA | 1.000 | 0.900 | 0.868 | 0.967 | 0.852 | 0.751 | 0.646 | 0.508 | 0.389 | 0.360 | 0.356 | 0.316 | 0.310 | 0.293 | 0.865 | 0.784 |
|  | Mis | NA | NA | NA | 0.060 | 0.095 | 0.099 | 0.042 | 0.043 | 0.061 | 0.090 | 0.143 | 0.203 | 0.227 | 0.246 | 0.287 | 0.286 | 0.341 | 0.059 | 0.093 |

^a^Freq: frequency. ^b^Sen: sensitivity. ^c^PPV: positive predictive value. ^d^Mis: misclassification.

Table A17. Multinomial model: Simulation results for the true cluster model (E) and alternative hypothesis (2) using elliptical windows.

|  |  | Maximum reported cluster size (MRCS) | | | | | | | | | | | | | | | | | | Default  Setting |
| --- | --- | --- | --- | --- | --- | --- | --- | --- | --- | --- | --- | --- | --- | --- | --- | --- | --- | --- | --- | --- |
|  |  | 1% | 2% | 3% | 4% | 5% | 6% | 8% | 10% | 12% | 15% | 20% | 25% | 30% | 35% | 40% | 45% | 50% | Overall |  |
| SCIC_1_ | Freq^a^ | 0 | 0 | 4 | 12 | 28 | 52 | 452 | 168 | 83 | 41 | 21 | 12 | 7 | 6 | 5 | 4 | 8 | 903 | 903 |
|  | Sen^b^ | NA | NA | 0.125 | 0.175 | 0.314 | 0.356 | 0.665 | 0.729 | 0.716 | 0.722 | 0.624 | 0.608 | 0.714 | 0.717 | 0.800 | 0.950 | 0.963 | 0.650 | 0.679 |
|  | PPV^c^ | NA | NA | 0.750 | 0.806 | 0.964 | 0.911 | 0.956 | 0.814 | 0.696 | 0.558 | 0.428 | 0.312 | 0.390 | 0.303 | 0.337 | 0.291 | 0.298 | 0.841 | 0.764 |
|  | Mis^d^ | NA | NA | 0.134 | 0.128 | 0.102 | 0.100 | 0.054 | 0.064 | 0.087 | 0.121 | 0.184 | 0.255 | 0.246 | 0.290 | 0.287 | 0.351 | 0.335 | 0.081 | 0.106 |
| SCIC_2_ | Freq | 0 | 0 | 4 | 12 | 27 | 51 | 423 | 167 | 85 | 51 | 31 | 23 | 9 | 7 | 3 | 2 | 8 | 903 | 903 |
|  | Sen | NA | NA | 0.125 | 0.208 | 0.315 | 0.361 | 0.670 | 0.728 | 0.734 | 0.753 | 0.700 | 0.770 | 0.678 | 0.700 | 0.867 | 1.000 | 0.963 | 0.662 | 0.679 |
|  | PPV | NA | NA | 0.750 | 0.889 | 0.963 | 0.921 | 0.957 | 0.808 | 0.698 | 0.552 | 0.434 | 0.355 | 0.263 | 0.278 | 0.305 | 0.328 | 0.298 | 0.824 | 0.764 |
|  | Mis | NA | NA | 0.134 | 0.120 | 0.102 | 0.099 | 0.053 | 0.065 | 0.085 | 0.125 | 0.183 | 0.238 | 0.322 | 0.308 | 0.319 | 0.297 | 0.335 | 0.085 | 0.106 |
| Elbow | Freq | 0 | 0 | 4 | 9 | 31 | 51 | 477 | 172 | 70 | 36 | 30 | 11 | 2 | 1 | 2 | 3 | 0 | 899 | 903 |
|  | Sen | NA | NA | 0.225 | 0.211 | 0.352 | 0.375 | 0.673 | 0.728 | 0.686 | 0.686 | 0.707 | 0.764 | 0.750 | 0.800 | 0.800 | 1.000 | NA | 0.654 | 0.679 |
|  | PPV | NA | NA | 1.000 | 0.852 | 0.924 | 0.904 | 0.948 | 0.804 | 0.677 | 0.500 | 0.405 | 0.335 | 0.317 | 0.320 | 0.322 | 0.338 | NA | 0.846 | 0.764 |
|  | Mis | NA | NA | 0.112 | 0.121 | 0.101 | 0.099 | 0.054 | 0.066 | 0.095 | 0.147 | 0.202 | 0.242 | 0.268 | 0.275 | 0.275 | 0.285 | NA | 0.077 | 0.106 |
| MCS  -P | Freq | 0 | 0 | 8 | 9 | 33 | 45 | 414 | 187 | 90 | 55 | 24 | 10 | 3 | 3 | 4 | 5 | 13 | 903 | 903 |
|  | Sen | NA | NA | 0.138 | 0.122 | 0.318 | 0.371 | 0.659 | 0.739 | 0.750 | 0.745 | 0.675 | 0.760 | 0.933 | 0.800 | 0.925 | 0.960 | 0.962 | 0.663 | 0.679 |
|  | PPV | NA | NA | 0.625 | 0.463 | 0.894 | 0.908 | 0.960 | 0.827 | 0.731 | 0.604 | 0.490 | 0.394 | 0.575 | 0.345 | 0.356 | 0.324 | 0.297 | 0.837 | 0.764 |
|  | Mis | NA | NA | 0.136 | 0.150 | 0.105 | 0.098 | 0.053 | 0.060 | 0.075 | 0.104 | 0.147 | 0.196 | 0.140 | 0.251 | 0.254 | 0.296 | 0.337 | 0.077 | 0.106 |
| MCHS  -P | Freq | 0 | 0 | 5 | 5 | 24 | 43 | 399 | 191 | 97 | 68 | 29 | 12 | 4 | 3 | 5 | 5 | 13 | 903 | 903 |
|  | Sen | NA | NA | 0.220 | 0.160 | 0.338 | 0.360 | 0.652 | 0.742 | 0.747 | 0.738 | 0.672 | 0.717 | 0.950 | 0.800 | 0.820 | 0.960 | 0.962 | 0.671 | 0.679 |
|  | PPV | NA | NA | 1.000 | 0.667 | 0.924 | 0.855 | 0.955 | 0.820 | 0.724 | 0.587 | 0.460 | 0.370 | 0.545 | 0.345 | 0.374 | 0.324 | 0.297 | 0.823 | 0.764 |
|  | Mis | NA | NA | 0.113 | 0.136 | 0.102 | 0.105 | 0.055 | 0.061 | 0.077 | 0.111 | 0.165 | 0.210 | 0.149 | 0.251 | 0.235 | 0.296 | 0.337 | 0.080 | 0.106 |

^a^Freq: frequency. ^b^Sen: sensitivity. ^c^PPV: positive predictive value. ^d^Mis: misclassification.

Table A18. Multinomial model: Simulation results for the true cluster model (E) and alternative hypothesis (3) using elliptical windows.

|  |  | Maximum reported cluster size (MRCS) | | | | | | | | | | | | | | | | | | Default  Setting |
| --- | --- | --- | --- | --- | --- | --- | --- | --- | --- | --- | --- | --- | --- | --- | --- | --- | --- | --- | --- | --- |
|  |  | 1% | 2% | 3% | 4% | 5% | 6% | 8% | 10% | 12% | 15% | 20% | 25% | 30% | 35% | 40% | 45% | 50% | Overall |  |
| SCIC_1_ | Freq^a^ | 0 | 2 | 2 | 11 | 33 | 43 | 440 | 193 | 67 | 47 | 29 | 12 | 10 | 3 | 5 | 5 | 9 | 911 | 911 |
|  | Sen^b^ | NA | 0.100 | 0.200 | 0.227 | 0.306 | 0.391 | 0.652 | 0.747 | 0.755 | 0.687 | 0.669 | 0.675 | 0.670 | 0.700 | 0.900 | 0.840 | 0.956 | 0.656 | 0.680 |
|  | PPV^c^ | NA | 1.000 | 1.000 | 0.921 | 0.975 | 0.906 | 0.962 | 0.800 | 0.698 | 0.558 | 0.406 | 0.355 | 0.280 | 0.304 | 0.349 | 0.394 | 0.293 | 0.837 | 0.756 |
|  | Mis^d^ | NA | 0.130 | 0.116 | 0.116 | 0.102 | 0.095 | 0.055 | 0.065 | 0.084 | 0.128 | 0.195 | 0.242 | 0.293 | 0.280 | 0.258 | 0.267 | 0.341 | 0.083 | 0.112 |
| SCIC_2_ | Freq | 0 | 2 | 2 | 12 | 33 | 40 | 423 | 185 | 67 | 53 | 33 | 23 | 15 | 4 | 4 | 6 | 9 | 911 | 911 |
|  | Sen | NA | 0.100 | 0.200 | 0.233 | 0.309 | 0.388 | 0.650 | 0.739 | 0.769 | 0.692 | 0.715 | 0.783 | 0.740 | 0.750 | 0.825 | 0.833 | 0.956 | 0.660 | 0.680 |
|  | PPV | NA | 1.000 | 1.000 | 0.956 | 0.970 | 0.903 | 0.963 | 0.793 | 0.694 | 0.526 | 0.395 | 0.353 | 0.289 | 0.298 | 0.271 | 0.365 | 0.293 | 0.817 | 0.756 |
|  | Mis | NA | 0.130 | 0.116 | 0.114 | 0.102 | 0.096 | 0.055 | 0.067 | 0.085 | 0.141 | 0.202 | 0.250 | 0.299 | 0.297 | 0.359 | 0.295 | 0.341 | 0.090 | 0.112 |
| Elbow | Freq | 0 | 1 | 1 | 9 | 37 | 42 | 486 | 174 | 57 | 52 | 27 | 14 | 3 | 2 | 1 | 3 | 0 | 909 | 911 |
|  | Sen | NA | 0.100 | 0.200 | 0.322 | 0.338 | 0.395 | 0.662 | 0.742 | 0.749 | 0.648 | 0.659 | 0.764 | 0.633 | 0.600 | 0.900 | 0.900 | NA | 0.654 | 0.680 |
|  | PPV | NA | 1.000 | 1.000 | 0.936 | 0.981 | 0.903 | 0.955 | 0.791 | 0.647 | 0.489 | 0.337 | 0.322 | 0.298 | 0.265 | 0.360 | 0.306 | NA | 0.842 | 0.756 |
|  | Mis | NA | 0.130 | 0.116 | 0.105 | 0.097 | 0.095 | 0.054 | 0.067 | 0.101 | 0.155 | 0.231 | 0.268 | 0.275 | 0.304 | 0.246 | 0.309 | NA | 0.081 | 0.112 |
| MCS  -P | Freq | 0 | 1 | 4 | 9 | 37 | 41 | 422 | 196 | 83 | 54 | 27 | 6 | 3 | 4 | 4 | 7 | 13 | 911 | 911 |
|  | Sen | NA | 0.100 | 0.150 | 0.233 | 0.316 | 0.398 | 0.648 | 0.744 | 0.788 | 0.737 | 0.674 | 0.717 | 0.600 | 0.850 | 0.925 | 0.914 | 0.931 | 0.664 | 0.680 |
|  | PPV | NA | 1.000 | 0.750 | 0.736 | 0.914 | 0.948 | 0.973 | 0.829 | 0.730 | 0.620 | 0.449 | 0.351 | 0.314 | 0.485 | 0.356 | 0.313 | 0.287 | 0.850 | 0.756 |
|  | Mis | NA | 0.130 | 0.130 | 0.124 | 0.103 | 0.091 | 0.054 | 0.058 | 0.072 | 0.101 | 0.165 | 0.222 | 0.246 | 0.178 | 0.254 | 0.302 | 0.347 | 0.077 | 0.112 |
| MCHS  -P | Freq | 0 | 1 | 1 | 7 | 31 | 36 | 396 | 207 | 92 | 61 | 36 | 10 | 6 | 4 | 4 | 6 | 13 | 911 | 911 |
|  | Sen | NA | 0.100 | 0.200 | 0.300 | 0.323 | 0.394 | 0.641 | 0.743 | 0.780 | 0.720 | 0.686 | 0.730 | 0.683 | 0.850 | 0.925 | 0.900 | 0.931 | 0.670 | 0.680 |
|  | PPV | NA | 1.000 | 1.000 | 0.875 | 0.959 | 0.926 | 0.968 | 0.815 | 0.718 | 0.585 | 0.450 | 0.361 | 0.365 | 0.485 | 0.356 | 0.306 | 0.287 | 0.830 | 0.756 |
|  | Mis | NA | 0.130 | 0.116 | 0.112 | 0.102 | 0.094 | 0.055 | 0.061 | 0.077 | 0.116 | 0.166 | 0.225 | 0.237 | 0.178 | 0.254 | 0.309 | 0.347 | 0.081 | 0.112 |

^a^Freq: frequency. ^b^Sen: sensitivity. ^c^PPV: positive predictive value. ^d^Mis: misclassification.

Table A19. Multinomial model: Simulation results for the true cluster model (E) and alternative hypothesis (4) using elliptical windows.

|  |  | Maximum reported cluster size (MRCS) | | | | | | | | | | | | | | | | | | Default  Setting |
| --- | --- | --- | --- | --- | --- | --- | --- | --- | --- | --- | --- | --- | --- | --- | --- | --- | --- | --- | --- | --- |
|  |  | 1% | 2% | 3% | 4% | 5% | 6% | 8% | 10% | 12% | 15% | 20% | 25% | 30% | 35% | 40% | 45% | 50% | Overall |  |
| SCIC_1_ | Freq^a^ | 0 | 2 | 9 | 22 | 47 | 56 | 461 | 154 | 69 | 32 | 21 | 13 | 8 | 1 | 11 | 5 | 6 | 917 | 917 |
|  | Sen^b^ | NA | 0.100 | 0.200 | 0.195 | 0.328 | 0.400 | 0.667 | 0.725 | 0.714 | 0.638 | 0.643 | 0.731 | 0.575 | 0.500 | 0.845 | 0.980 | 0.983 | 0.634 | 0.669 |
|  | PPV^c^ | NA | 1.000 | 1.000 | 0.847 | 0.921 | 0.946 | 0.948 | 0.790 | 0.691 | 0.479 | 0.414 | 0.407 | 0.257 | 0.208 | 0.284 | 0.336 | 0.251 | 0.840 | 0.738 |
|  | Mis^d^ | NA | 0.130 | 0.116 | 0.123 | 0.102 | 0.091 | 0.055 | 0.069 | 0.088 | 0.154 | 0.200 | 0.226 | 0.319 | 0.348 | 0.354 | 0.284 | 0.442 | 0.086 | 0.123 |
| SCIC_2_ | Freq | 0 | 2 | 8 | 21 | 43 | 57 | 428 | 149 | 65 | 41 | 37 | 23 | 19 | 2 | 10 | 5 | 7 | 917 | 917 |
|  | Sen | NA | 0.100 | 0.213 | 0.248 | 0.316 | 0.411 | 0.665 | 0.724 | 0.717 | 0.673 | 0.762 | 0.739 | 0.674 | 0.750 | 0.830 | 0.940 | 0.957 | 0.644 | 0.669 |
|  | PPV | NA | 1.000 | 1.000 | 0.925 | 0.933 | 0.949 | 0.949 | 0.778 | 0.689 | 0.481 | 0.403 | 0.358 | 0.257 | 0.260 | 0.253 | 0.336 | 0.248 | 0.812 | 0.738 |
|  | Mis | NA | 0.130 | 0.114 | 0.112 | 0.102 | 0.089 | 0.055 | 0.072 | 0.089 | 0.153 | 0.208 | 0.257 | 0.328 | 0.333 | 0.399 | 0.278 | 0.439 | 0.096 | 0.123 |
| Elbow | Freq | 0 | 2 | 8 | 21 | 45 | 64 | 484 | 131 | 68 | 35 | 32 | 9 | 9 | 0 | 4 | 3 | 0 | 915 | 917 |
|  | Sen | NA | 0.100 | 0.213 | 0.248 | 0.342 | 0.427 | 0.671 | 0.724 | 0.712 | 0.657 | 0.781 | 0.533 | 0.578 | NA | 0.825 | 0.633 | NA | 0.635 | 0.669 |
|  | PPV | NA | 1.000 | 1.000 | 0.878 | 0.932 | 0.939 | 0.941 | 0.776 | 0.650 | 0.429 | 0.402 | 0.243 | 0.221 | NA | 0.280 | 0.222 | NA | 0.837 | 0.738 |
|  | Mis | NA | 0.130 | 0.114 | 0.115 | 0.099 | 0.088 | 0.055 | 0.073 | 0.102 | 0.174 | 0.205 | 0.319 | 0.353 | NA | 0.370 | 0.295 | NA | 0.085 | 0.123 |
| MCS  -P | Freq | 0 | 4 | 11 | 19 | 46 | 52 | 428 | 158 | 91 | 46 | 16 | 4 | 5 | 5 | 11 | 11 | 10 | 917 | 917 |
|  | Sen | NA | 0.075 | 0.155 | 0.232 | 0.313 | 0.402 | 0.669 | 0.750 | 0.762 | 0.713 | 0.594 | 0.750 | 0.780 | 0.720 | 0.909 | 0.964 | 0.950 | 0.653 | 0.669 |
|  | PPV | NA | 0.750 | 0.727 | 0.779 | 0.929 | 0.962 | 0.963 | 0.823 | 0.726 | 0.582 | 0.434 | 0.661 | 0.398 | 0.316 | 0.335 | 0.331 | 0.289 | 0.847 | 0.738 |
|  | Mis | NA | 0.138 | 0.130 | 0.121 | 0.103 | 0.089 | 0.052 | 0.059 | 0.075 | 0.112 | 0.171 | 0.109 | 0.203 | 0.264 | 0.277 | 0.289 | 0.348 | 0.079 | 0.123 |
| MCHS  -P | Freq | 0 | 1 | 8 | 15 | 36 | 52 | 406 | 162 | 107 | 56 | 22 | 6 | 7 | 5 | 12 | 11 | 11 | 917 | 917 |
|  | Sen | NA | 0.100 | 0.188 | 0.240 | 0.292 | 0.390 | 0.665 | 0.743 | 0.769 | 0.666 | 0.641 | 0.683 | 0.671 | 0.720 | 0.892 | 0.964 | 0.945 | 0.659 | 0.669 |
|  | PPV | NA | 1.000 | 0.875 | 0.820 | 0.898 | 0.920 | 0.955 | 0.816 | 0.714 | 0.538 | 0.419 | 0.412 | 0.333 | 0.316 | 0.325 | 0.364 | 0.313 | 0.822 | 0.738 |
|  | Mis | NA | 0.130 | 0.121 | 0.119 | 0.109 | 0.096 | 0.054 | 0.062 | 0.078 | 0.131 | 0.177 | 0.200 | 0.251 | 0.264 | 0.287 | 0.269 | 0.327 | 0.085 | 0.123 |

^a^Freq: frequency. ^b^Sen: sensitivity. ^c^PPV: positive predictive value. ^d^Mis: misclassification.

Table A20. Multinomial model: Simulation results for the true cluster model (E) and alternative hypothesis (5) using elliptical windows.

|  |  | Maximum reported cluster size (MRCS) | | | | | | | | | | | | | | | | | | Default  Setting |
| --- | --- | --- | --- | --- | --- | --- | --- | --- | --- | --- | --- | --- | --- | --- | --- | --- | --- | --- | --- | --- |
|  |  | 1% | 2% | 3% | 4% | 5% | 6% | 8% | 10% | 12% | 15% | 20% | 25% | 30% | 35% | 40% | 45% | 50% | Overall |  |
| SCIC_1_ | Freq^a^ | 0 | 1 | 1 | 7 | 22 | 34 | 521 | 213 | 65 | 44 | 13 | 10 | 9 | 2 | 0 | 3 | 5 | 950 | 950 |
|  | Sen^b^ | NA | 0.100 | 0.200 | 0.229 | 0.327 | 0.406 | 0.717 | 0.789 | 0.845 | 0.770 | 0.869 | 0.720 | 0.644 | 0.400 | NA | 0.900 | 0.880 | 0.722 | 0.738 |
|  | PPV^c^ | NA | 1.000 | 1.000 | 1.000 | 0.964 | 0.937 | 0.962 | 0.825 | 0.717 | 0.573 | 0.495 | 0.343 | 0.286 | 0.200 | NA | 0.280 | 0.252 | 0.869 | 0.784 |
|  | Mis^d^ | NA | 0.130 | 0.116 | 0.112 | 0.100 | 0.090 | 0.046 | 0.056 | 0.072 | 0.121 | 0.159 | 0.239 | 0.283 | 0.312 | NA | 0.362 | 0.400 | 0.066 | 0.093 |
| SCIC_2_ | Freq | 0 | 1 | 1 | 6 | 20 | 31 | 494 | 204 | 66 | 55 | 29 | 19 | 13 | 3 | 0 | 3 | 5 | 950 | 950 |
|  | Sen | NA | 0.100 | 0.200 | 0.200 | 0.330 | 0.406 | 0.718 | 0.788 | 0.836 | 0.756 | 0.866 | 0.779 | 0.654 | 0.567 | NA | 0.867 | 0.880 | 0.727 | 0.738 |
|  | PPV | NA | 1.000 | 1.000 | 1.000 | 0.953 | 0.947 | 0.962 | 0.826 | 0.705 | 0.547 | 0.430 | 0.331 | 0.272 | 0.219 | NA | 0.290 | 0.252 | 0.844 | 0.784 |
|  | Mis | NA | 0.130 | 0.116 | 0.116 | 0.101 | 0.090 | 0.046 | 0.056 | 0.077 | 0.132 | 0.194 | 0.256 | 0.302 | 0.338 | NA | 0.329 | 0.400 | 0.074 | 0.093 |
| Elbow | Freq | 0 | 1 | 0 | 7 | 29 | 36 | 561 | 180 | 55 | 34 | 25 | 13 | 3 | 2 | 0 | 3 | 0 | 949 | 950 |
|  | Sen | NA | 0.100 | NA | 0.300 | 0.428 | 0.400 | 0.737 | 0.776 | 0.796 | 0.694 | 0.852 | 0.623 | 0.567 | 0.400 | NA | 0.933 | NA | 0.721 | 0.738 |
|  | PPV | NA | 1.000 | NA | 1.000 | 0.941 | 0.929 | 0.956 | 0.811 | 0.664 | 0.491 | 0.394 | 0.290 | 0.269 | 0.182 | NA | 0.314 | NA | 0.864 | 0.784 |
|  | Mis | NA | 0.130 | NA | 0.101 | 0.088 | 0.092 | 0.044 | 0.061 | 0.091 | 0.156 | 0.215 | 0.271 | 0.290 | 0.326 | NA | 0.304 | NA | 0.068 | 0.093 |
| MCS  -P | Freq | 0 | 3 | 2 | 13 | 27 | 34 | 480 | 218 | 81 | 48 | 14 | 6 | 5 | 1 | 2 | 5 | 11 | 950 | 950 |
|  | Sen | NA | 0.033 | 0.000 | 0.169 | 0.344 | 0.421 | 0.720 | 0.790 | 0.840 | 0.800 | 0.814 | 0.817 | 0.700 | 0.000 | 0.800 | 0.880 | 0.918 | 0.722 | 0.738 |
|  | PPV | NA | 0.333 | 0.000 | 0.692 | 0.900 | 0.897 | 0.974 | 0.839 | 0.741 | 0.640 | 0.529 | 0.421 | 0.364 | 0.000 | 0.326 | 0.383 | 0.278 | 0.867 | 0.784 |
|  | Mis | NA | 0.150 | 0.174 | 0.132 | 0.102 | 0.092 | 0.044 | 0.052 | 0.066 | 0.091 | 0.133 | 0.184 | 0.223 | 0.420 | 0.268 | 0.258 | 0.364 | 0.064 | 0.093 |
| MCHS  -P | Freq | 0 | 1 | 0 | 8 | 24 | 27 | 463 | 222 | 89 | 68 | 16 | 8 | 5 | 1 | 2 | 5 | 11 | 950 | 950 |
|  | Sen | NA | 0.100 | NA | 0.250 | 0.350 | 0.415 | 0.714 | 0.786 | 0.848 | 0.791 | 0.806 | 0.863 | 0.660 | 0.000 | 0.800 | 0.880 | 0.918 | 0.732 | 0.738 |
|  | PPV | NA | 1.000 | NA | 0.938 | 0.856 | 0.921 | 0.964 | 0.831 | 0.744 | 0.616 | 0.534 | 0.425 | 0.319 | 0.000 | 0.326 | 0.300 | 0.278 | 0.854 | 0.784 |
|  | Mis | NA | 0.130 | NA | 0.112 | 0.105 | 0.091 | 0.046 | 0.054 | 0.064 | 0.101 | 0.130 | 0.185 | 0.252 | 0.420 | 0.268 | 0.316 | 0.364 | 0.067 | 0.093 |

^a^Freq: frequency. ^b^Sen: sensitivity. ^c^PPV: positive predictive value. ^d^Mis: misclassification.

Table A21. Multinomial model: Simulation results for the true cluster model (E) and alternative hypothesis (6) using elliptical windows.

|  |  | Maximum reported cluster size (MRCS) | | | | | | | | | | | | | | | | | | Default  Setting |
| --- | --- | --- | --- | --- | --- | --- | --- | --- | --- | --- | --- | --- | --- | --- | --- | --- | --- | --- | --- | --- |
|  |  | 1% | 2% | 3% | 4% | 5% | 6% | 8% | 10% | 12% | 15% | 20% | 25% | 30% | 35% | 40% | 45% | 50% | Overall |  |
| SCIC_1_ | Freq^a^ | 0 | 1 | 2 | 4 | 27 | 41 | 502 | 208 | 76 | 48 | 15 | 8 | 6 | 2 | 4 | 3 | 5 | 952 | 952 |
|  | Sen^b^ | NA | 0.100 | 0.200 | 0.225 | 0.326 | 0.388 | 0.707 | 0.782 | 0.749 | 0.708 | 0.687 | 0.750 | 0.717 | 0.750 | 0.775 | 0.933 | 0.960 | 0.701 | 0.723 |
|  | PPV^c^ | NA | 1.000 | 1.000 | 1.000 | 0.934 | 0.911 | 0.959 | 0.820 | 0.715 | 0.574 | 0.423 | 0.310 | 0.299 | 0.289 | 0.288 | 0.299 | 0.376 | 0.860 | 0.776 |
|  | Mis^d^ | NA | 0.130 | 0.116 | 0.112 | 0.101 | 0.095 | 0.048 | 0.057 | 0.080 | 0.122 | 0.188 | 0.279 | 0.283 | 0.297 | 0.315 | 0.338 | 0.371 | 0.070 | 0.097 |
| SCIC_2_ | Freq | NA | 1 | 2 | 2 | 28 | 31 | 477 | 201 | 77 | 56 | 32 | 20 | 14 | 3 | 3 | 1 | 4 | 952 | 952 |
|  | Sen | NA | 0.100 | 0.200 | 0.200 | 0.336 | 0.432 | 0.704 | 0.779 | 0.753 | 0.738 | 0.734 | 0.800 | 0.800 | 0.767 | 0.733 | 1.000 | 0.950 | 0.709 | 0.723 |
|  | PPV | NA | 1.000 | 1.000 | 1.000 | 0.932 | 0.943 | 0.958 | 0.815 | 0.705 | 0.565 | 0.385 | 0.329 | 0.304 | 0.300 | 0.264 | 0.345 | 0.421 | 0.834 | 0.776 |
|  | Mis | NA | 0.130 | 0.116 | 0.116 | 0.100 | 0.087 | 0.048 | 0.059 | 0.084 | 0.127 | 0.212 | 0.264 | 0.293 | 0.290 | 0.338 | 0.275 | 0.312 | 0.078 | 0.097 |
| Elbow | Freq | NA | 1 | 2 | 6 | 33 | 37 | 537 | 191 | 59 | 35 | 31 | 12 | 5 | NA | 2 | NA | NA | 951 | 952 |
|  | Sen | NA | 0.100 | 0.200 | 0.383 | 0.394 | 0.435 | 0.724 | 0.774 | 0.700 | 0.651 | 0.719 | 0.783 | 0.680 | NA | 0.800 | NA | NA | 0.704 | 0.723 |
|  | PPV | NA | 1.000 | 1.000 | 1.000 | 0.926 | 0.923 | 0.956 | 0.798 | 0.694 | 0.480 | 0.371 | 0.327 | 0.282 | NA | 0.296 | NA | NA | 0.856 | 0.776 |
|  | Mis | NA | 0.130 | 0.116 | 0.089 | 0.093 | 0.089 | 0.046 | 0.063 | 0.091 | 0.161 | 0.220 | 0.262 | 0.296 | NA | 0.304 | NA | NA | 0.070 | 0.097 |
| MCS  -P | Freq | NA | NA | 4 | 6 | 37 | 40 | 464 | 207 | 101 | 51 | 11 | 4 | 7 | 3 | 6 | 5 | 6 | 952 | 952 |
|  | Sen | NA | NA | 0.150 | 0.233 | 0.330 | 0.390 | 0.706 | 0.800 | 0.784 | 0.735 | 0.727 | 1.000 | 0.700 | 0.900 | 0.850 | 0.920 | 0.967 | 0.709 | 0.723 |
|  | PPV | NA | NA | 0.750 | 0.833 | 0.922 | 0.879 | 0.970 | 0.844 | 0.746 | 0.622 | 0.513 | 0.478 | 0.330 | 0.355 | 0.403 | 0.317 | 0.395 | 0.868 | 0.776 |
|  | Mis | NA | NA | 0.130 | 0.118 | 0.101 | 0.097 | 0.046 | 0.050 | 0.069 | 0.101 | 0.137 | 0.159 | 0.246 | 0.251 | 0.244 | 0.299 | 0.292 | 0.065 | 0.097 |
| MCHS  -P | Freq | NA | NA | 2 | 6 | 28 | 33 | 434 | 213 | 108 | 70 | 22 | 6 | 10 | 4 | 6 | 4 | 6 | 952 | 952 |
|  | Sen | NA | NA | 0.200 | 0.317 | 0.371 | 0.406 | 0.695 | 0.793 | 0.792 | 0.744 | 0.732 | 0.967 | 0.650 | 0.825 | 0.850 | 0.975 | 0.967 | 0.715 | 0.723 |
|  | PPV | NA | NA | 1.000 | 0.944 | 0.950 | 0.859 | 0.966 | 0.828 | 0.739 | 0.598 | 0.473 | 0.476 | 0.300 | 0.322 | 0.403 | 0.334 | 0.395 | 0.844 | 0.776 |
|  | Mis | NA | NA | 0.116 | 0.104 | 0.095 | 0.097 | 0.048 | 0.054 | 0.070 | 0.110 | 0.158 | 0.159 | 0.271 | 0.279 | 0.244 | 0.286 | 0.292 | 0.070 | 0.097 |

^a^Freq: frequency. ^b^Sen: sensitivity. ^c^PPV: positive predictive value. ^d^Mis: misclassification.

Table A22. Multinomial model: Simulation results for the true cluster model (E) and alternative hypothesis (7) using elliptical windows.

|  |  | Maximum reported cluster size (MRCS) | | | | | | | | | | | | | | | | | | Default  Setting |
| --- | --- | --- | --- | --- | --- | --- | --- | --- | --- | --- | --- | --- | --- | --- | --- | --- | --- | --- | --- | --- |
|  |  | 1% | 2% | 3% | 4% | 5% | 6% | 8% | 10% | 12% | 15% | 20% | 25% | 30% | 35% | 40% | 45% | 50% | Overall |  |
| SCIC_1_ | Freq^a^ | 0 | 0 | 3 | 5 | 28 | 35 | 518 | 199 | 81 | 55 | 21 | 11 | 8 | 2 | 3 | 1 | 2 | 972 | 972 |
|  | Sen^b^ | NA | NA | 0.200 | 0.200 | 0.404 | 0.406 | 0.732 | 0.796 | 0.811 | 0.749 | 0.800 | 0.600 | 0.663 | 0.500 | 0.800 | 0.800 | 1.000 | 0.727 | 0.751 |
|  | PPV^c^ | NA | NA | 1.000 | 1.000 | 0.972 | 0.933 | 0.960 | 0.816 | 0.718 | 0.566 | 0.474 | 0.316 | 0.270 | 0.214 | 0.269 | 0.286 | 0.303 | 0.859 | 0.763 |
|  | Mis^d^ | NA | NA | 0.116 | 0.116 | 0.089 | 0.090 | 0.044 | 0.056 | 0.076 | 0.123 | 0.161 | 0.240 | 0.304 | 0.341 | 0.357 | 0.319 | 0.333 | 0.066 | 0.097 |
| SCIC_2_ | Freq | 0 | 0 | 3 | 6 | 24 | 30 | 489 | 192 | 75 | 60 | 39 | 24 | 16 | 5 | 3 | 2 | 4 | 972 | 972 |
|  | Sen | NA | NA | 0.233 | 0.300 | 0.396 | 0.423 | 0.740 | 0.799 | 0.813 | 0.777 | 0.826 | 0.708 | 0.613 | 0.740 | 0.833 | 0.850 | 0.875 | 0.738 | 0.751 |
|  | PPV | NA | NA | 1.000 | 0.943 | 0.960 | 0.936 | 0.960 | 0.816 | 0.707 | 0.563 | 0.451 | 0.315 | 0.235 | 0.256 | 0.251 | 0.275 | 0.239 | 0.828 | 0.763 |
|  | Mis | NA | NA | 0.111 | 0.106 | 0.091 | 0.087 | 0.043 | 0.056 | 0.080 | 0.126 | 0.178 | 0.261 | 0.342 | 0.345 | 0.386 | 0.348 | 0.431 | 0.077 | 0.097 |
| Elbow | Freq | 0 | 0 | 1 | 8 | 34 | 36 | 568 | 178 | 63 | 35 | 29 | 12 | 5 | 0 | 1 | 2 | 0 | 972 | 972 |
|  | Sen | NA | NA | 0.200 | 0.325 | 0.465 | 0.450 | 0.758 | 0.779 | 0.776 | 0.717 | 0.755 | 0.558 | 0.500 | NA | 0.500 | 0.400 | NA | 0.731 | 0.751 |
|  | PPV | NA | NA | 1.000 | 0.957 | 0.944 | 0.925 | 0.951 | 0.802 | 0.683 | 0.505 | 0.385 | 0.280 | 0.202 | NA | 0.147 | 0.143 | NA | 0.858 | 0.763 |
|  | Mis | NA | NA | 0.116 | 0.101 | 0.083 | 0.086 | 0.042 | 0.061 | 0.087 | 0.149 | 0.211 | 0.269 | 0.374 | NA | 0.493 | 0.304 | NA | 0.067 | 0.097 |
| MCS  -P | Freq | 0 | 2 | 2 | 11 | 38 | 46 | 472 | 197 | 98 | 58 | 23 | 14 | 4 | 3 | 0 | 1 | 3 | 972 | 972 |
|  | Sen | NA | 0.050 | 0.100 | 0.236 | 0.426 | 0.465 | 0.727 | 0.806 | 0.792 | 0.791 | 0.783 | 0.707 | 0.775 | 0.767 | NA | 0.800 | 1.000 | 0.723 | 0.751 |
|  | PPV | NA | 0.500 | 0.500 | 0.782 | 0.958 | 0.896 | 0.967 | 0.841 | 0.743 | 0.617 | 0.518 | 0.375 | 0.364 | 0.311 | NA | 0.286 | 0.319 | 0.864 | 0.763 |
|  | Mis | NA | 0.145 | 0.145 | 0.120 | 0.087 | 0.085 | 0.044 | 0.049 | 0.068 | 0.098 | 0.137 | 0.205 | 0.225 | 0.275 | NA | 0.319 | 0.309 | 0.063 | 0.097 |
| MCHS  -P | Freq | 0 | 0 | 1 | 9 | 21 | 34 | 440 | 207 | 114 | 74 | 33 | 16 | 7 | 8 | 1 | 3 | 4 | 972 | 972 |
|  | Sen | NA | NA | 0.200 | 0.267 | 0.405 | 0.435 | 0.727 | 0.807 | 0.818 | 0.796 | 0.748 | 0.681 | 0.757 | 0.825 | 1.000 | 0.767 | 0.950 | 0.740 | 0.751 |
|  | PPV | NA | NA | 1.000 | 0.867 | 0.884 | 0.823 | 0.964 | 0.832 | 0.742 | 0.610 | 0.492 | 0.353 | 0.338 | 0.324 | 0.294 | 0.443 | 0.297 | 0.834 | 0.763 |
|  | Mis | NA | NA | 0.116 | 0.114 | 0.097 | 0.097 | 0.044 | 0.052 | 0.068 | 0.103 | 0.148 | 0.221 | 0.248 | 0.283 | 0.348 | 0.193 | 0.337 | 0.068 | 0.097 |

^a^Freq: frequency. ^b^Sen: sensitivity. ^c^PPV: positive predictive value. ^d^Mis: misclassification.
