# Supplementary material for: Optimizing the maximum reported cluster size for the multinomial-based spatial scan statistic
Source: Int J Health Geogr. 2023 Nov 8;22:30. doi: 10.1186/s12942-023-00353-4 (PMC10631089; doi:10.1186/s12942-023-00353-4)
Supplement: Supplementary file 2 — Additional file 2. Simulation results for ordinal model (A23–A48). [file 12942_2023_353_MOESM2_ESM.docx]

Table A23. Ordinal model: Simulation results for true cluster model (A) with an alternative hypothesis (1) using elliptical windows.

|  |  | Maximum reported cluster size (MRCS) | | | | | | | | | | | | | | | | | | Default  Setting |
| --- | --- | --- | --- | --- | --- | --- | --- | --- | --- | --- | --- | --- | --- | --- | --- | --- | --- | --- | --- | --- |
|  |  | 1% | 2% | 3% | 4% | 5% | 6% | 8% | 10% | 12% | 15% | 20% | 25% | 30% | 35% | 40% | 45% | 50% | Overall |  |
| SCIC_1_ | Freq^a^ | 1 | 1 | 3 | 12 | 56 | 38 | 571 | 180 | 66 | 36 | 21 | 1 | 3 | 1 | 0 | 1 | 0 | 991 | 991 |
|  | Sen^b^ | 0.200 | 0.200 | 0.333 | 0.433 | 0.582 | 0.684 | 0.942 | 0.976 | 0.982 | 0.972 | 0.971 | 0.800 | 1.000 | 1.000 | NA | 1.000 | NA | 0.913 | 0.945 |
|  | PPV^c^ | 1.000 | 1.000 | 0.833 | 0.972 | 0.972 | 0.987 | 0.989 | 0.811 | 0.691 | 0.570 | 0.414 | 0.250 | 0.263 | 0.238 | NA | 0.161 | NA | 0.903 | 0.883 |
|  | Mis^d^ | 0.058 | 0.058 | 0.053 | 0.042 | 0.032 | 0.024 | 0.005 | 0.018 | 0.034 | 0.057 | 0.104 | 0.188 | 0.203 | 0.232 | NA | 0.377 | NA | 0.018 | 0.020 |
| SCIC_2_ | Freq | 0 | 3 | 5 | 23 | 58 | 43 | 564 | 170 | 68 | 31 | 20 | 2 | 3 | 0 | 0 | 1 | 0 | 991 | 991 |
|  | Sen | NA | 0.267 | 0.440 | 0.539 | 0.614 | 0.698 | 0.943 | 0.979 | 0.982 | 0.968 | 0.970 | 0.900 | 1.000 | NA | NA | 1.000 | NA | 0.909 | 0.945 |
|  | PPV | NA | 1.000 | 1.000 | 0.951 | 0.969 | 0.974 | 0.988 | 0.814 | 0.693 | 0.558 | 0.412 | 0.292 | 0.263 | NA | NA | 0.161 | NA | 0.906 | 0.883 |
|  | Mis | NA | 0.053 | 0.041 | 0.036 | 0.030 | 0.024 | 0.005 | 0.018 | 0.033 | 0.059 | 0.104 | 0.167 | 0.203 | NA | NA | 0.377 | NA | 0.017 | 0.020 |
| Gini | Freq | 0 | 0 | 0 | 13 | 29 | 29 | 564 | 192 | 71 | 44 | 33 | 3 | 5 | 3 | 0 | 3 | 2 | 991 | 991 |
|  | Sen | NA | NA | NA | 0.785 | 0.717 | 0.759 | 0.946 | 0.979 | 0.989 | 0.982 | 0.970 | 0.933 | 1.000 | 1.000 | NA | 1.000 | 1.000 | 0.944 | 0.945 |
|  | PPV | NA | NA | NA | 0.969 | 0.956 | 0.931 | 0.989 | 0.814 | 0.697 | 0.558 | 0.411 | 0.292 | 0.256 | 0.234 | NA | 0.163 | 0.156 | 0.881 | 0.883 |
|  | Mis | NA | NA | NA | 0.018 | 0.024 | 0.023 | 0.005 | 0.018 | 0.033 | 0.059 | 0.105 | 0.169 | 0.212 | 0.237 | NA | 0.372 | 0.391 | 0.020 | 0.020 |
| Elbow | Freq | 0 | 2 | 10 | 74 | 75 | 53 | 548 | 148 | 56 | 18 | 6 | 1 | 0 | 0 | 0 | 0 | 0 | 991 | 991 |
|  | Sen | NA | 0.300 | 0.660 | 0.746 | 0.669 | 0.725 | 0.945 | 0.972 | 0.986 | 0.956 | 0.933 | 0.800 | NA | NA | NA | NA | NA | 0.900 | 0.945 |
|  | PPV | NA | 1.000 | 1.000 | 0.949 | 0.942 | 0.954 | 0.984 | 0.807 | 0.694 | 0.547 | 0.380 | 0.250 | NA | NA | NA | NA | NA | 0.922 | 0.883 |
|  | Mis | NA | 0.051 | 0.025 | 0.022 | 0.028 | 0.024 | 0.005 | 0.019 | 0.033 | 0.061 | 0.116 | 0.188 | NA | NA | NA | NA | NA | 0.015 | 0.020 |
| MCS  -P | Freq | 0 | 0 | 4 | 25 | 42 | 32 | 548 | 186 | 71 | 39 | 29 | 3 | 5 | 3 | 0 | 2 | 2 | 991 | 991 |
|  | Sen | NA | NA | 0.650 | 0.736 | 0.719 | 0.744 | 0.943 | 0.975 | 0.983 | 0.979 | 0.972 | 0.933 | 1.000 | 1.000 | NA | 1.000 | 1.000 | 0.933 | 0.945 |
|  | PPV | NA | NA | 1.000 | 0.953 | 0.942 | 0.960 | 0.987 | 0.811 | 0.691 | 0.558 | 0.409 | 0.292 | 0.256 | 0.234 | NA | 0.161 | 0.156 | 0.884 | 0.883 |
|  | Mis | NA | NA | 0.025 | 0.022 | 0.025 | 0.022 | 0.005 | 0.018 | 0.034 | 0.059 | 0.105 | 0.169 | 0.212 | 0.237 | NA | 0.377 | 0.391 | 0.020 | 0.020 |
| MCHS  -P | Freq | 0 | 1 | 16 | 44 | 41 | 33 | 528 | 176 | 67 | 41 | 29 | 3 | 5 | 3 | 0 | 2 | 2 | 991 | 991 |
|  | Sen | NA | 0.400 | 0.625 | 0.764 | 0.717 | 0.752 | 0.941 | 0.976 | 0.985 | 0.980 | 0.972 | 0.933 | 1.000 | 1.000 | NA | 1.000 | 1.000 | 0.924 | 0.945 |
|  | PPV | NA | 1.000 | 1.000 | 0.961 | 0.946 | 0.946 | 0.987 | 0.811 | 0.693 | 0.569 | 0.409 | 0.292 | 0.256 | 0.234 | NA | 0.161 | 0.156 | 0.886 | 0.883 |
|  | Mis | NA | 0.043 | 0.027 | 0.020 | 0.025 | 0.023 | 0.005 | 0.018 | 0.033 | 0.057 | 0.105 | 0.169 | 0.212 | 0.237 | NA | 0.377 | 0.391 | 0.021 | 0.020 |

^a^Freq: frequency. ^b^Sen: sensitivity. ^c^PPV: positive predictive value. ^d^Mis: misclassification.

Table A24. Ordinal model: Simulation results for true cluster model (A) with an alternative hypothesis (2) using elliptical windows.

|  |  | Maximum reported cluster size (MRCS) | | | | | | | | | | | | | | | | | | Default  Setting |
| --- | --- | --- | --- | --- | --- | --- | --- | --- | --- | --- | --- | --- | --- | --- | --- | --- | --- | --- | --- | --- |
|  |  | 1% | 2% | 3% | 4% | 5% | 6% | 8% | 10% | 12% | 15% | 20% | 25% | 30% | 35% | 40% | 45% | 50% | Overall |  |
| SCIC_1_ | Freq^a^ | 0 | 0 | 3 | 16 | 55 | 61 | 504 | 155 | 84 | 48 | 32 | 3 | 4 | 2 | 3 | 2 | 0 | 972 | 972 |
|  | Sen^b^ | NA | NA | 0.400 | 0.400 | 0.564 | 0.682 | 0.927 | 0.965 | 0.971 | 0.975 | 0.981 | 1.000 | 0.950 | 1.000 | 1.000 | 1.000 | NA | 0.896 | 0.939 |
|  | PPV^c^ | NA | NA | 1.000 | 0.958 | 0.938 | 0.966 | 0.980 | 0.808 | 0.678 | 0.554 | 0.431 | 0.313 | 0.442 | 0.228 | 0.190 | 0.170 | NA | 0.874 | 0.835 |
|  | Mis^d^ | NA | NA | 0.043 | 0.045 | 0.035 | 0.025 | 0.007 | 0.019 | 0.036 | 0.060 | 0.096 | 0.159 | 0.152 | 0.246 | 0.309 | 0.355 | NA | 0.023 | 0.030 |
| SCIC_2_ | Freq | 0 | 0 | 4 | 23 | 52 | 67 | 501 | 154 | 81 | 47 | 30 | 3 | 4 | 3 | 2 | 1 | 0 | 972 | 972 |
|  | Sen | NA | NA | 0.350 | 0.487 | 0.585 | 0.681 | 0.928 | 0.966 | 0.973 | 0.974 | 0.973 | 1.000 | 0.900 | 0.933 | 1.000 | 1.000 | NA | 0.894 | 0.939 |
|  | PPV | NA | NA | 1.000 | 0.962 | 0.942 | 0.956 | 0.980 | 0.807 | 0.678 | 0.552 | 0.423 | 0.313 | 0.259 | 0.198 | 0.193 | 0.179 | NA | 0.876 | 0.835 |
|  | Mis | NA | NA | 0.047 | 0.039 | 0.033 | 0.026 | 0.007 | 0.019 | 0.036 | 0.060 | 0.101 | 0.159 | 0.196 | 0.290 | 0.304 | 0.333 | NA | 0.023 | 0.030 |
| Gini | Freq | 0 | 0 | 1 | 8 | 20 | 28 | 487 | 184 | 91 | 60 | 57 | 9 | 8 | 6 | 5 | 7 | 1 | 972 | 972 |
|  | Sen | NA | NA | 0.800 | 0.675 | 0.650 | 0.743 | 0.937 | 0.962 | 0.982 | 0.983 | 0.972 | 1.000 | 0.950 | 0.933 | 1.000 | 0.971 | 1.000 | 0.938 | 0.939 |
|  | PPV | NA | NA | 1.000 | 0.975 | 0.948 | 0.942 | 0.987 | 0.804 | 0.690 | 0.558 | 0.427 | 0.331 | 0.266 | 0.202 | 0.191 | 0.168 | 0.161 | 0.835 | 0.835 |
|  | Mis | NA | NA | 0.014 | 0.025 | 0.028 | 0.023 | 0.006 | 0.020 | 0.034 | 0.059 | 0.098 | 0.148 | 0.194 | 0.278 | 0.307 | 0.352 | 0.377 | 0.030 | 0.030 |
| Elbow | Freq | 0 | 0 | 7 | 33 | 62 | 66 | 527 | 151 | 76 | 32 | 11 | 2 | 3 | 1 | 1 | 0 | 0 | 972 | 972 |
|  | Sen | NA | NA | 0.543 | 0.667 | 0.635 | 0.703 | 0.931 | 0.954 | 0.958 | 0.963 | 0.964 | 1.000 | 0.933 | 0.800 | 1.000 | NA | NA | 0.892 | 0.939 |
|  | PPV | NA | NA | 1.000 | 0.949 | 0.928 | 0.953 | 0.974 | 0.796 | 0.664 | 0.534 | 0.402 | 0.295 | 0.250 | 0.138 | 0.200 | NA | NA | 0.891 | 0.835 |
|  | Mis | NA | NA | 0.033 | 0.027 | 0.031 | 0.025 | 0.007 | 0.021 | 0.039 | 0.065 | 0.112 | 0.174 | 0.208 | 0.377 | 0.290 | NA | NA | 0.020 | 0.030 |
| MCS  -P | Freq | 0 | 0 | 1 | 16 | 34 | 41 | 475 | 179 | 87 | 59 | 50 | 8 | 6 | 6 | 4 | 4 | 2 | 972 | 972 |
|  | Sen | NA | NA | 0.800 | 0.688 | 0.618 | 0.722 | 0.932 | 0.964 | 0.977 | 0.980 | 0.980 | 1.000 | 0.933 | 0.933 | 1.000 | 0.950 | 1.000 | 0.924 | 0.939 |
|  | PPV | NA | NA | 1.000 | 0.950 | 0.927 | 0.965 | 0.983 | 0.804 | 0.687 | 0.555 | 0.422 | 0.330 | 0.265 | 0.202 | 0.191 | 0.168 | 0.154 | 0.842 | 0.835 |
|  | Mis | NA | NA | 0.014 | 0.026 | 0.032 | 0.023 | 0.006 | 0.020 | 0.035 | 0.059 | 0.101 | 0.149 | 0.193 | 0.278 | 0.308 | 0.344 | 0.399 | 0.029 | 0.030 |
| MCHS  -P | Freq | 0 | 0 | 7 | 26 | 37 | 42 | 466 | 173 | 84 | 57 | 50 | 8 | 6 | 6 | 4 | 4 | 2 | 972 | 972 |
|  | Sen | NA | NA | 0.629 | 0.715 | 0.622 | 0.724 | 0.930 | 0.966 | 0.976 | 0.979 | 0.980 | 1.000 | 0.933 | 0.933 | 1.000 | 0.950 | 1.000 | 0.919 | 0.939 |
|  | PPV | NA | NA | 0.964 | 0.946 | 0.894 | 0.955 | 0.980 | 0.807 | 0.687 | 0.558 | 0.422 | 0.330 | 0.265 | 0.202 | 0.191 | 0.168 | 0.154 | 0.842 | 0.835 |
|  | Mis | NA | NA | 0.029 | 0.025 | 0.033 | 0.023 | 0.007 | 0.019 | 0.035 | 0.059 | 0.101 | 0.149 | 0.193 | 0.278 | 0.308 | 0.344 | 0.399 | 0.029 | 0.030 |

^a^Freq: frequency. ^b^Sen: sensitivity. ^c^PPV: positive predictive value. ^d^Mis: misclassification.

Table A25. Ordinal model: Simulation results for true cluster model (A) with an alternative hypothesis (3) using elliptical windows.

|  |  | Maximum reported cluster size (MRCS) | | | | | | | | | | | | | | | | | | Default  Setting |
| --- | --- | --- | --- | --- | --- | --- | --- | --- | --- | --- | --- | --- | --- | --- | --- | --- | --- | --- | --- | --- |
|  |  | 1% | 2% | 3% | 4% | 5% | 6% | 8% | 10% | 12% | 15% | 20% | 25% | 30% | 35% | 40% | 45% | 50% | Overall |  |
| SCIC_1_ | Freq^a^ | 0 | 2 | 8 | 11 | 57 | 56 | 464 | 175 | 90 | 42 | 21 | 6 | 2 | 3 | 2 | 2 | 1 | 942 | 942 |
|  | Sen^b^ | NA | 0.200 | 0.375 | 0.345 | 0.575 | 0.682 | 0.924 | 0.968 | 0.973 | 0.986 | 0.981 | 1.000 | 1.000 | 0.933 | 1.000 | 0.800 | 1.000 | 0.893 | 0.933 |
|  | PPV^c^ | NA | 1.000 | 1.000 | 0.833 | 0.955 | 0.949 | 0.980 | 0.802 | 0.682 | 0.568 | 0.427 | 0.318 | 0.295 | 0.226 | 0.204 | 0.138 | 0.156 | 0.871 | 0.839 |
|  | Mis^d^ | NA | 0.058 | 0.045 | 0.054 | 0.034 | 0.026 | 0.007 | 0.020 | 0.035 | 0.056 | 0.098 | 0.157 | 0.174 | 0.237 | 0.283 | 0.377 | 0.391 | 0.024 | 0.029 |
| SCIC_2_ | Freq | 0 | 3 | 8 | 13 | 52 | 57 | 472 | 173 | 87 | 42 | 20 | 6 | 1 | 3 | 2 | 2 | 1 | 942 | 942 |
|  | Sen | NA | 0.200 | 0.375 | 0.400 | 0.592 | 0.677 | 0.925 | 0.963 | 0.972 | 0.986 | 0.990 | 1.000 | 1.000 | 0.933 | 1.000 | 0.800 | 1.000 | 0.893 | 0.933 |
|  | PPV | NA | 1.000 | 1.000 | 0.905 | 0.979 | 0.959 | 0.980 | 0.796 | 0.683 | 0.563 | 0.430 | 0.318 | 0.313 | 0.226 | 0.204 | 0.138 | 0.156 | 0.875 | 0.839 |
|  | Mis | NA | 0.058 | 0.045 | 0.048 | 0.031 | 0.026 | 0.007 | 0.021 | 0.035 | 0.058 | 0.097 | 0.157 | 0.159 | 0.237 | 0.283 | 0.377 | 0.391 | 0.023 | 0.029 |
| Gini | Freq | 0 | 0 | 2 | 10 | 17 | 34 | 464 | 184 | 101 | 58 | 38 | 14 | 5 | 4 | 3 | 5 | 3 | 942 | 942 |
|  | Sen | NA | NA | 0.500 | 0.680 | 0.624 | 0.706 | 0.931 | 0.967 | 0.968 | 0.990 | 0.984 | 1.000 | 0.960 | 1.000 | 1.000 | 0.920 | 1.000 | 0.932 | 0.933 |
|  | PPV | NA | NA | 0.875 | 0.960 | 0.931 | 0.938 | 0.985 | 0.803 | 0.676 | 0.562 | 0.426 | 0.331 | 0.270 | 0.230 | 0.196 | 0.157 | 0.153 | 0.839 | 0.839 |
|  | Mis | NA | NA | 0.043 | 0.026 | 0.032 | 0.025 | 0.006 | 0.020 | 0.036 | 0.058 | 0.098 | 0.148 | 0.191 | 0.243 | 0.300 | 0.365 | 0.401 | 0.029 | 0.029 |
| Elbow | Freq | 1 | 0 | 16 | 31 | 56 | 57 | 487 | 168 | 73 | 31 | 11 | 6 | 0 | 2 | 1 | 2 | 0 | 941 | 942 |
|  | Sen | NA | NA | 0.538 | 0.684 | 0.632 | 0.695 | 0.930 | 0.963 | 0.964 | 0.974 | 0.982 | 1.000 | NA | 1.000 | 1.000 | 0.800 | NA | 0.894 | 0.933 |
|  | PPV | NA | NA | 0.969 | 0.937 | 0.959 | 0.941 | 0.977 | 0.794 | 0.671 | 0.535 | 0.413 | 0.325 | NA | 0.234 | 0.200 | 0.138 | NA | 0.886 | 0.839 |
|  | Mis | NA | NA | 0.035 | 0.026 | 0.029 | 0.026 | 0.007 | 0.022 | 0.037 | 0.065 | 0.103 | 0.152 | NA | 0.239 | 0.290 | 0.377 | NA | 0.021 | 0.029 |
| MCS  -P | Freq | 0 | 0 | 5 | 17 | 38 | 41 | 451 | 178 | 101 | 53 | 29 | 13 | 4 | 3 | 4 | 4 | 1 | 942 | 942 |
|  | Sen | NA | NA | 0.520 | 0.612 | 0.653 | 0.688 | 0.931 | 0.967 | 0.972 | 0.989 | 0.986 | 1.000 | 1.000 | 0.933 | 1.000 | 0.900 | 1.000 | 0.919 | 0.933 |
|  | PPV | NA | NA | 0.950 | 0.898 | 0.954 | 0.948 | 0.984 | 0.803 | 0.678 | 0.557 | 0.423 | 0.330 | 0.283 | 0.222 | 0.195 | 0.156 | 0.156 | 0.849 | 0.839 |
|  | Mis | NA | NA | 0.038 | 0.033 | 0.028 | 0.026 | 0.006 | 0.020 | 0.036 | 0.060 | 0.099 | 0.148 | 0.185 | 0.242 | 0.301 | 0.362 | 0.391 | 0.027 | 0.029 |
| MCHS  -P | Freq | 0 | 0 | 5 | 17 | 33 | 42 | 458 | 173 | 103 | 53 | 29 | 13 | 4 | 3 | 4 | 4 | 1 | 942 | 942 |
|  | Sen | NA | NA | 0.440 | 0.647 | 0.618 | 0.681 | 0.929 | 0.968 | 0.967 | 0.989 | 0.986 | 1.000 | 1.000 | 0.933 | 1.000 | 0.900 | 1.000 | 0.917 | 0.933 |
|  | PPV | NA | NA | 1.000 | 0.929 | 0.970 | 0.950 | 0.984 | 0.805 | 0.674 | 0.557 | 0.423 | 0.330 | 0.283 | 0.222 | 0.195 | 0.156 | 0.156 | 0.851 | 0.839 |
|  | Mis | NA | NA | 0.041 | 0.029 | 0.029 | 0.026 | 0.006 | 0.020 | 0.037 | 0.060 | 0.099 | 0.148 | 0.185 | 0.242 | 0.301 | 0.362 | 0.391 | 0.027 | 0.029 |

^a^Freq: frequency. ^b^Sen: sensitivity. ^c^PPV: positive predictive value. ^d^Mis: misclassification.

Table A26. Ordinal model: Simulation results for true cluster model (A) with an alternative hypothesis (4) using elliptical windows.

|  |  | Maximum reported cluster size (MRCS) | | | | | | | | | | | | | | | | | | Default  Setting |
| --- | --- | --- | --- | --- | --- | --- | --- | --- | --- | --- | --- | --- | --- | --- | --- | --- | --- | --- | --- | --- |
|  |  | 1% | 2% | 3% | 4% | 5% | 6% | 8% | 10% | 12% | 15% | 20% | 25% | 30% | 35% | 40% | 45% | 50% | Overall |  |
| SCIC_1_ | Freq^a^ | 0 | 4 | 8 | 15 | 49 | 55 | 499 | 158 | 89 | 40 | 20 | 7 | 5 | 1 | 3 | 0 | 2 | 955 | 955 |
|  | Sen^b^ | NA | 0.200 | 0.375 | 0.400 | 0.563 | 0.658 | 0.921 | 0.946 | 0.969 | 0.980 | 0.970 | 1.000 | 1.000 | 1.000 | 1.000 | NA | 1.000 | 0.885 | 0.925 |
|  | PPV^c^ | NA | 1.000 | 0.938 | 0.956 | 0.946 | 0.967 | 0.982 | 0.790 | 0.676 | 0.558 | 0.430 | 0.322 | 0.275 | 0.217 | 0.185 | NA | 0.154 | 0.875 | 0.844 |
|  | Mis^d^ | NA | 0.058 | 0.047 | 0.045 | 0.034 | 0.027 | 0.007 | 0.022 | 0.036 | 0.059 | 0.096 | 0.155 | 0.191 | 0.261 | 0.319 | NA | 0.399 | 0.024 | 0.029 |
| SCIC_2_ | Freq | 0 | 4 | 8 | 16 | 50 | 59 | 499 | 160 | 86 | 36 | 19 | 7 | 4 | 2 | 3 | 0 | 2 | 955 | 955 |
|  | Sen | NA | 0.200 | 0.400 | 0.425 | 0.576 | 0.668 | 0.922 | 0.944 | 0.967 | 0.978 | 0.968 | 1.000 | 1.000 | 0.900 | 1.000 | NA | 1.000 | 0.884 | 0.925 |
|  | PPV | NA | 1.000 | 1.000 | 0.946 | 0.967 | 0.962 | 0.981 | 0.787 | 0.674 | 0.564 | 0.431 | 0.322 | 0.275 | 0.180 | 0.185 | NA | 0.154 | 0.878 | 0.844 |
|  | Mis | NA | 0.058 | 0.043 | 0.044 | 0.032 | 0.027 | 0.007 | 0.023 | 0.037 | 0.058 | 0.095 | 0.155 | 0.192 | 0.312 | 0.319 | NA | 0.399 | 0.024 | 0.029 |
| Gini | Freq | 0 | 1 | 2 | 7 | 22 | 28 | 508 | 146 | 106 | 59 | 46 | 9 | 6 | 4 | 5 | 3 | 3 | 955 | 955 |
|  | Sen | NA | 0.200 | 0.600 | 0.686 | 0.627 | 0.671 | 0.924 | 0.962 | 0.979 | 0.963 | 0.978 | 0.956 | 1.000 | 0.950 | 1.000 | 1.000 | 1.000 | 0.925 | 0.925 |
|  | PPV | NA | 1.000 | 1.000 | 0.886 | 0.917 | 0.973 | 0.987 | 0.802 | 0.688 | 0.549 | 0.418 | 0.318 | 0.276 | 0.207 | 0.187 | 0.169 | 0.153 | 0.845 | 0.844 |
|  | Mis | NA | 0.058 | 0.029 | 0.031 | 0.032 | 0.025 | 0.007 | 0.020 | 0.034 | 0.062 | 0.102 | 0.155 | 0.191 | 0.275 | 0.316 | 0.357 | 0.401 | 0.029 | 0.029 |
| Elbow | Freq | 0 | 1 | 15 | 24 | 65 | 68 | 514 | 152 | 78 | 26 | 3 | 4 | 2 | 0 | 2 | 1 | 0 | 955 | 955 |
|  | Sen | NA | 0.200 | 0.520 | 0.592 | 0.628 | 0.685 | 0.924 | 0.938 | 0.967 | 0.962 | 1.000 | 1.000 | 0.900 | NA | 1.000 | 1.000 | NA | 0.879 | 0.925 |
|  | PPV | NA | 1.000 | 1.000 | 0.939 | 0.942 | 0.948 | 0.979 | 0.783 | 0.662 | 0.546 | 0.455 | 0.342 | 0.212 | NA | 0.185 | 0.161 | NA | 0.896 | 0.844 |
|  | Mis | NA | 0.058 | 0.035 | 0.033 | 0.031 | 0.027 | 0.007 | 0.023 | 0.039 | 0.062 | 0.087 | 0.141 | 0.261 | NA | 0.319 | 0.377 | NA | 0.020 | 0.029 |
| MCS  -P | Freq | 0 | 2 | 5 | 9 | 35 | 54 | 491 | 156 | 98 | 42 | 36 | 8 | 8 | 2 | 3 | 3 | 3 | 955 | 955 |
|  | Sen | NA | 0.200 | 0.520 | 0.622 | 0.651 | 0.685 | 0.922 | 0.947 | 0.971 | 0.981 | 0.983 | 1.000 | 1.000 | 0.900 | 1.000 | 1.000 | 1.000 | 0.908 | 0.925 |
|  | PPV | NA | 1.000 | 1.000 | 0.896 | 0.943 | 0.953 | 0.984 | 0.791 | 0.680 | 0.556 | 0.429 | 0.333 | 0.271 | 0.180 | 0.185 | 0.169 | 0.152 | 0.857 | 0.844 |
|  | Mis | NA | 0.058 | 0.035 | 0.034 | 0.029 | 0.027 | 0.007 | 0.022 | 0.036 | 0.059 | 0.097 | 0.147 | 0.196 | 0.312 | 0.319 | 0.357 | 0.406 | 0.027 | 0.029 |
| MCHS  -P | Freq | 0 | 6 | 21 | 22 | 41 | 50 | 467 | 149 | 95 | 41 | 36 | 9 | 7 | 2 | 3 | 3 | 3 | 955 | 955 |
|  | Sen | NA | 0.367 | 0.571 | 0.736 | 0.688 | 0.692 | 0.921 | 0.948 | 0.977 | 0.980 | 0.983 | 0.956 | 1.000 | 0.900 | 1.000 | 1.000 | 1.000 | 0.900 | 0.925 |
|  | PPV | NA | 1.000 | 1.000 | 0.955 | 0.930 | 0.961 | 0.983 | 0.790 | 0.683 | 0.551 | 0.429 | 0.317 | 0.272 | 0.180 | 0.185 | 0.169 | 0.152 | 0.859 | 0.844 |
|  | Mis | NA | 0.046 | 0.031 | 0.022 | 0.028 | 0.025 | 0.007 | 0.022 | 0.035 | 0.061 | 0.097 | 0.155 | 0.195 | 0.312 | 0.319 | 0.357 | 0.406 | 0.028 | 0.029 |

^a^Freq: frequency. ^b^Sen: sensitivity. ^c^PPV: positive predictive value. ^d^Mis: misclassification.

Table 27. Ordinal model: Simulation results for true cluster model (B) with an alternative hypothesis (1) using elliptical windows.

|  |  | Maximum reported cluster size (MRCS) | | | | | | | | | | | | | | | | | | Default  Setting |
| --- | --- | --- | --- | --- | --- | --- | --- | --- | --- | --- | --- | --- | --- | --- | --- | --- | --- | --- | --- | --- |
|  |  | 1% | 2% | 3% | 4% | 5% | 6% | 8% | 10% | 12% | 15% | 20% | 25% | 30% | 35% | 40% | 45% | 50% | Overall |  |
| SCIC_1_ | Freq^a^ | 1 | 3 | 4 | 18 | 37 | 68 | 585 | 130 | 80 | 36 | 14 | 3 | 1 | 1 | 1 | 0 | 0 | 982 | 982 |
|  | Sen^b^ | 0.200 | 0.200 | 0.300 | 0.433 | 0.584 | 0.685 | 0.900 | 0.943 | 0.978 | 0.961 | 0.943 | 0.800 | 1.000 | 1.000 | 1.000 | NA | NA | 0.874 | 0.891 |
|  | PPV^c^ | 1.000 | 1.000 | 0.875 | 0.924 | 0.973 | 0.950 | 0.980 | 0.794 | 0.683 | 0.544 | 0.406 | 0.258 | 0.278 | 0.238 | 0.200 | NA | NA | 0.899 | 0.848 |
|  | Mis^d^ | 0.058 | 0.058 | 0.054 | 0.044 | 0.031 | 0.026 | 0.009 | 0.022 | 0.035 | 0.062 | 0.108 | 0.179 | 0.188 | 0.232 | 0.290 | NA | NA | 0.020 | 0.029 |
| SCIC_2_ | Freq | 0 | 5 | 5 | 28 | 51 | 75 | 566 | 120 | 80 | 32 | 14 | 3 | 1 | 1 | 1 | 0 | 0 | 982 | 982 |
|  | Sen | NA | 0.280 | 0.360 | 0.529 | 0.667 | 0.704 | 0.901 | 0.945 | 0.975 | 0.963 | 0.943 | 0.800 | 1.000 | 1.000 | 1.000 | NA | NA | 0.871 | 0.891 |
|  | PPV | NA | 1.000 | 0.900 | 0.917 | 0.948 | 0.935 | 0.981 | 0.795 | 0.682 | 0.545 | 0.399 | 0.258 | 0.278 | 0.238 | 0.200 | NA | NA | 0.899 | 0.848 |
|  | Mis | NA | 0.052 | 0.049 | 0.038 | 0.028 | 0.026 | 0.009 | 0.022 | 0.035 | 0.062 | 0.113 | 0.179 | 0.188 | 0.232 | 0.290 | NA | NA | 0.020 | 0.029 |
| Gini | Freq | 0 | 0 | 1 | 31 | 80 | 63 | 459 | 100 | 106 | 80 | 37 | 7 | 6 | 5 | 4 | 2 | 1 | 982 | 982 |
|  | Sen | NA | NA | 0.600 | 0.690 | 0.830 | 0.743 | 0.888 | 0.926 | 0.979 | 0.978 | 0.968 | 0.886 | 1.000 | 1.000 | 1.000 | 1.000 | 1.000 | 0.893 | 0.891 |
|  | PPV | NA | NA | 1.000 | 0.967 | 0.943 | 0.931 | 0.978 | 0.784 | 0.674 | 0.562 | 0.408 | 0.283 | 0.274 | 0.224 | 0.196 | 0.170 | 0.152 | 0.845 | 0.848 |
|  | Mis | NA | NA | 0.029 | 0.024 | 0.017 | 0.024 | 0.010 | 0.024 | 0.036 | 0.058 | 0.107 | 0.170 | 0.193 | 0.252 | 0.297 | 0.355 | 0.406 | 0.029 | 0.029 |
| Elbow | Freq | 0 | 3 | 9 | 72 | 118 | 81 | 506 | 106 | 64 | 15 | 5 | 1 | 0 | 2 | 0 | 0 | 0 | 982 | 982 |
|  | Sen | NA | 0.333 | 0.533 | 0.742 | 0.812 | 0.731 | 0.897 | 0.934 | 0.959 | 0.947 | 0.920 | 0.800 | NA | 1.000 | NA | NA | NA | 0.866 | 0.891 |
|  | PPV | NA | 1.000 | 0.889 | 0.954 | 0.933 | 0.909 | 0.975 | 0.786 | 0.664 | 0.537 | 0.384 | 0.267 | NA | 0.223 | NA | NA | NA | 0.910 | 0.848 |
|  | Mis | NA | 0.048 | 0.037 | 0.021 | 0.019 | 0.026 | 0.009 | 0.023 | 0.039 | 0.064 | 0.119 | 0.174 | NA | 0.254 | NA | NA | NA | 0.019 | 0.029 |
| MCS  -P | Freq | 0 | 1 | 5 | 48 | 96 | 64 | 465 | 120 | 78 | 59 | 27 | 6 | 4 | 4 | 4 | 1 | 0 | 982 | 982 |
|  | Sen | NA | 0.400 | 0.640 | 0.733 | 0.850 | 0.731 | 0.890 | 0.940 | 0.977 | 0.973 | 0.956 | 0.867 | 1.000 | 1.000 | 1.000 | 1.000 | NA | 0.887 | 0.891 |
|  | PPV | NA | 1.000 | 0.900 | 0.951 | 0.944 | 0.921 | 0.975 | 0.796 | 0.678 | 0.557 | 0.408 | 0.278 | 0.276 | 0.225 | 0.196 | 0.179 | NA | 0.867 | 0.848 |
|  | Mis | NA | 0.043 | 0.029 | 0.022 | 0.015 | 0.026 | 0.010 | 0.022 | 0.036 | 0.059 | 0.107 | 0.171 | 0.192 | 0.250 | 0.297 | 0.333 | NA | 0.025 | 0.029 |
| MCHS  -P | Freq | 0 | 2 | 10 | 57 | 92 | 64 | 462 | 117 | 76 | 57 | 27 | 5 | 4 | 4 | 4 | 1 | 0 | 982 | 982 |
|  | Sen | NA | 0.400 | 0.580 | 0.740 | 0.835 | 0.716 | 0.887 | 0.937 | 0.976 | 0.972 | 0.956 | 0.840 | 1.000 | 1.000 | 1.000 | 1.000 | NA | 0.879 | 0.891 |
|  | PPV | NA | 1.000 | 0.950 | 0.970 | 0.928 | 0.925 | 0.977 | 0.794 | 0.677 | 0.556 | 0.405 | 0.268 | 0.276 | 0.225 | 0.196 | 0.179 | NA | 0.870 | 0.848 |
|  | Mis | NA | 0.043 | 0.032 | 0.020 | 0.018 | 0.026 | 0.010 | 0.022 | 0.036 | 0.059 | 0.109 | 0.177 | 0.192 | 0.250 | 0.297 | 0.333 | NA | 0.026 | 0.029 |

^a^Freq: frequency. ^b^Sen: sensitivity. ^c^PPV: positive predictive value. ^d^Mis: misclassification.

Table 28. Ordinal model: Simulation results for true cluster model (B) with an alternative hypothesis (2) using elliptical windows.

|  |  | Maximum reported cluster size (MRCS) | | | | | | | | | | | | | | | | | | Default  Setting |
| --- | --- | --- | --- | --- | --- | --- | --- | --- | --- | --- | --- | --- | --- | --- | --- | --- | --- | --- | --- | --- |
|  |  | 1% | 2% | 3% | 4% | 5% | 6% | 8% | 10% | 12% | 15% | 20% | 25% | 30% | 35% | 40% | 45% | 50% | Overall |  |
| SCIC_1_ | Freq^a^ | 0 | 1 | 6 | 15 | 47 | 68 | 472 | 154 | 83 | 51 | 20 | 5 | 9 | 3 | 1 | 2 | 1 | 938 | 938 |
|  | Sen^b^ | NA | 0.200 | 0.367 | 0.413 | 0.566 | 0.662 | 0.894 | 0.940 | 0.961 | 0.965 | 0.950 | 0.880 | 0.978 | 0.667 | 1.000 | 1.000 | 0.800 | 0.868 | 0.883 |
|  | PPV^c^ | NA | 1.000 | 1.000 | 0.956 | 0.938 | 0.935 | 0.968 | 0.793 | 0.666 | 0.556 | 0.399 | 0.298 | 0.269 | 0.145 | 0.179 | 0.182 | 0.114 | 0.858 | 0.796 |
|  | Mis^d^ | NA | 0.058 | 0.046 | 0.044 | 0.034 | 0.028 | 0.010 | 0.022 | 0.038 | 0.059 | 0.109 | 0.159 | 0.195 | 0.304 | 0.333 | 0.326 | 0.464 | 0.028 | 0.042 |
| SCIC_2_ | Freq | 0 | 1 | 5 | 18 | 54 | 73 | 468 | 152 | 76 | 52 | 18 | 5 | 9 | 3 | 1 | 2 | 1 | 938 | 938 |
|  | Sen | NA | 0.200 | 0.400 | 0.433 | 0.607 | 0.688 | 0.895 | 0.937 | 0.966 | 0.965 | 0.944 | 0.880 | 0.933 | 0.667 | 1.000 | 1.000 | 0.800 | 0.867 | 0.883 |
|  | PPV | NA | 1.000 | 0.950 | 0.968 | 0.936 | 0.925 | 0.968 | 0.792 | 0.670 | 0.558 | 0.400 | 0.298 | 0.257 | 0.145 | 0.179 | 0.182 | 0.114 | 0.860 | 0.796 |
|  | Mis | NA | 0.058 | 0.046 | 0.043 | 0.032 | 0.028 | 0.010 | 0.023 | 0.037 | 0.059 | 0.108 | 0.159 | 0.205 | 0.304 | 0.333 | 0.326 | 0.464 | 0.027 | 0.042 |
| Gini | Freq | 0 | 1 | 4 | 18 | 58 | 66 | 376 | 119 | 108 | 70 | 58 | 23 | 13 | 7 | 7 | 6 | 4 | 938 | 938 |
|  | Sen | NA | 0.200 | 0.350 | 0.722 | 0.710 | 0.694 | 0.884 | 0.924 | 0.972 | 0.960 | 0.962 | 0.939 | 0.985 | 0.829 | 0.943 | 1.000 | 0.950 | 0.883 | 0.883 |
|  | PPV | NA | 1.000 | 1.000 | 0.989 | 0.957 | 0.929 | 0.966 | 0.778 | 0.672 | 0.558 | 0.420 | 0.310 | 0.281 | 0.185 | 0.179 | 0.170 | 0.144 | 0.796 | 0.796 |
|  | Mis | NA | 0.058 | 0.047 | 0.021 | 0.024 | 0.027 | 0.011 | 0.025 | 0.037 | 0.059 | 0.101 | 0.158 | 0.185 | 0.277 | 0.319 | 0.355 | 0.417 | 0.041 | 0.042 |
| Elbow | Freq | 0 | 1 | 7 | 39 | 79 | 76 | 460 | 146 | 73 | 35 | 12 | 2 | 3 | 2 | 1 | 2 | 0 | 938 | 938 |
|  | Sen | NA | 0.200 | 0.486 | 0.697 | 0.714 | 0.697 | 0.893 | 0.929 | 0.940 | 0.954 | 0.933 | 0.800 | 0.600 | 0.800 | 1.000 | 1.000 | NA | 0.861 | 0.883 |
|  | PPV | NA | 1.000 | 0.964 | 0.974 | 0.924 | 0.910 | 0.964 | 0.780 | 0.647 | 0.548 | 0.400 | 0.228 | 0.176 | 0.174 | 0.179 | 0.182 | NA | 0.872 | 0.796 |
|  | Mis | NA | 0.058 | 0.039 | 0.023 | 0.025 | 0.028 | 0.010 | 0.024 | 0.042 | 0.061 | 0.107 | 0.225 | 0.251 | 0.290 | 0.333 | 0.326 | NA | 0.025 | 0.042 |
| MCS  -P | Freq | 0 | 1 | 6 | 25 | 68 | 69 | 403 | 145 | 84 | 55 | 34 | 19 | 10 | 6 | 6 | 4 | 3 | 938 | 938 |
|  | Sen | NA | 0.200 | 0.467 | 0.712 | 0.744 | 0.699 | 0.889 | 0.934 | 0.964 | 0.967 | 0.947 | 0.958 | 0.940 | 0.767 | 0.933 | 1.000 | 0.933 | 0.879 | 0.883 |
|  | PPV | NA | 1.000 | 0.958 | 0.992 | 0.953 | 0.906 | 0.966 | 0.784 | 0.664 | 0.560 | 0.411 | 0.323 | 0.261 | 0.175 | 0.179 | 0.174 | 0.141 | 0.826 | 0.796 |
|  | Mis | NA | 0.058 | 0.041 | 0.021 | 0.022 | 0.028 | 0.011 | 0.024 | 0.038 | 0.058 | 0.104 | 0.150 | 0.201 | 0.280 | 0.316 | 0.344 | 0.420 | 0.035 | 0.042 |
| MCHS  -P | Freq | 0 | 2 | 11 | 32 | 66 | 68 | 398 | 143 | 82 | 54 | 34 | 19 | 10 | 6 | 6 | 4 | 3 | 938 | 938 |
|  | Sen | NA | 0.300 | 0.545 | 0.731 | 0.733 | 0.697 | 0.888 | 0.933 | 0.963 | 0.970 | 0.947 | 0.958 | 0.940 | 0.767 | 0.933 | 1.000 | 0.933 | 0.875 | 0.883 |
|  | PPV | NA | 1.000 | 0.955 | 0.992 | 0.945 | 0.902 | 0.967 | 0.784 | 0.663 | 0.561 | 0.411 | 0.323 | 0.261 | 0.175 | 0.179 | 0.174 | 0.141 | 0.827 | 0.796 |
|  | Mis | NA | 0.051 | 0.036 | 0.020 | 0.023 | 0.029 | 0.010 | 0.024 | 0.039 | 0.058 | 0.104 | 0.150 | 0.201 | 0.280 | 0.316 | 0.344 | 0.420 | 0.035 | 0.042 |

^a^Freq: frequency. ^b^Sen: sensitivity. ^c^PPV: positive predictive value. ^d^Mis: misclassification.

Table 29. Ordinal model: Simulation results for true cluster model (B) with an alternative hypothesis (3) using elliptical windows.

|  |  | Maximum reported cluster size (MRCS) | | | | | | | | | | | | | | | | | | Default  Setting |
| --- | --- | --- | --- | --- | --- | --- | --- | --- | --- | --- | --- | --- | --- | --- | --- | --- | --- | --- | --- | --- |
|  |  | 1% | 2% | 3% | 4% | 5% | 6% | 8% | 10% | 12% | 15% | 20% | 25% | 30% | 35% | 40% | 45% | 50% | Overall |  |
| SCIC_1_ | Freq^a^ | 0 | 3 | 5 | 15 | 51 | 67 | 499 | 129 | 75 | 48 | 15 | 5 | 2 | 1 | 4 | 0 | 1 | 920 | 920 |
|  | Sen^b^ | NA | 0.200 | 0.360 | 0.400 | 0.569 | 0.672 | 0.882 | 0.926 | 0.971 | 0.963 | 0.947 | 0.920 | 1.000 | 1.000 | 1.000 | NA | 1.000 | 0.856 | 0.882 |
|  | PPV^c^ | NA | 1.000 | 1.000 | 0.844 | 0.948 | 0.929 | 0.965 | 0.785 | 0.673 | 0.546 | 0.406 | 0.299 | 0.278 | 0.227 | 0.189 | NA | 0.152 | 0.870 | 0.797 |
|  | Mis^d^ | NA | 0.058 | 0.046 | 0.049 | 0.034 | 0.027 | 0.011 | 0.024 | 0.037 | 0.062 | 0.106 | 0.162 | 0.188 | 0.246 | 0.312 | NA | 0.406 | 0.026 | 0.042 |
| SCIC_2_ | Freq | 0 | 3 | 5 | 16 | 53 | 71 | 497 | 124 | 76 | 46 | 15 | 5 | 3 | 1 | 4 | 0 | 1 | 920 | 920 |
|  | Sen | NA | 0.200 | 0.360 | 0.488 | 0.592 | 0.676 | 0.880 | 0.926 | 0.971 | 0.961 | 0.947 | 0.880 | 1.000 | 1.000 | 1.000 | NA | 1.000 | 0.856 | 0.882 |
|  | PPV | NA | 1.000 | 1.000 | 0.894 | 0.942 | 0.923 | 0.964 | 0.782 | 0.676 | 0.548 | 0.406 | 0.298 | 0.258 | 0.227 | 0.189 | NA | 0.152 | 0.870 | 0.797 |
|  | Mis | NA | 0.058 | 0.046 | 0.042 | 0.033 | 0.028 | 0.011 | 0.024 | 0.036 | 0.061 | 0.106 | 0.159 | 0.213 | 0.246 | 0.312 | NA | 0.406 | 0.026 | 0.042 |
| Gini | Freq | 0 | 0 | 3 | 12 | 55 | 62 | 388 | 107 | 107 | 80 | 46 | 19 | 15 | 7 | 9 | 5 | 5 | 920 | 920 |
|  | Sen | NA | NA | 0.333 | 0.583 | 0.738 | 0.668 | 0.874 | 0.914 | 0.981 | 0.985 | 0.965 | 0.947 | 0.960 | 0.971 | 1.000 | 0.960 | 0.920 | 0.883 | 0.882 |
|  | PPV | NA | NA | 1.000 | 0.944 | 0.966 | 0.918 | 0.964 | 0.768 | 0.678 | 0.566 | 0.415 | 0.310 | 0.263 | 0.218 | 0.189 | 0.167 | 0.137 | 0.796 | 0.797 |
|  | Mis | NA | NA | 0.048 | 0.033 | 0.021 | 0.029 | 0.012 | 0.027 | 0.036 | 0.057 | 0.103 | 0.157 | 0.199 | 0.255 | 0.312 | 0.351 | 0.426 | 0.042 | 0.042 |
| Elbow | Freq | 0 | 0 | 7 | 35 | 81 | 76 | 475 | 127 | 70 | 32 | 9 | 2 | 1 | 2 | 2 | 1 | 0 | 920 | 920 |
|  | Sen | NA | NA | 0.457 | 0.657 | 0.711 | 0.687 | 0.880 | 0.910 | 0.963 | 0.950 | 0.911 | 1.000 | 1.000 | 1.000 | 1.000 | 1.000 | NA | 0.852 | 0.882 |
|  | PPV | NA | NA | 1.000 | 0.933 | 0.939 | 0.917 | 0.960 | 0.767 | 0.662 | 0.530 | 0.377 | 0.292 | 0.278 | 0.227 | 0.179 | 0.172 | NA | 0.878 | 0.797 |
|  | Mis | NA | NA | 0.039 | 0.028 | 0.025 | 0.027 | 0.012 | 0.027 | 0.039 | 0.066 | 0.118 | 0.188 | 0.188 | 0.246 | 0.333 | 0.348 | NA | 0.024 | 0.042 |
| MCS  -P | Freq | 0 | 1 | 4 | 27 | 66 | 67 | 412 | 122 | 90 | 60 | 36 | 15 | 6 | 4 | 6 | 0 | 4 | 920 | 920 |
|  | Sen | NA | 0.200 | 0.350 | 0.652 | 0.764 | 0.672 | 0.877 | 0.923 | 0.976 | 0.990 | 0.950 | 0.947 | 0.967 | 1.000 | 1.000 | NA | 0.950 | 0.874 | 0.882 |
|  | PPV | NA | 1.000 | 1.000 | 0.942 | 0.961 | 0.921 | 0.960 | 0.775 | 0.674 | 0.564 | 0.405 | 0.318 | 0.258 | 0.231 | 0.190 | NA | 0.141 | 0.830 | 0.797 |
|  | Mis | NA | 0.058 | 0.047 | 0.028 | 0.020 | 0.028 | 0.012 | 0.025 | 0.036 | 0.057 | 0.107 | 0.152 | 0.205 | 0.243 | 0.309 | NA | 0.424 | 0.033 | 0.042 |
| MCHS  -P | Freq | 0 | 2 | 8 | 13 | 48 | 65 | 440 | 125 | 89 | 60 | 36 | 15 | 5 | 4 | 6 | 0 | 4 | 920 | 920 |
|  | Sen | NA | 0.100 | 0.450 | 0.523 | 0.675 | 0.665 | 0.880 | 0.923 | 0.975 | 0.990 | 0.950 | 0.947 | 0.960 | 1.000 | 1.000 | NA | 0.950 | 0.872 | 0.882 |
|  | PPV | NA | 0.500 | 1.000 | 0.921 | 0.956 | 0.926 | 0.963 | 0.776 | 0.674 | 0.564 | 0.405 | 0.318 | 0.267 | 0.231 | 0.190 | NA | 0.141 | 0.831 | 0.797 |
|  | Mis | NA | 0.072 | 0.040 | 0.038 | 0.027 | 0.028 | 0.011 | 0.025 | 0.036 | 0.057 | 0.107 | 0.152 | 0.194 | 0.243 | 0.309 | NA | 0.424 | 0.033 | 0.042 |

^a^Freq: frequency. ^b^Sen: sensitivity. ^c^PPV: positive predictive value. ^d^Mis: misclassification.

Table 30. Ordinal model: Simulation results for true cluster model (B) with an alternative hypothesis (4) using elliptical windows.

|  |  | Maximum reported cluster size (MRCS) | | | | | | | | | | | | | | | | | | Default  Setting |
| --- | --- | --- | --- | --- | --- | --- | --- | --- | --- | --- | --- | --- | --- | --- | --- | --- | --- | --- | --- | --- |
|  |  | 1% | 2% | 3% | 4% | 5% | 6% | 8% | 10% | 12% | 15% | 20% | 25% | 30% | 35% | 40% | 45% | 50% | Overall |  |
| SCIC_1_ | Freq^a^ | 0 | 7 | 7 | 22 | 44 | 80 | 487 | 123 | 80 | 42 | 17 | 8 | 3 | 1 | 1 | 0 | 1 | 923 | 923 |
|  | Sen^b^ | NA | 0.200 | 0.371 | 0.418 | 0.568 | 0.660 | 0.886 | 0.920 | 0.958 | 0.967 | 0.918 | 0.900 | 1.000 | 0.000 | 0.800 | NA | 1.000 | 0.846 | 0.873 |
|  | PPV^c^ | NA | 1.000 | 0.929 | 0.961 | 0.947 | 0.930 | 0.963 | 0.778 | 0.671 | 0.552 | 0.395 | 0.292 | 0.261 | 0.000 | 0.143 | NA | 0.147 | 0.869 | 0.803 |
|  | Mis^d^ | NA | 0.058 | 0.048 | 0.044 | 0.034 | 0.028 | 0.011 | 0.025 | 0.038 | 0.060 | 0.109 | 0.165 | 0.208 | 0.391 | 0.362 | NA | 0.420 | 0.026 | 0.040 |
| SCIC_2_ | Freq | 0 | 7 | 9 | 29 | 50 | 78 | 483 | 120 | 76 | 41 | 17 | 7 | 4 | 1 | 0 | 0 | 1 | 923 | 923 |
|  | Sen | NA | 0.200 | 0.400 | 0.441 | 0.600 | 0.667 | 0.888 | 0.920 | 0.961 | 0.966 | 0.929 | 0.886 | 0.950 | 0.000 | NA | NA | 1.000 | 0.843 | 0.873 |
|  | PPV | NA | 1.000 | 0.944 | 0.956 | 0.950 | 0.936 | 0.964 | 0.776 | 0.674 | 0.550 | 0.396 | 0.292 | 0.241 | 0.000 | NA | NA | 0.147 | 0.873 | 0.803 |
|  | Mis | NA | 0.058 | 0.045 | 0.043 | 0.032 | 0.028 | 0.011 | 0.025 | 0.037 | 0.061 | 0.109 | 0.164 | 0.225 | 0.391 | NA | NA | 0.420 | 0.026 | 0.040 |
| Gini | Freq | 0 | 2 | 1 | 32 | 49 | 62 | 390 | 99 | 106 | 84 | 53 | 12 | 13 | 7 | 5 | 4 | 4 | 923 | 923 |
|  | Sen | NA | 0.200 | 0.400 | 0.663 | 0.706 | 0.674 | 0.873 | 0.901 | 0.974 | 0.967 | 0.940 | 0.917 | 0.923 | 0.857 | 0.960 | 1.000 | 1.000 | 0.871 | 0.873 |
|  | PPV | NA | 1.000 | 1.000 | 0.971 | 0.962 | 0.938 | 0.964 | 0.757 | 0.675 | 0.558 | 0.409 | 0.309 | 0.247 | 0.192 | 0.181 | 0.168 | 0.154 | 0.803 | 0.803 |
|  | Mis | NA | 0.058 | 0.043 | 0.026 | 0.024 | 0.027 | 0.012 | 0.029 | 0.036 | 0.059 | 0.105 | 0.155 | 0.212 | 0.271 | 0.319 | 0.359 | 0.399 | 0.040 | 0.040 |
| Elbow | Freq | 0 | 4 | 10 | 53 | 79 | 86 | 474 | 111 | 61 | 29 | 9 | 3 | 3 | 0 | 0 | 0 | 0 | 922 | 923 |
|  | Sen | NA | 0.200 | 0.500 | 0.657 | 0.699 | 0.672 | 0.889 | 0.912 | 0.944 | 0.966 | 0.911 | 0.800 | 0.667 | NA | NA | NA | NA | 0.840 | 0.873 |
|  | PPV | NA | 1.000 | 1.000 | 0.963 | 0.940 | 0.916 | 0.961 | 0.766 | 0.664 | 0.535 | 0.386 | 0.239 | 0.163 | NA | NA | NA | NA | 0.889 | 0.803 |
|  | Mis | NA | 0.058 | 0.036 | 0.027 | 0.026 | 0.029 | 0.011 | 0.027 | 0.039 | 0.064 | 0.113 | 0.203 | 0.275 | NA | NA | NA | NA | 0.023 | 0.040 |
| MCS  -P | Freq | 0 | 2 | 5 | 42 | 64 | 72 | 408 | 115 | 89 | 59 | 36 | 11 | 7 | 6 | 3 | 2 | 2 | 923 | 923 |
|  | Sen | NA | 0.200 | 0.440 | 0.657 | 0.719 | 0.672 | 0.882 | 0.911 | 0.957 | 0.969 | 0.944 | 0.873 | 0.943 | 0.833 | 0.933 | 1.000 | 1.000 | 0.860 | 0.873 |
|  | PPV | NA | 1.000 | 1.000 | 0.967 | 0.938 | 0.935 | 0.964 | 0.770 | 0.666 | 0.550 | 0.412 | 0.293 | 0.243 | 0.188 | 0.176 | 0.161 | 0.154 | 0.835 | 0.803 |
|  | Mis | NA | 0.058 | 0.041 | 0.027 | 0.024 | 0.028 | 0.011 | 0.027 | 0.038 | 0.061 | 0.103 | 0.163 | 0.219 | 0.273 | 0.324 | 0.377 | 0.399 | 0.033 | 0.040 |
| MCHS  -P | Freq | 0 | 4 | 14 | 57 | 67 | 73 | 388 | 111 | 84 | 58 | 36 | 11 | 7 | 6 | 3 | 2 | 2 | 923 | 923 |
|  | Sen | NA | 0.300 | 0.529 | 0.698 | 0.731 | 0.679 | 0.880 | 0.910 | 0.957 | 0.969 | 0.944 | 0.873 | 0.943 | 0.833 | 0.933 | 1.000 | 1.000 | 0.854 | 0.873 |
|  | PPV | NA | 0.917 | 1.000 | 0.964 | 0.937 | 0.928 | 0.965 | 0.768 | 0.663 | 0.550 | 0.410 | 0.293 | 0.243 | 0.188 | 0.176 | 0.161 | 0.154 | 0.837 | 0.803 |
|  | Mis | NA | 0.054 | 0.034 | 0.024 | 0.024 | 0.028 | 0.011 | 0.027 | 0.039 | 0.061 | 0.104 | 0.163 | 0.219 | 0.273 | 0.324 | 0.377 | 0.399 | 0.034 | 0.040 |

^a^Freq: frequency. ^b^Sen: sensitivity. ^c^PPV: positive predictive value. ^d^Mis: misclassification.

Table A31. Ordinal model: Simulation results for true cluster model (C) with an alternative hypothesis (1) using elliptical windows.

|  |  | Maximum reported cluster size (MRCS) | | | | | | | | | | | | | | | | | | Default  Setting |
| --- | --- | --- | --- | --- | --- | --- | --- | --- | --- | --- | --- | --- | --- | --- | --- | --- | --- | --- | --- | --- |
|  |  | 1% | 2% | 3% | 4% | 5% | 6% | 8% | 10% | 12% | 15% | 20% | 25% | 30% | 35% | 40% | 45% | 50% | Overall |  |
| SCIC_1_ | Freq^a^ | 0 | 3 | 1 | 6 | 9 | 34 | 151 | 228 | 412 | 73 | 41 | 14 | 11 | 9 | 3 | 2 | 0 | 997 | 997 |
|  | Sen^b^ | NA | 0.100 | 0.200 | 0.333 | 0.311 | 0.476 | 0.603 | 0.729 | 0.783 | 0.747 | 0.720 | 0.807 | 0.755 | 0.922 | 0.967 | 0.700 | NA | 0.719 | 0.743 |
|  | PPV^c^ | NA | 1.000 | 1.000 | 1.000 | 0.978 | 0.971 | 0.961 | 0.943 | 0.955 | 0.781 | 0.663 | 0.563 | 0.686 | 0.496 | 0.372 | 0.650 | NA | 0.915 | 0.825 |
|  | Mis^d^ | NA | 0.130 | 0.116 | 0.097 | 0.101 | 0.078 | 0.062 | 0.046 | 0.038 | 0.069 | 0.099 | 0.123 | 0.119 | 0.169 | 0.242 | 0.196 | NA | 0.055 | 0.079 |
| SCIC_2_ | Freq | 0 | 3 | 3 | 10 | 33 | 75 | 178 | 222 | 385 | 54 | 17 | 7 | 3 | 5 | 1 | 1 | 0 | 997 | 997 |
|  | Sen | NA | 0.100 | 0.300 | 0.400 | 0.479 | 0.581 | 0.670 | 0.764 | 0.813 | 0.767 | 0.700 | 0.786 | 0.867 | 0.940 | 0.900 | 0.900 | NA | 0.737 | 0.743 |
|  | PPV | NA | 1.000 | 1.000 | 0.980 | 0.977 | 0.964 | 0.947 | 0.935 | 0.953 | 0.778 | 0.626 | 0.578 | 0.433 | 0.496 | 0.346 | 0.300 | NA | 0.927 | 0.825 |
|  | Mis | NA | 0.130 | 0.101 | 0.088 | 0.078 | 0.064 | 0.054 | 0.043 | 0.034 | 0.067 | 0.105 | 0.122 | 0.184 | 0.171 | 0.261 | 0.319 | NA | 0.050 | 0.079 |
| Gini | Freq | 0 | 1 | 1 | 5 | 31 | 95 | 135 | 137 | 333 | 63 | 45 | 48 | 31 | 28 | 29 | 8 | 7 | 997 | 997 |
|  | Sen | NA | 0.100 | 0.200 | 0.360 | 0.594 | 0.685 | 0.710 | 0.797 | 0.807 | 0.783 | 0.751 | 0.823 | 0.865 | 0.943 | 0.962 | 0.975 | 1.000 | 0.780 | 0.743 |
|  | PPV | NA | 1.000 | 1.000 | 1.000 | 0.971 | 0.963 | 0.921 | 0.917 | 0.945 | 0.769 | 0.613 | 0.545 | 0.473 | 0.452 | 0.378 | 0.339 | 0.305 | 0.841 | 0.825 |
|  | Mis | NA | 0.130 | 0.116 | 0.093 | 0.062 | 0.050 | 0.052 | 0.041 | 0.036 | 0.066 | 0.106 | 0.128 | 0.164 | 0.186 | 0.236 | 0.281 | 0.331 | 0.069 | 0.079 |
| Elbow | Freq | 0 | 1 | 6 | 31 | 129 | 176 | 223 | 185 | 213 | 28 | 3 | 1 | 1 | 0 | 0 | 0 | 0 | 997 | 997 |
|  | Sen | NA | 0.100 | 0.333 | 0.497 | 0.651 | 0.673 | 0.692 | 0.763 | 0.800 | 0.757 | 0.700 | 0.800 | 0.900 | NA | NA | NA | NA | 0.713 | 0.743 |
|  | PPV | NA | 1.000 | 1.000 | 0.984 | 0.965 | 0.953 | 0.934 | 0.927 | 0.941 | 0.728 | 0.679 | 0.500 | 0.474 | NA | NA | NA | NA | 0.936 | 0.825 |
|  | Mis | NA | 0.130 | 0.097 | 0.074 | 0.054 | 0.053 | 0.053 | 0.044 | 0.038 | 0.079 | 0.092 | 0.145 | 0.159 | NA | NA | NA | NA | 0.050 | 0.079 |
| MCS  -P | Freq | 0 | 1 | 3 | 15 | 75 | 143 | 173 | 166 | 311 | 47 | 23 | 14 | 12 | 8 | 3 | 2 | 1 | 997 | 997 |
|  | Sen | NA | 0.100 | 0.300 | 0.440 | 0.599 | 0.683 | 0.708 | 0.793 | 0.824 | 0.768 | 0.717 | 0.836 | 0.858 | 0.963 | 0.933 | 0.950 | 1.000 | 0.751 | 0.743 |
|  | PPV | NA | 1.000 | 1.000 | 0.977 | 0.966 | 0.953 | 0.935 | 0.930 | 0.941 | 0.757 | 0.617 | 0.559 | 0.490 | 0.541 | 0.346 | 0.329 | 0.294 | 0.909 | 0.825 |
|  | Mis | NA | 0.130 | 0.101 | 0.083 | 0.061 | 0.051 | 0.050 | 0.040 | 0.034 | 0.071 | 0.106 | 0.121 | 0.158 | 0.150 | 0.266 | 0.290 | 0.348 | 0.052 | 0.079 |
| MCHS  -P | Freq | 0 | 1 | 7 | 33 | 92 | 116 | 179 | 168 | 283 | 53 | 24 | 15 | 13 | 8 | 2 | 2 | 1 | 997 | 997 |
|  | Sen | NA | 0.100 | 0.329 | 0.455 | 0.596 | 0.662 | 0.703 | 0.789 | 0.809 | 0.783 | 0.758 | 0.847 | 0.862 | 0.975 | 0.950 | 0.950 | 1.000 | 0.735 | 0.743 |
|  | PPV | NA | 1.000 | 1.000 | 0.990 | 0.974 | 0.946 | 0.933 | 0.937 | 0.939 | 0.765 | 0.611 | 0.554 | 0.482 | 0.489 | 0.346 | 0.329 | 0.294 | 0.908 | 0.825 |
|  | Mis | NA | 0.130 | 0.097 | 0.080 | 0.061 | 0.055 | 0.051 | 0.039 | 0.037 | 0.068 | 0.106 | 0.122 | 0.163 | 0.172 | 0.268 | 0.290 | 0.348 | 0.055 | 0.079 |

^a^Freq: frequency. ^b^Sen: sensitivity. ^c^PPV: positive predictive value. ^d^Mis: misclassification.

Table A32. Ordinal model: Simulation results for true cluster model (C) with an alternative hypothesis (2) using elliptical windows.

|  |  | Maximum reported cluster size (MRCS) | | | | | | | | | | | | | | | | | | Default  Setting |
| --- | --- | --- | --- | --- | --- | --- | --- | --- | --- | --- | --- | --- | --- | --- | --- | --- | --- | --- | --- | --- |
|  |  | 1% | 2% | 3% | 4% | 5% | 6% | 8% | 10% | 12% | 15% | 20% | 25% | 30% | 35% | 40% | 45% | 50% | Overall |  |
| SCIC_1_ | Freq^a^ | 0 | 4 | 1 | 11 | 25 | 48 | 142 | 208 | 348 | 86 | 49 | 22 | 14 | 12 | 8 | 5 | 1 | 984 | 984 |
|  | Sen^b^ | NA | 0.100 | 0.200 | 0.273 | 0.308 | 0.433 | 0.558 | 0.650 | 0.730 | 0.698 | 0.735 | 0.800 | 0.857 | 0.892 | 0.975 | 0.900 | 1.000 | 0.661 | 0.714 |
|  | PPV^c^ | NA | 1.000 | 1.000 | 1.000 | 0.920 | 0.924 | 0.953 | 0.931 | 0.932 | 0.734 | 0.626 | 0.538 | 0.495 | 0.455 | 0.371 | 0.385 | 0.294 | 0.874 | 0.777 |
|  | Mis^d^ | NA | 0.130 | 0.116 | 0.105 | 0.104 | 0.088 | 0.068 | 0.058 | 0.048 | 0.081 | 0.103 | 0.132 | 0.156 | 0.194 | 0.245 | 0.258 | 0.348 | 0.071 | 0.097 |
| SCIC_2_ | Freq | 0 | 3 | 2 | 17 | 43 | 71 | 172 | 199 | 319 | 76 | 39 | 14 | 11 | 8 | 5 | 4 | 1 | 984 | 984 |
|  | Sen | NA | 0.100 | 0.250 | 0.329 | 0.435 | 0.530 | 0.610 | 0.671 | 0.755 | 0.707 | 0.736 | 0.821 | 0.873 | 0.913 | 0.960 | 0.950 | 1.000 | 0.673 | 0.714 |
|  | PPV | NA | 1.000 | 1.000 | 0.985 | 0.944 | 0.926 | 0.936 | 0.924 | 0.933 | 0.735 | 0.619 | 0.515 | 0.463 | 0.401 | 0.359 | 0.322 | 0.294 | 0.883 | 0.777 |
|  | Mis | NA | 0.130 | 0.109 | 0.098 | 0.085 | 0.075 | 0.063 | 0.057 | 0.045 | 0.080 | 0.104 | 0.140 | 0.166 | 0.212 | 0.255 | 0.297 | 0.348 | 0.067 | 0.097 |
| Gini | Freq | 0 | 1 | 0 | 8 | 22 | 62 | 112 | 136 | 290 | 77 | 78 | 57 | 46 | 29 | 36 | 21 | 9 | 984 | 984 |
|  | Sen | NA | 0.100 | NA | 0.338 | 0.418 | 0.589 | 0.610 | 0.705 | 0.756 | 0.703 | 0.749 | 0.814 | 0.865 | 0.931 | 0.978 | 0.957 | 0.978 | 0.733 | 0.714 |
|  | PPV | NA | 1.000 | NA | 1.000 | 0.966 | 0.944 | 0.925 | 0.910 | 0.942 | 0.720 | 0.606 | 0.520 | 0.466 | 0.428 | 0.381 | 0.329 | 0.307 | 0.791 | 0.777 |
|  | Mis | NA | 0.130 | NA | 0.096 | 0.086 | 0.065 | 0.065 | 0.054 | 0.043 | 0.084 | 0.108 | 0.137 | 0.165 | 0.197 | 0.234 | 0.291 | 0.325 | 0.089 | 0.097 |
| Elbow | Freq | 0 | 3 | 3 | 21 | 89 | 118 | 194 | 206 | 267 | 58 | 16 | 4 | 0 | 2 | 1 | 2 | 0 | 984 | 984 |
|  | Sen | NA | 0.100 | 0.267 | 0.390 | 0.529 | 0.581 | 0.619 | 0.670 | 0.750 | 0.679 | 0.713 | 0.825 | NA | 0.950 | 1.000 | 1.000 | NA | 0.653 | 0.714 |
|  | PPV | NA | 1.000 | 0.917 | 1.000 | 0.950 | 0.931 | 0.930 | 0.918 | 0.918 | 0.705 | 0.617 | 0.527 | NA | 0.432 | 0.385 | 0.345 | NA | 0.905 | 0.777 |
|  | Mis | NA | 0.130 | 0.111 | 0.088 | 0.072 | 0.067 | 0.063 | 0.058 | 0.047 | 0.088 | 0.107 | 0.134 | NA | 0.188 | 0.232 | 0.275 | NA | 0.063 | 0.097 |
| MCS  -P | Freq | 0 | 1 | 1 | 15 | 52 | 87 | 159 | 177 | 275 | 75 | 59 | 25 | 19 | 9 | 13 | 11 | 6 | 984 | 984 |
|  | Sen | NA | 0.100 | 0.300 | 0.367 | 0.510 | 0.598 | 0.627 | 0.699 | 0.765 | 0.725 | 0.742 | 0.808 | 0.858 | 0.944 | 0.977 | 0.945 | 0.950 | 0.702 | 0.714 |
|  | PPV | NA | 1.000 | 1.000 | 0.983 | 0.962 | 0.932 | 0.928 | 0.919 | 0.932 | 0.744 | 0.612 | 0.519 | 0.456 | 0.414 | 0.371 | 0.329 | 0.286 | 0.856 | 0.777 |
|  | Mis | NA | 0.130 | 0.101 | 0.093 | 0.074 | 0.065 | 0.062 | 0.054 | 0.043 | 0.077 | 0.107 | 0.137 | 0.169 | 0.203 | 0.244 | 0.289 | 0.353 | 0.073 | 0.097 |
| MCHS  -P | Freq | 0 | 1 | 5 | 23 | 68 | 97 | 153 | 172 | 260 | 66 | 54 | 25 | 20 | 10 | 13 | 11 | 6 | 984 | 984 |
|  | Sen | NA | 0.100 | 0.320 | 0.413 | 0.521 | 0.608 | 0.626 | 0.701 | 0.766 | 0.708 | 0.735 | 0.812 | 0.865 | 0.950 | 0.977 | 0.945 | 0.950 | 0.695 | 0.714 |
|  | PPV | NA | 1.000 | 0.950 | 0.991 | 0.964 | 0.944 | 0.931 | 0.920 | 0.929 | 0.734 | 0.607 | 0.528 | 0.472 | 0.413 | 0.371 | 0.329 | 0.286 | 0.860 | 0.777 |
|  | Mis | NA | 0.130 | 0.101 | 0.086 | 0.072 | 0.062 | 0.062 | 0.054 | 0.044 | 0.081 | 0.109 | 0.134 | 0.163 | 0.204 | 0.244 | 0.289 | 0.353 | 0.073 | 0.097 |

^a^Freq: frequency. ^b^Sen: sensitivity. ^c^PPV: positive predictive value. ^d^Mis: misclassification.

Table A33. Ordinal model: Simulation results for true cluster model (C) with an alternative hypothesis (3) using elliptical windows.

|  |  | Maximum reported cluster size (MRCS) | | | | | | | | | | | | | | | | | | Default  Setting |
| --- | --- | --- | --- | --- | --- | --- | --- | --- | --- | --- | --- | --- | --- | --- | --- | --- | --- | --- | --- | --- |
|  |  | 1% | 2% | 3% | 4% | 5% | 6% | 8% | 10% | 12% | 15% | 20% | 25% | 30% | 35% | 40% | 45% | 50% | Overall |  |
| SCIC_1_ | Freq^a^ | 0 | 5 | 3 | 13 | 37 | 44 | 146 | 207 | 337 | 82 | 57 | 23 | 14 | 7 | 8 | 2 | 2 | 987 | 987 |
|  | Sen^b^ | NA | 0.120 | 0.167 | 0.300 | 0.332 | 0.436 | 0.541 | 0.647 | 0.728 | 0.685 | 0.742 | 0.809 | 0.807 | 0.929 | 1.000 | 0.950 | 0.850 | 0.650 | 0.718 |
|  | PPV^c^ | NA | 1.000 | 1.000 | 0.974 | 0.962 | 0.929 | 0.956 | 0.950 | 0.938 | 0.724 | 0.643 | 0.550 | 0.612 | 0.449 | 0.394 | 0.324 | 0.661 | 0.886 | 0.774 |
|  | Mis^d^ | NA | 0.128 | 0.121 | 0.103 | 0.099 | 0.087 | 0.070 | 0.056 | 0.047 | 0.086 | 0.100 | 0.128 | 0.137 | 0.184 | 0.225 | 0.297 | 0.174 | 0.070 | 0.098 |
| SCIC_2_ | Freq | 0 | 3 | 4 | 19 | 40 | 75 | 165 | 222 | 315 | 61 | 43 | 17 | 9 | 6 | 5 | 2 | 1 | 987 | 987 |
|  | Sen | NA | 0.100 | 0.250 | 0.358 | 0.383 | 0.516 | 0.596 | 0.676 | 0.752 | 0.707 | 0.747 | 0.806 | 0.844 | 0.917 | 1.000 | 0.950 | 1.000 | 0.666 | 0.718 |
|  | PPV | NA | 1.000 | 1.000 | 0.932 | 0.956 | 0.939 | 0.938 | 0.939 | 0.931 | 0.723 | 0.627 | 0.520 | 0.513 | 0.460 | 0.392 | 0.324 | 0.323 | 0.892 | 0.774 |
|  | Mis | NA | 0.130 | 0.109 | 0.097 | 0.092 | 0.076 | 0.065 | 0.054 | 0.046 | 0.084 | 0.103 | 0.136 | 0.159 | 0.176 | 0.226 | 0.297 | 0.304 | 0.066 | 0.098 |
| Gini | Freq | 0 | 0 | 0 | 10 | 28 | 59 | 109 | 127 | 304 | 68 | 74 | 53 | 51 | 33 | 40 | 20 | 11 | 987 | 987 |
|  | Sen | NA | NA | NA | 0.400 | 0.439 | 0.575 | 0.638 | 0.713 | 0.754 | 0.721 | 0.741 | 0.811 | 0.859 | 0.939 | 0.975 | 0.955 | 0.964 | 0.739 | 0.718 |
|  | PPV | NA | NA | NA | 0.980 | 0.978 | 0.946 | 0.931 | 0.917 | 0.927 | 0.747 | 0.610 | 0.533 | 0.452 | 0.422 | 0.372 | 0.326 | 0.298 | 0.789 | 0.774 |
|  | Mis | NA | NA | NA | 0.088 | 0.083 | 0.067 | 0.061 | 0.052 | 0.046 | 0.078 | 0.107 | 0.132 | 0.172 | 0.198 | 0.243 | 0.294 | 0.335 | 0.090 | 0.098 |
| Elbow | Freq | 0 | 1 | 4 | 39 | 92 | 130 | 181 | 204 | 258 | 51 | 14 | 6 | 5 | 0 | 2 | 0 | 0 | 987 | 987 |
|  | Sen | NA | 0.100 | 0.250 | 0.418 | 0.505 | 0.578 | 0.599 | 0.680 | 0.745 | 0.694 | 0.771 | 0.867 | 0.880 | NA | 1.000 | NA | NA | 0.645 | 0.718 |
|  | PPV | NA | 1.000 | 1.000 | 0.953 | 0.953 | 0.936 | 0.921 | 0.931 | 0.921 | 0.717 | 0.606 | 0.530 | 0.459 | NA | 0.408 | NA | NA | 0.909 | 0.774 |
|  | Mis | NA | 0.130 | 0.109 | 0.087 | 0.076 | 0.067 | 0.067 | 0.055 | 0.048 | 0.086 | 0.108 | 0.130 | 0.168 | NA | 0.210 | NA | NA | 0.064 | 0.098 |
| MCS  -P | Freq | 0 | 0 | 2 | 21 | 52 | 105 | 126 | 168 | 296 | 69 | 53 | 33 | 19 | 11 | 13 | 14 | 5 | 987 | 987 |
|  | Sen | NA | NA | 0.350 | 0.405 | 0.504 | 0.594 | 0.636 | 0.708 | 0.760 | 0.716 | 0.747 | 0.782 | 0.868 | 0.927 | 0.969 | 0.950 | 0.980 | 0.703 | 0.718 |
|  | PPV | NA | NA | 1.000 | 0.953 | 0.971 | 0.943 | 0.933 | 0.925 | 0.925 | 0.734 | 0.627 | 0.520 | 0.451 | 0.421 | 0.374 | 0.324 | 0.309 | 0.855 | 0.774 |
|  | Mis | NA | NA | 0.094 | 0.089 | 0.074 | 0.065 | 0.061 | 0.052 | 0.045 | 0.080 | 0.103 | 0.137 | 0.172 | 0.196 | 0.241 | 0.295 | 0.322 | 0.073 | 0.098 |
| MCHS  -P | Freq | 0 | 0 | 0 | 23 | 61 | 86 | 121 | 168 | 294 | 69 | 57 | 37 | 25 | 12 | 15 | 14 | 5 | 987 | 987 |
|  | Sen | NA | NA | NA | 0.370 | 0.495 | 0.553 | 0.616 | 0.693 | 0.737 | 0.703 | 0.751 | 0.795 | 0.868 | 0.933 | 0.967 | 0.950 | 0.980 | 0.689 | 0.718 |
|  | PPV | NA | NA | NA | 0.968 | 0.953 | 0.925 | 0.941 | 0.927 | 0.926 | 0.734 | 0.633 | 0.521 | 0.474 | 0.420 | 0.417 | 0.324 | 0.309 | 0.848 | 0.774 |
|  | Mis | NA | NA | NA | 0.093 | 0.077 | 0.072 | 0.063 | 0.054 | 0.048 | 0.081 | 0.102 | 0.136 | 0.167 | 0.197 | 0.224 | 0.295 | 0.322 | 0.077 | 0.098 |

^a^Freq: frequency. ^b^Sen: sensitivity. ^c^PPV: positive predictive value. ^d^Mis: misclassification.

Table A34. Ordinal model: Simulation results for true cluster model (C) with an alternative hypothesis (4) using elliptical windows.

|  |  | Maximum reported cluster size (MRCS) | | | | | | | | | | | | | | | | | | Default  Setting |
| --- | --- | --- | --- | --- | --- | --- | --- | --- | --- | --- | --- | --- | --- | --- | --- | --- | --- | --- | --- | --- |
|  |  | 1% | 2% | 3% | 4% | 5% | 6% | 8% | 10% | 12% | 15% | 20% | 25% | 30% | 35% | 40% | 45% | 50% | Overall |  |
| SCIC_1_ | Freq^a^ | 0 | 4 | 9 | 15 | 29 | 32 | 166 | 203 | 307 | 84 | 59 | 26 | 17 | 12 | 12 | 8 | 0 | 983 | 983 |
|  | Sen^b^ | NA | 0.100 | 0.200 | 0.247 | 0.310 | 0.413 | 0.523 | 0.648 | 0.716 | 0.679 | 0.734 | 0.800 | 0.865 | 0.875 | 0.950 | 0.950 | NA | 0.643 | 0.719 |
|  | PPV^c^ | NA | 1.000 | 0.917 | 0.956 | 0.954 | 0.958 | 0.965 | 0.937 | 0.950 | 0.737 | 0.639 | 0.590 | 0.499 | 0.506 | 0.366 | 0.423 | NA | 0.879 | 0.746 |
|  | Mis^d^ | NA | 0.130 | 0.119 | 0.111 | 0.103 | 0.088 | 0.072 | 0.057 | 0.047 | 0.083 | 0.101 | 0.119 | 0.157 | 0.181 | 0.246 | 0.250 | NA | 0.074 | 0.108 |
| SCIC_2_ | Freq | 0 | 3 | 9 | 19 | 38 | 52 | 187 | 211 | 300 | 67 | 44 | 20 | 11 | 7 | 10 | 5 | 0 | 983 | 983 |
|  | Sen | NA | 0.100 | 0.233 | 0.300 | 0.408 | 0.519 | 0.571 | 0.667 | 0.739 | 0.704 | 0.732 | 0.805 | 0.873 | 0.914 | 0.960 | 0.980 | NA | 0.657 | 0.719 |
|  | PPV | NA | 1.000 | 0.917 | 0.947 | 0.957 | 0.938 | 0.956 | 0.934 | 0.951 | 0.741 | 0.626 | 0.537 | 0.465 | 0.410 | 0.368 | 0.343 | NA | 0.892 | 0.746 |
|  | Mis | NA | 0.130 | 0.114 | 0.104 | 0.089 | 0.075 | 0.066 | 0.055 | 0.044 | 0.079 | 0.103 | 0.130 | 0.166 | 0.205 | 0.245 | 0.275 | NA | 0.069 | 0.108 |
| Gini | Freq | 0 | 1 | 4 | 10 | 22 | 61 | 113 | 135 | 241 | 61 | 81 | 74 | 51 | 44 | 48 | 24 | 13 | 983 | 983 |
|  | Sen | NA | 0.100 | 0.225 | 0.360 | 0.441 | 0.600 | 0.615 | 0.690 | 0.742 | 0.703 | 0.733 | 0.809 | 0.869 | 0.941 | 0.958 | 0.967 | 0.977 | 0.735 | 0.719 |
|  | PPV | NA | 1.000 | 1.000 | 1.000 | 0.975 | 0.954 | 0.949 | 0.924 | 0.945 | 0.735 | 0.613 | 0.534 | 0.465 | 0.422 | 0.371 | 0.329 | 0.302 | 0.773 | 0.746 |
|  | Mis | NA | 0.130 | 0.112 | 0.093 | 0.083 | 0.062 | 0.061 | 0.054 | 0.044 | 0.081 | 0.107 | 0.130 | 0.165 | 0.199 | 0.242 | 0.292 | 0.331 | 0.097 | 0.108 |
| Elbow | Freq | 0 | 3 | 8 | 40 | 70 | 116 | 212 | 233 | 229 | 48 | 14 | 4 | 0 | 2 | 3 | 1 | 0 | 983 | 983 |
|  | Sen | NA | 0.167 | 0.250 | 0.403 | 0.489 | 0.599 | 0.584 | 0.666 | 0.729 | 0.696 | 0.714 | 0.750 | NA | 0.900 | 0.967 | 1.000 | NA | 0.631 | 0.719 |
|  | PPV | NA | 1.000 | 0.875 | 0.948 | 0.962 | 0.949 | 0.936 | 0.918 | 0.941 | 0.742 | 0.605 | 0.523 | NA | 0.400 | 0.364 | 0.370 | NA | 0.917 | 0.746 |
|  | Mis | NA | 0.121 | 0.114 | 0.090 | 0.077 | 0.063 | 0.067 | 0.058 | 0.047 | 0.080 | 0.111 | 0.138 | NA | 0.210 | 0.251 | 0.246 | NA | 0.064 | 0.108 |
| MCS  -P | Freq | 0 | 1 | 4 | 23 | 42 | 91 | 151 | 177 | 254 | 68 | 56 | 39 | 20 | 13 | 26 | 12 | 6 | 983 | 983 |
|  | Sen | NA | 0.100 | 0.225 | 0.391 | 0.495 | 0.614 | 0.613 | 0.694 | 0.757 | 0.706 | 0.732 | 0.790 | 0.875 | 0.908 | 0.965 | 0.967 | 0.983 | 0.698 | 0.719 |
|  | PPV | NA | 1.000 | 1.000 | 0.972 | 0.970 | 0.957 | 0.942 | 0.930 | 0.946 | 0.749 | 0.611 | 0.521 | 0.473 | 0.402 | 0.372 | 0.333 | 0.289 | 0.852 | 0.746 |
|  | Mis | NA | 0.130 | 0.112 | 0.090 | 0.076 | 0.060 | 0.062 | 0.053 | 0.042 | 0.078 | 0.107 | 0.137 | 0.162 | 0.210 | 0.242 | 0.286 | 0.353 | 0.077 | 0.108 |
| MCHS  -P | Freq | 0 | 3 | 8 | 35 | 53 | 96 | 153 | 169 | 238 | 67 | 51 | 36 | 19 | 14 | 25 | 12 | 4 | 983 | 983 |
|  | Sen | NA | 0.200 | 0.275 | 0.406 | 0.506 | 0.604 | 0.614 | 0.695 | 0.772 | 0.706 | 0.729 | 0.789 | 0.884 | 0.914 | 0.964 | 0.967 | 0.975 | 0.691 | 0.719 |
|  | PPV | NA | 1.000 | 0.969 | 0.971 | 0.965 | 0.950 | 0.934 | 0.925 | 0.946 | 0.745 | 0.609 | 0.521 | 0.473 | 0.433 | 0.371 | 0.333 | 0.287 | 0.855 | 0.746 |
|  | Mis | NA | 0.116 | 0.107 | 0.088 | 0.075 | 0.062 | 0.063 | 0.053 | 0.040 | 0.079 | 0.109 | 0.137 | 0.162 | 0.197 | 0.242 | 0.286 | 0.355 | 0.076 | 0.108 |

^a^Freq: frequency. ^b^Sen: sensitivity. ^c^PPV: positive predictive value. ^d^Mis: misclassification.

Table A35. Ordinal model: Simulation results for true cluster model (D) with an alternative hypothesis (1) using elliptical windows.

|  |  | Maximum reported cluster size (MRCS) | | | | | | | | | | | | | | | | | | Default  Setting |
| --- | --- | --- | --- | --- | --- | --- | --- | --- | --- | --- | --- | --- | --- | --- | --- | --- | --- | --- | --- | --- |
|  |  | 1% | 2% | 3% | 4% | 5% | 6% | 8% | 10% | 12% | 15% | 20% | 25% | 30% | 35% | 40% | 45% | 50% | Overall |  |
| SCIC_1_ | Freq^a^ | 0 | 0 | 1 | 5 | 18 | 29 | 417 | 149 | 75 | 25 | 52 | 140 | 55 | 23 | 3 | 6 | 1 | 999 | 999 |
|  | Sen^b^ | NA | NA | 0.300 | 0.240 | 0.394 | 0.497 | 0.847 | 0.903 | 0.901 | 0.836 | 0.817 | 0.934 | 0.933 | 0.957 | 0.833 | 0.967 | 0.500 | 0.856 | 0.873 |
|  | PPV^c^ | NA | NA | 1.000 | 1.000 | 0.970 | 0.968 | 0.984 | 0.881 | 0.784 | 0.724 | 0.687 | 0.687 | 0.595 | 0.511 | 0.590 | 0.431 | 0.714 | 0.852 | 0.770 |
|  | Mis^d^ | NA | NA | 0.101 | 0.110 | 0.089 | 0.075 | 0.024 | 0.031 | 0.050 | 0.072 | 0.084 | 0.076 | 0.113 | 0.156 | 0.179 | 0.227 | 0.101 | 0.052 | 0.076 |
| SCIC_2_ | Freq | 0 | 0 | 1 | 5 | 13 | 29 | 520 | 220 | 108 | 32 | 22 | 32 | 12 | 3 | 0 | 2 | 0 | 999 | 999 |
|  | Sen | NA | NA | 0.300 | 0.340 | 0.523 | 0.559 | 0.867 | 0.923 | 0.929 | 0.966 | 0.923 | 0.963 | 0.992 | 1.000 | NA | 0.900 | NA | 0.879 | 0.873 |
|  | PPV | NA | NA | 1.000 | 0.900 | 0.972 | 0.968 | 0.983 | 0.883 | 0.784 | 0.711 | 0.742 | 0.670 | 0.621 | 0.486 | NA | 0.572 | NA | 0.908 | 0.770 |
|  | Mis | NA | NA | 0.101 | 0.101 | 0.071 | 0.066 | 0.022 | 0.029 | 0.047 | 0.063 | 0.063 | 0.078 | 0.097 | 0.155 | NA | 0.167 | NA | 0.034 | 0.076 |
| Gini | Freq | 0 | 0 | 0 | 0 | 1 | 6 | 369 | 171 | 121 | 56 | 41 | 62 | 100 | 46 | 14 | 6 | 6 | 999 | 999 |
|  | Sen | NA | NA | NA | NA | 0.600 | 0.383 | 0.859 | 0.929 | 0.930 | 0.966 | 0.946 | 0.961 | 0.990 | 0.993 | 0.993 | 0.967 | 0.967 | 0.915 | 0.873 |
|  | PPV | NA | NA | NA | NA | 1.000 | 0.913 | 0.991 | 0.881 | 0.783 | 0.708 | 0.708 | 0.668 | 0.570 | 0.507 | 0.392 | 0.429 | 0.302 | 0.819 | 0.770 |
|  | Mis | NA | NA | NA | NA | 0.058 | 0.099 | 0.022 | 0.028 | 0.047 | 0.064 | 0.070 | 0.081 | 0.115 | 0.152 | 0.226 | 0.229 | 0.329 | 0.056 | 0.076 |
| Elbow | Freq | 0 | 0 | 2 | 15 | 24 | 26 | 661 | 193 | 61 | 10 | 3 | 4 | 0 | 0 | 0 | 0 | 0 | 999 | 999 |
|  | Sen | NA | NA | 0.450 | 0.560 | 0.592 | 0.546 | 0.881 | 0.917 | 0.921 | 0.970 | 0.800 | 0.950 | NA | NA | NA | NA | NA | 0.870 | 0.873 |
|  | PPV | NA | NA | 1.000 | 0.959 | 0.909 | 0.959 | 0.981 | 0.870 | 0.768 | 0.693 | 0.585 | 0.585 | NA | NA | NA | NA | NA | 0.938 | 0.770 |
|  | Mis | NA | NA | 0.080 | 0.067 | 0.068 | 0.070 | 0.020 | 0.032 | 0.052 | 0.068 | 0.111 | 0.112 | NA | NA | NA | NA | NA | 0.028 | 0.076 |
| MCS  -P | Freq | 0 | 0 | 0 | 2 | 4 | 12 | 512 | 230 | 134 | 44 | 31 | 14 | 9 | 4 | 1 | 2 | 0 | 999 | 999 |
|  | Sen | NA | NA | NA | 0.450 | 0.525 | 0.483 | 0.871 | 0.931 | 0.934 | 0.973 | 0.952 | 0.936 | 0.944 | 1.000 | 1.000 | 0.900 | NA | 0.896 | 0.873 |
|  | PPV | NA | NA | NA | 0.875 | 0.873 | 0.956 | 0.986 | 0.884 | 0.785 | 0.710 | 0.723 | 0.747 | 0.621 | 0.497 | 0.417 | 0.572 | NA | 0.904 | 0.770 |
|  | Mis | NA | NA | NA | 0.087 | 0.080 | 0.080 | 0.021 | 0.028 | 0.047 | 0.063 | 0.068 | 0.068 | 0.103 | 0.149 | 0.203 | 0.167 | NA | 0.033 | 0.076 |
| MCHS  -P | Freq | 0 | 0 | 1 | 9 | 18 | 31 | 503 | 190 | 116 | 48 | 38 | 18 | 14 | 9 | 1 | 2 | 1 | 999 | 999 |
|  | Sen | NA | NA | 0.300 | 0.456 | 0.583 | 0.584 | 0.880 | 0.925 | 0.932 | 0.965 | 0.932 | 0.950 | 0.900 | 0.989 | 1.000 | 0.900 | 0.900 | 0.884 | 0.873 |
|  | PPV | NA | NA | 1.000 | 0.950 | 0.985 | 0.970 | 0.986 | 0.873 | 0.787 | 0.709 | 0.717 | 0.686 | 0.763 | 0.601 | 0.417 | 0.572 | 0.281 | 0.903 | 0.770 |
|  | Mis | NA | NA | 0.101 | 0.082 | 0.062 | 0.064 | 0.019 | 0.030 | 0.046 | 0.064 | 0.071 | 0.084 | 0.068 | 0.116 | 0.203 | 0.167 | 0.348 | 0.035 | 0.076 |

^a^Freq: frequency. ^b^Sen: sensitivity. ^c^PPV: positive predictive value. ^d^Mis: misclassification.

Table A36. Ordinal model: Simulation results for true cluster model (D) with an alternative hypothesis (2) using elliptical windows.

|  |  | Maximum reported cluster size (MRCS) | | | | | | | | | | | | | | | | | | Default  Setting |
| --- | --- | --- | --- | --- | --- | --- | --- | --- | --- | --- | --- | --- | --- | --- | --- | --- | --- | --- | --- | --- |
|  |  | 1% | 2% | 3% | 4% | 5% | 6% | 8% | 10% | 12% | 15% | 20% | 25% | 30% | 35% | 40% | 45% | 50% | Overall |  |
| SCIC_1_ | Freq^a^ | 0 | 1 | 3 | 12 | 39 | 48 | 380 | 123 | 64 | 39 | 41 | 131 | 73 | 25 | 8 | 5 | 0 | 992 | 992 |
|  | Sen^b^ | NA | 0.100 | 0.200 | 0.242 | 0.351 | 0.421 | 0.765 | 0.835 | 0.867 | 0.721 | 0.817 | 0.934 | 0.973 | 0.900 | 0.738 | 0.900 | NA | 0.781 | 0.834 |
|  | PPV^c^ | NA | 1.000 | 1.000 | 1.000 | 0.956 | 0.962 | 0.980 | 0.860 | 0.771 | 0.680 | 0.665 | 0.662 | 0.576 | 0.531 | 0.630 | 0.344 | NA | 0.836 | 0.750 |
|  | Mis^d^ | NA | 0.130 | 0.116 | 0.110 | 0.096 | 0.086 | 0.036 | 0.043 | 0.057 | 0.090 | 0.086 | 0.082 | 0.116 | 0.154 | 0.156 | 0.258 | NA | 0.066 | 0.088 |
| SCIC_2_ | Freq | 0 | 0 | 2 | 8 | 33 | 47 | 449 | 191 | 109 | 49 | 19 | 45 | 31 | 8 | 0 | 1 | 0 | 992 | 992 |
|  | Sen | NA | NA | 0.200 | 0.288 | 0.415 | 0.489 | 0.800 | 0.871 | 0.904 | 0.837 | 0.853 | 0.958 | 0.981 | 0.975 | NA | 1.000 | NA | 0.810 | 0.834 |
|  | PPV | NA | NA | 1.000 | 1.000 | 0.973 | 0.968 | 0.975 | 0.861 | 0.767 | 0.677 | 0.670 | 0.666 | 0.584 | 0.496 | NA | 0.370 | NA | 0.879 | 0.750 |
|  | Mis | NA | NA | 0.116 | 0.103 | 0.087 | 0.076 | 0.032 | 0.039 | 0.054 | 0.081 | 0.085 | 0.078 | 0.111 | 0.154 | NA | 0.246 | NA | 0.050 | 0.088 |
| Gini | Freq | 0 | 0 | 0 | 1 | 4 | 15 | 322 | 141 | 116 | 76 | 42 | 77 | 106 | 56 | 21 | 9 | 6 | 992 | 992 |
|  | Sen | NA | NA | NA | 0.200 | 0.350 | 0.407 | 0.771 | 0.864 | 0.915 | 0.886 | 0.893 | 0.943 | 0.988 | 0.982 | 0.971 | 0.978 | 0.983 | 0.863 | 0.834 |
|  | PPV | NA | NA | NA | 1.000 | 1.000 | 0.990 | 0.983 | 0.863 | 0.774 | 0.667 | 0.660 | 0.643 | 0.553 | 0.473 | 0.384 | 0.348 | 0.308 | 0.780 | 0.750 |
|  | Mis | NA | NA | NA | 0.116 | 0.094 | 0.087 | 0.035 | 0.039 | 0.051 | 0.080 | 0.088 | 0.088 | 0.121 | 0.165 | 0.231 | 0.271 | 0.324 | 0.073 | 0.088 |
| Elbow | Freq | 0 | 0 | 2 | 6 | 31 | 46 | 562 | 196 | 90 | 29 | 17 | 9 | 3 | 1 | 0 | 0 | 0 | 992 | 992 |
|  | Sen | NA | NA | 0.350 | 0.250 | 0.439 | 0.474 | 0.809 | 0.863 | 0.894 | 0.797 | 0.835 | 0.978 | 1.000 | 1.000 | NA | NA | NA | 0.798 | 0.8336 |
|  | PPV | NA | NA | 1.000 | 1.000 | 0.937 | 0.965 | 0.973 | 0.851 | 0.754 | 0.650 | 0.651 | 0.641 | 0.559 | 0.435 | NA | NA | NA | 0.908 | 0.7504 |
|  | Mis | NA | NA | 0.094 | 0.109 | 0.086 | 0.079 | 0.031 | 0.041 | 0.058 | 0.091 | 0.090 | 0.084 | 0.116 | 0.188 | NA | NA | NA | 0.044 | 0.0877 |
| MCS  -P | Freq | 0 | 0 | 0 | 2 | 10 | 22 | 462 | 193 | 134 | 68 | 22 | 33 | 31 | 9 | 3 | 2 | 1 | 992 | 992 |
|  | Sen | NA | NA | NA | 0.350 | 0.430 | 0.459 | 0.805 | 0.890 | 0.915 | 0.865 | 0.882 | 0.936 | 0.965 | 0.956 | 1.000 | 0.950 | 1.000 | 0.841 | 0.834 |
|  | PPV | NA | NA | NA | 1.000 | 1.000 | 0.987 | 0.978 | 0.868 | 0.768 | 0.666 | 0.632 | 0.646 | 0.540 | 0.473 | 0.376 | 0.334 | 0.323 | 0.866 | 0.750 |
|  | Mis | NA | NA | NA | 0.094 | 0.083 | 0.080 | 0.031 | 0.035 | 0.053 | 0.082 | 0.093 | 0.087 | 0.131 | 0.167 | 0.242 | 0.283 | 0.304 | 0.049 | 0.088 |
| MCHS  -P | Freq | 0 | 0 | 1 | 3 | 18 | 35 | 450 | 184 | 113 | 74 | 27 | 38 | 34 | 9 | 3 | 2 | 1 | 992 | 992 |
|  | Sen | NA | NA | 0.400 | 0.367 | 0.483 | 0.517 | 0.810 | 0.880 | 0.909 | 0.874 | 0.893 | 0.934 | 0.968 | 0.944 | 1.000 | 0.950 | 1.000 | 0.835 | 0.834 |
|  | PPV | NA | NA | 1.000 | 0.933 | 0.981 | 0.973 | 0.979 | 0.861 | 0.775 | 0.668 | 0.676 | 0.623 | 0.560 | 0.453 | 0.376 | 0.334 | 0.323 | 0.866 | 0.750 |
|  | Mis | NA | NA | 0.087 | 0.097 | 0.076 | 0.072 | 0.030 | 0.038 | 0.051 | 0.081 | 0.083 | 0.096 | 0.124 | 0.176 | 0.242 | 0.283 | 0.304 | 0.050 | 0.088 |

^a^Freq: frequency. ^b^Sen: sensitivity. ^c^PPV: positive predictive value. ^d^Mis: misclassification.

Table A37. Ordinal model: Simulation results for true cluster model (D) with an alternative hypothesis (3) using elliptical windows.

|  |  | Maximum reported cluster size (MRCS) | | | | | | | | | | | | | | | | | | Default  Setting |
| --- | --- | --- | --- | --- | --- | --- | --- | --- | --- | --- | --- | --- | --- | --- | --- | --- | --- | --- | --- | --- |
|  |  | 1% | 2% | 3% | 4% | 5% | 6% | 8% | 10% | 12% | 15% | 20% | 25% | 30% | 35% | 40% | 45% | 50% | Overall |  |
| SCIC_1_ | Freq^a^ | 0 | 1 | 10 | 7 | 27 | 44 | 354 | 90 | 63 | 34 | 71 | 172 | 85 | 20 | 8 | 0 | 2 | 988 | 988 |
|  | Sen^b^ | NA | 0.100 | 0.180 | 0.271 | 0.333 | 0.459 | 0.729 | 0.778 | 0.776 | 0.709 | 0.782 | 0.940 | 0.944 | 0.910 | 0.913 | NA | 1.000 | 0.768 | 0.814 |
|  | PPV^c^ | NA | 1.000 | 1.000 | 0.952 | 1.000 | 0.957 | 0.977 | 0.859 | 0.756 | 0.707 | 0.669 | 0.660 | 0.573 | 0.550 | 0.473 | NA | 0.323 | 0.817 | 0.737 |
|  | Mis^d^ | NA | 0.130 | 0.119 | 0.108 | 0.097 | 0.082 | 0.042 | 0.050 | 0.067 | 0.087 | 0.090 | 0.082 | 0.118 | 0.147 | 0.199 | NA | 0.304 | 0.071 | 0.093 |
| SCIC_2_ | Freq | 0 | 1 | 8 | 6 | 23 | 50 | 465 | 150 | 107 | 42 | 32 | 57 | 34 | 8 | 5 | 0 | 0 | 988 | 988 |
|  | Sen | NA | 0.100 | 0.200 | 0.417 | 0.426 | 0.512 | 0.779 | 0.863 | 0.861 | 0.826 | 0.800 | 0.947 | 0.968 | 0.938 | 0.960 | NA | NA | 0.792 | 0.814 |
|  | PPV | NA | 1.000 | 1.000 | 0.943 | 0.986 | 0.953 | 0.979 | 0.861 | 0.771 | 0.683 | 0.676 | 0.647 | 0.551 | 0.535 | 0.384 | NA | NA | 0.874 | 0.737 |
|  | Mis | NA | 0.130 | 0.116 | 0.089 | 0.084 | 0.074 | 0.035 | 0.040 | 0.056 | 0.080 | 0.089 | 0.085 | 0.126 | 0.147 | 0.229 | NA | NA | 0.054 | 0.093 |
| Gini | Freq | 0 | 1 | 3 | 0 | 5 | 17 | 325 | 140 | 128 | 56 | 43 | 80 | 108 | 44 | 27 | 7 | 4 | 988 | 988 |
|  | Sen | NA | 0.100 | 0.267 | NA | 0.300 | 0.453 | 0.751 | 0.845 | 0.866 | 0.895 | 0.853 | 0.933 | 0.984 | 0.982 | 0.985 | 0.986 | 0.975 | 0.842 | 0.814 |
|  | PPV | NA | 1.000 | 1.000 | NA | 1.000 | 0.992 | 0.983 | 0.861 | 0.773 | 0.687 | 0.655 | 0.633 | 0.556 | 0.473 | 0.415 | 0.386 | 0.385 | 0.788 | 0.737 |
|  | Mis | NA | 0.130 | 0.106 | NA | 0.101 | 0.080 | 0.038 | 0.042 | 0.055 | 0.074 | 0.089 | 0.090 | 0.122 | 0.167 | 0.210 | 0.251 | 0.264 | 0.073 | 0.093 |
| Elbow | Freq | 0 | 1 | 11 | 7 | 21 | 51 | 594 | 158 | 92 | 25 | 16 | 8 | 1 | 2 | 1 | 0 | 0 | 988 | 988 |
|  | Sen | NA | 0.100 | 0.236 | 0.443 | 0.395 | 0.522 | 0.794 | 0.845 | 0.834 | 0.816 | 0.750 | 0.925 | 1.000 | 1.000 | 1.000 | NA | NA | 0.775 | 0.814 |
|  | PPV | NA | 1.000 | 1.000 | 0.951 | 0.960 | 0.954 | 0.976 | 0.847 | 0.753 | 0.653 | 0.609 | 0.623 | 0.556 | 0.500 | 0.400 | NA | NA | 0.914 | 0.737 |
|  | Mis | NA | 0.130 | 0.111 | 0.085 | 0.090 | 0.073 | 0.033 | 0.044 | 0.062 | 0.089 | 0.109 | 0.094 | 0.116 | 0.145 | 0.217 | NA | NA | 0.046 | 0.093 |
| MCS  -P | Freq | 0 | 1 | 7 | 3 | 9 | 24 | 450 | 185 | 145 | 58 | 29 | 21 | 28 | 18 | 9 | 1 | 0 | 988 | 988 |
|  | Sen | NA | 0.100 | 0.271 | 0.367 | 0.367 | 0.479 | 0.793 | 0.863 | 0.882 | 0.895 | 0.810 | 0.919 | 0.979 | 0.967 | 0.956 | 1.000 | NA | 0.821 | 0.814 |
|  | PPV | NA | 1.000 | 1.000 | 1.000 | 1.000 | 0.966 | 0.982 | 0.864 | 0.774 | 0.692 | 0.643 | 0.653 | 0.557 | 0.499 | 0.379 | 0.333 | NA | 0.868 | 0.737 |
|  | Mis | NA | 0.130 | 0.106 | 0.092 | 0.092 | 0.078 | 0.032 | 0.039 | 0.054 | 0.073 | 0.096 | 0.087 | 0.124 | 0.156 | 0.233 | 0.290 | NA | 0.051 | 0.093 |
| MCHS  -P | Freq | 0 | 1 | 8 | 8 | 33 | 52 | 399 | 155 | 112 | 55 | 36 | 49 | 43 | 24 | 11 | 2 | 0 | 988 | 988 |
|  | Sen | NA | 0.100 | 0.250 | 0.300 | 0.500 | 0.546 | 0.780 | 0.856 | 0.861 | 0.873 | 0.822 | 0.902 | 0.972 | 0.933 | 0.964 | 1.000 | NA | 0.798 | 0.814 |
|  | PPV | NA | 1.000 | 1.000 | 1.000 | 0.984 | 0.973 | 0.979 | 0.859 | 0.774 | 0.682 | 0.636 | 0.669 | 0.560 | 0.525 | 0.382 | 0.500 | NA | 0.856 | 0.737 |
|  | Mis | NA | 0.130 | 0.109 | 0.101 | 0.074 | 0.068 | 0.034 | 0.041 | 0.056 | 0.078 | 0.097 | 0.085 | 0.122 | 0.150 | 0.232 | 0.181 | NA | 0.058 | 0.093 |

^a^Freq: frequency. ^b^Sen: sensitivity. ^c^PPV: positive predictive value. ^d^Mis: misclassification.

Table A38. Ordinal model: Simulation results for true cluster model (D) with an alternative hypothesis (4) using elliptical windows.

|  |  | Maximum reported cluster size (MRCS) | | | | | | | | | | | | | | | | | | Default  Setting |
| --- | --- | --- | --- | --- | --- | --- | --- | --- | --- | --- | --- | --- | --- | --- | --- | --- | --- | --- | --- | --- |
|  |  | 1% | 2% | 3% | 4% | 5% | 6% | 8% | 10% | 12% | 15% | 20% | 25% | 30% | 35% | 40% | 45% | 50% | Overall |  |
| SCIC_1_ | Freq^a^ | 0 | 2 | 7 | 20 | 44 | 34 | 317 | 45 | 35 | 31 | 103 | 201 | 111 | 24 | 11 | 3 | 0 | 988 | 988 |
|  | Sen^b^ | NA | 0.100 | 0.200 | 0.240 | 0.350 | 0.412 | 0.701 | 0.764 | 0.737 | 0.703 | 0.779 | 0.941 | 0.962 | 0.950 | 0.900 | 0.833 | NA | 0.760 | 0.807 |
|  | PPV^c^ | NA | 1.000 | 1.000 | 1.000 | 0.985 | 0.980 | 0.981 | 0.832 | 0.747 | 0.715 | 0.685 | 0.667 | 0.571 | 0.505 | 0.447 | 0.568 | NA | 0.799 | 0.706 |
|  | Mis^d^ | NA | 0.130 | 0.116 | 0.110 | 0.095 | 0.087 | 0.045 | 0.055 | 0.074 | 0.084 | 0.087 | 0.080 | 0.117 | 0.156 | 0.206 | 0.203 | NA | 0.078 | 0.105 |
| SCIC_2_ | Freq | 0 | 2 | 3 | 21 | 39 | 48 | 478 | 100 | 73 | 35 | 39 | 91 | 41 | 12 | 5 | 1 | 0 | 988 | 988 |
|  | Sen | NA | 0.100 | 0.200 | 0.267 | 0.408 | 0.473 | 0.772 | 0.840 | 0.848 | 0.803 | 0.821 | 0.959 | 0.978 | 0.975 | 0.980 | 1.000 | NA | 0.774 | 0.807 |
|  | PPV | NA | 1.000 | 1.000 | 1.000 | 0.980 | 0.971 | 0.980 | 0.853 | 0.771 | 0.714 | 0.683 | 0.671 | 0.566 | 0.523 | 0.390 | 0.370 | NA | 0.876 | 0.706 |
|  | Mis | NA | 0.130 | 0.116 | 0.106 | 0.087 | 0.079 | 0.036 | 0.043 | 0.059 | 0.075 | 0.083 | 0.077 | 0.117 | 0.149 | 0.226 | 0.246 | NA | 0.057 | 0.105 |
| Gini | Freq | 0 | 1 | 1 | 0 | 12 | 11 | 349 | 118 | 107 | 57 | 40 | 73 | 123 | 52 | 30 | 10 | 4 | 988 | 988 |
|  | Sen | NA | 0.100 | 0.200 | NA | 0.317 | 0.436 | 0.726 | 0.855 | 0.888 | 0.904 | 0.850 | 0.922 | 0.979 | 0.990 | 0.983 | 0.990 | 0.900 | 0.836 | 0.807 |
|  | PPV | NA | 1.000 | 1.000 | NA | 1.000 | 0.955 | 0.988 | 0.860 | 0.777 | 0.688 | 0.679 | 0.623 | 0.558 | 0.489 | 0.404 | 0.353 | 0.285 | 0.786 | 0.706 |
|  | Mis | NA | 0.130 | 0.116 | NA | 0.099 | 0.084 | 0.041 | 0.041 | 0.053 | 0.074 | 0.084 | 0.094 | 0.121 | 0.159 | 0.217 | 0.265 | 0.341 | 0.076 | 0.105 |
| Elbow | Freq | 0 | 2 | 10 | 12 | 43 | 44 | 631 | 123 | 67 | 14 | 17 | 15 | 7 | 2 | 1 | 0 | 0 | 988 | 988 |
|  | Sen | NA | 0.100 | 0.260 | 0.317 | 0.409 | 0.500 | 0.788 | 0.830 | 0.815 | 0.750 | 0.782 | 0.960 | 0.971 | 0.950 | 1.000 | NA | NA | 0.757 | 0.807 |
|  | PPV | NA | 1.000 | 0.980 | 1.000 | 0.969 | 0.971 | 0.975 | 0.841 | 0.749 | 0.693 | 0.634 | 0.660 | 0.523 | 0.434 | 0.385 | NA | NA | 0.923 | 0.706 |
|  | Mis | NA | 0.130 | 0.109 | 0.099 | 0.088 | 0.075 | 0.034 | 0.047 | 0.066 | 0.085 | 0.097 | 0.078 | 0.137 | 0.188 | 0.232 | NA | NA | 0.047 | 0.105 |
| MCS  -P | Freq | 0 | 1 | 3 | 2 | 20 | 23 | 481 | 154 | 118 | 59 | 27 | 41 | 41 | 8 | 6 | 2 | 2 | 988 | 988 |
|  | Sen | NA | 0.100 | 0.233 | 0.200 | 0.390 | 0.491 | 0.770 | 0.872 | 0.893 | 0.880 | 0.830 | 0.924 | 0.980 | 0.963 | 0.967 | 1.000 | 1.000 | 0.810 | 0.807 |
|  | PPV | NA | 1.000 | 1.000 | 1.000 | 0.994 | 0.971 | 0.982 | 0.858 | 0.776 | 0.688 | 0.673 | 0.643 | 0.561 | 0.450 | 0.385 | 0.364 | 0.308 | 0.870 | 0.706 |
|  | Mis | NA | 0.130 | 0.111 | 0.116 | 0.089 | 0.076 | 0.036 | 0.039 | 0.053 | 0.076 | 0.086 | 0.088 | 0.122 | 0.178 | 0.229 | 0.254 | 0.326 | 0.054 | 0.105 |
| MCHS  -P | Freq | 0 | 1 | 7 | 4 | 18 | 14 | 452 | 148 | 131 | 70 | 35 | 40 | 47 | 10 | 7 | 2 | 2 | 988 | 988 |
|  | Sen | NA | 0.100 | 0.271 | 0.300 | 0.372 | 0.457 | 0.767 | 0.861 | 0.903 | 0.890 | 0.863 | 0.928 | 0.974 | 0.960 | 0.971 | 1.000 | 1.000 | 0.814 | 0.807 |
|  | PPV | NA | 1.000 | 1.000 | 1.000 | 0.993 | 0.964 | 0.985 | 0.856 | 0.782 | 0.695 | 0.679 | 0.637 | 0.585 | 0.525 | 0.425 | 0.364 | 0.308 | 0.863 | 0.706 |
|  | Mis | NA | 0.130 | 0.106 | 0.101 | 0.092 | 0.081 | 0.036 | 0.041 | 0.051 | 0.073 | 0.084 | 0.089 | 0.115 | 0.151 | 0.207 | 0.254 | 0.326 | 0.055 | 0.105 |

^a^Freq: frequency. ^b^Sen: sensitivity. ^c^PPV: positive predictive value. ^d^Mis: misclassification.

Table A39. Ordinal model: Simulation results for true cluster model (D) with an alternative hypothesis (5) using elliptical windows.

|  |  | Maximum reported cluster size (MRCS) | | | | | | | | | | | | | | | | | | Default  Setting |
| --- | --- | --- | --- | --- | --- | --- | --- | --- | --- | --- | --- | --- | --- | --- | --- | --- | --- | --- | --- | --- |
|  |  | 1% | 2% | 3% | 4% | 5% | 6% | 8% | 10% | 12% | 15% | 20% | 25% | 30% | 35% | 40% | 45% | 50% | Overall |  |
| SCIC_1_ | Freq^a^ | 1 | 2 | 3 | 4 | 30 | 40 | 419 | 163 | 88 | 29 | 44 | 92 | 53 | 20 | 3 | 6 | 0 | 997 | 997 |
|  | Sen^b^ | 0.100 | 0.100 | 0.167 | 0.175 | 0.363 | 0.473 | 0.798 | 0.909 | 0.889 | 0.793 | 0.795 | 0.928 | 0.915 | 0.895 | 0.833 | 0.833 | NA | 0.812 | 0.847 |
|  | PPV^c^ | 1.000 | 1.000 | 0.833 | 0.875 | 0.956 | 0.982 | 0.983 | 0.883 | 0.779 | 0.689 | 0.648 | 0.676 | 0.584 | 0.568 | 0.600 | 0.526 | NA | 0.862 | 0.794 |
|  | Mis^d^ | 0.130 | 0.130 | 0.126 | 0.123 | 0.095 | 0.078 | 0.031 | 0.030 | 0.052 | 0.081 | 0.096 | 0.080 | 0.121 | 0.147 | 0.169 | 0.200 | NA | 0.055 | 0.073 |
| SCIC_2_ | Freq | 0 | 2 | 2 | 5 | 21 | 30 | 508 | 221 | 113 | 40 | 18 | 22 | 11 | 4 | 0 | 0 | 0 | 997 | 997 |
|  | Sen | NA | 0.100 | 0.150 | 0.220 | 0.452 | 0.527 | 0.828 | 0.917 | 0.904 | 0.888 | 0.878 | 0.964 | 0.955 | 0.975 | NA | NA | NA | 0.842 | 0.847 |
|  | PPV | NA | 1.000 | 0.750 | 0.900 | 0.936 | 0.975 | 0.980 | 0.877 | 0.785 | 0.683 | 0.646 | 0.684 | 0.549 | 0.508 | NA | NA | NA | 0.902 | 0.794 |
|  | Mis | NA | 0.130 | 0.130 | 0.116 | 0.084 | 0.071 | 0.028 | 0.030 | 0.050 | 0.076 | 0.092 | 0.072 | 0.121 | 0.149 | NA | NA | NA | 0.040 | 0.073 |
| Gini | Freq | 0 | 0 | 2 | 2 | 4 | 5 | 375 | 169 | 144 | 58 | 25 | 54 | 90 | 43 | 15 | 8 | 3 | 997 | 997 |
|  | Sen | NA | NA | 0.150 | 0.300 | 0.325 | 0.400 | 0.807 | 0.910 | 0.908 | 0.941 | 0.864 | 0.948 | 0.991 | 0.986 | 0.980 | 0.963 | 0.967 | 0.878 | 0.847 |
|  | PPV | NA | NA | 0.750 | 1.000 | 0.875 | 0.971 | 0.984 | 0.877 | 0.786 | 0.699 | 0.620 | 0.652 | 0.556 | 0.494 | 0.423 | 0.489 | 0.306 | 0.818 | 0.794 |
|  | Mis | NA | NA | 0.130 | 0.101 | 0.105 | 0.090 | 0.030 | 0.031 | 0.049 | 0.068 | 0.099 | 0.085 | 0.120 | 0.157 | 0.210 | 0.197 | 0.324 | 0.059 | 0.073 |
| Elbow | Freq | 0 | 1 | 5 | 9 | 32 | 32 | 623 | 191 | 73 | 25 | 5 | 1 | 0 | 0 | 0 | 0 | 0 | 997 | 997 |
|  | Sen | NA | 0.100 | 0.280 | 0.533 | 0.463 | 0.556 | 0.846 | 0.918 | 0.895 | 0.848 | 0.820 | 0.900 | NA | NA | NA | NA | NA | 0.835 | 0.847 |
|  | PPV | NA | 1.000 | 0.867 | 0.986 | 0.899 | 0.969 | 0.978 | 0.869 | 0.766 | 0.657 | 0.589 | 0.600 | NA | NA | NA | NA | NA | 0.928 | 0.794 |
|  | Mis | NA | 0.130 | 0.110 | 0.069 | 0.086 | 0.067 | 0.025 | 0.032 | 0.055 | 0.085 | 0.110 | 0.101 | NA | NA | NA | NA | NA | 0.035 | 0.073 |
| MCS  -P | Freq | 0 | 0 | 3 | 5 | 9 | 17 | 482 | 241 | 133 | 56 | 24 | 11 | 12 | 3 | 0 | 0 | 1 | 997 | 997 |
|  | Sen | NA | NA | 0.267 | 0.460 | 0.456 | 0.541 | 0.829 | 0.919 | 0.910 | 0.921 | 0.875 | 0.909 | 0.950 | 1.000 | NA | NA | 1.000 | 0.859 | 0.847 |
|  | PPV | NA | NA | 0.778 | 1.000 | 0.913 | 0.983 | 0.981 | 0.877 | 0.786 | 0.682 | 0.617 | 0.640 | 0.578 | 0.464 | NA | NA | 0.303 | 0.892 | 0.794 |
|  | Mis | NA | NA | 0.116 | 0.078 | 0.085 | 0.068 | 0.027 | 0.030 | 0.049 | 0.074 | 0.100 | 0.092 | 0.118 | 0.169 | NA | NA | 0.333 | 0.040 | 0.073 |
| MCHS  -P | Freq | 0 | 0 | 6 | 9 | 21 | 37 | 486 | 189 | 119 | 56 | 23 | 20 | 15 | 10 | 1 | 4 | 1 | 997 | 997 |
|  | Sen | NA | NA | 0.233 | 0.411 | 0.495 | 0.586 | 0.842 | 0.906 | 0.908 | 0.932 | 0.861 | 0.960 | 0.940 | 0.960 | 0.500 | 0.800 | 1.000 | 0.848 | 0.847 |
|  | PPV | NA | NA | 0.917 | 0.986 | 0.951 | 0.957 | 0.981 | 0.872 | 0.788 | 0.690 | 0.641 | 0.638 | 0.631 | 0.609 | 1.000 | 0.786 | 0.303 | 0.894 | 0.794 |
|  | Mis | NA | NA | 0.114 | 0.087 | 0.077 | 0.064 | 0.025 | 0.033 | 0.048 | 0.071 | 0.095 | 0.095 | 0.104 | 0.114 | 0.072 | 0.080 | 0.333 | 0.041 | 0.073 |

^a^Freq: frequency. ^b^Sen: sensitivity. ^c^PPV: positive predictive value. ^d^Mis: misclassification.

Table A40. Ordinal model: Simulation results for true cluster model (D) with an alternative hypothesis (6) using elliptical windows.

|  |  | Maximum reported cluster size (MRCS) | | | | | | | | | | | | | | | | | | Default  Setting |
| --- | --- | --- | --- | --- | --- | --- | --- | --- | --- | --- | --- | --- | --- | --- | --- | --- | --- | --- | --- | --- |
|  |  | 1% | 2% | 3% | 4% | 5% | 6% | 8% | 10% | 12% | 15% | 20% | 25% | 30% | 35% | 40% | 45% | 50% | Overall |  |
| SCIC_1_ | Freq^a^ | 0 | 3 | 4 | 12 | 32 | 35 | 419 | 128 | 85 | 24 | 49 | 110 | 65 | 25 | 3 | 2 | 3 | 999 | 999 |
|  | Sen^b^ | NA | 0.100 | 0.200 | 0.258 | 0.359 | 0.437 | 0.785 | 0.870 | 0.871 | 0.713 | 0.765 | 0.943 | 0.945 | 0.876 | 0.767 | 0.750 | 0.833 | 0.794 | 0.828 |
|  | PPV^c^ | NA | 1.000 | 1.000 | 0.979 | 0.983 | 0.978 | 0.981 | 0.879 | 0.774 | 0.701 | 0.672 | 0.663 | 0.578 | 0.588 | 0.587 | 0.672 | 0.542 | 0.854 | 0.778 |
|  | Mis^d^ | NA | 0.130 | 0.116 | 0.109 | 0.094 | 0.084 | 0.033 | 0.036 | 0.055 | 0.088 | 0.092 | 0.081 | 0.118 | 0.137 | 0.184 | 0.174 | 0.237 | 0.060 | 0.080 |
| SCIC_2_ | Freq | 0 | 1 | 1 | 11 | 23 | 32 | 513 | 199 | 112 | 36 | 23 | 27 | 13 | 6 | 1 | 0 | 1 | 999 | 999 |
|  | Sen | NA | 0.100 | 0.200 | 0.327 | 0.400 | 0.494 | 0.820 | 0.894 | 0.900 | 0.858 | 0.857 | 0.944 | 0.946 | 1.000 | 0.900 | NA | 1.000 | 0.826 | 0.828 |
|  | PPV | NA | 1.000 | 1.000 | 0.977 | 0.984 | 0.977 | 0.980 | 0.876 | 0.780 | 0.688 | 0.670 | 0.677 | 0.607 | 0.549 | 0.360 | NA | 0.313 | 0.902 | 0.778 |
|  | Mis | NA | 0.130 | 0.116 | 0.099 | 0.088 | 0.076 | 0.029 | 0.033 | 0.051 | 0.077 | 0.088 | 0.077 | 0.116 | 0.135 | 0.246 | NA | 0.319 | 0.043 | 0.080 |
| Gini | Freq | 0 | 1 | 0 | 0 | 10 | 9 | 365 | 174 | 134 | 57 | 38 | 50 | 88 | 48 | 17 | 2 | 6 | 999 | 999 |
|  | Sen | NA | 0.100 | NA | NA | 0.390 | 0.356 | 0.801 | 0.885 | 0.910 | 0.902 | 0.879 | 0.938 | 0.988 | 0.979 | 0.982 | 1.000 | 1.000 | 0.867 | 0.828 |
|  | PPV | NA | 1.000 | NA | NA | 0.967 | 1.000 | 0.990 | 0.874 | 0.787 | 0.687 | 0.648 | 0.634 | 0.564 | 0.488 | 0.412 | 0.352 | 0.310 | 0.817 | 0.778 |
|  | Mis | NA | 0.130 | NA | NA | 0.091 | 0.093 | 0.030 | 0.035 | 0.049 | 0.074 | 0.089 | 0.089 | 0.118 | 0.158 | 0.216 | 0.268 | 0.324 | 0.062 | 0.080 |
| Elbow | Freq | 0 | 1 | 1 | 8 | 32 | 38 | 624 | 182 | 84 | 22 | 6 | 1 | 0 | 0 | 0 | 0 | 0 | 999 | 999 |
|  | Sen | NA | 0.100 | 0.300 | 0.350 | 0.459 | 0.539 | 0.835 | 0.888 | 0.865 | 0.841 | 0.783 | 1.000 | NA | NA | NA | NA | NA | 0.819 | 0.828 |
|  | PPV | NA | 1.000 | 1.000 | 0.969 | 0.957 | 0.965 | 0.979 | 0.862 | 0.766 | 0.672 | 0.579 | 0.714 | NA | NA | NA | NA | NA | 0.929 | 0.778 |
|  | Mis | NA | 0.130 | 0.101 | 0.096 | 0.083 | 0.070 | 0.027 | 0.037 | 0.057 | 0.082 | 0.114 | 0.058 | NA | NA | NA | NA | NA | 0.037 | 0.080 |
| MCS  -P | Freq | 0 | 1 | 0 | 4 | 17 | 17 | 480 | 227 | 140 | 52 | 25 | 11 | 12 | 10 | 1 | 0 | 2 | 999 | 999 |
|  | Sen | NA | 0.100 | NA | 0.400 | 0.453 | 0.429 | 0.825 | 0.904 | 0.904 | 0.894 | 0.844 | 0.900 | 0.942 | 0.980 | 1.000 | NA | 1.000 | 0.847 | 0.828 |
|  | PPV | NA | 1.000 | NA | 0.938 | 0.948 | 0.990 | 0.986 | 0.874 | 0.782 | 0.685 | 0.622 | 0.657 | 0.661 | 0.466 | 0.400 | NA | 0.313 | 0.892 | 0.778 |
|  | Mis | NA | 0.130 | NA | 0.091 | 0.085 | 0.084 | 0.027 | 0.033 | 0.050 | 0.075 | 0.099 | 0.087 | 0.098 | 0.167 | 0.217 | NA | 0.319 | 0.042 | 0.080 |
| MCHS  -P | Freq | 0 | 2 | 2 | 9 | 33 | 47 | 446 | 198 | 117 | 53 | 35 | 19 | 22 | 13 | 1 | 0 | 2 | 999 | 999 |
|  | Sen | NA | 0.150 | 0.350 | 0.400 | 0.500 | 0.547 | 0.828 | 0.893 | 0.897 | 0.883 | 0.866 | 0.889 | 0.968 | 0.969 | 1.000 | NA | 1.000 | 0.830 | 0.828 |
|  | PPV | NA | 1.000 | 1.000 | 1.000 | 0.980 | 0.979 | 0.986 | 0.867 | 0.784 | 0.690 | 0.688 | 0.628 | 0.623 | 0.517 | 0.400 | NA | 0.313 | 0.889 | 0.778 |
|  | Mis | NA | 0.123 | 0.094 | 0.087 | 0.074 | 0.068 | 0.027 | 0.035 | 0.050 | 0.075 | 0.082 | 0.097 | 0.104 | 0.147 | 0.217 | NA | 0.319 | 0.045 | 0.080 |

^a^Freq: frequency. ^b^Sen: sensitivity. ^c^PPV: positive predictive value. ^d^Mis: misclassification.

Table A41. Ordinal model: Simulation results for true cluster model (D) with an alternative hypothesis (7) using elliptical windows.

|  |  | Maximum reported cluster size (MRCS) | | | | | | | | | | | | | | | | | | Default  Setting |
| --- | --- | --- | --- | --- | --- | --- | --- | --- | --- | --- | --- | --- | --- | --- | --- | --- | --- | --- | --- | --- |
|  |  | 1% | 2% | 3% | 4% | 5% | 6% | 8% | 10% | 12% | 15% | 20% | 25% | 30% | 35% | 40% | 45% | 50% | Overall |  |
| SCIC_1_ | Freq^a^ | 0 | 3 | 5 | 7 | 28 | 47 | 459 | 150 | 90 | 38 | 36 | 68 | 45 | 9 | 8 | 3 | 1 | 997 | 997 |
|  | Sen^b^ | NA | 0.100 | 0.200 | 0.243 | 0.364 | 0.500 | 0.808 | 0.883 | 0.877 | 0.839 | 0.817 | 0.915 | 0.920 | 0.944 | 0.625 | 0.833 | 1.000 | 0.804 | 0.836 |
|  | PPV^c^ | NA | 1.000 | 1.000 | 0.917 | 0.988 | 0.970 | 0.983 | 0.876 | 0.775 | 0.693 | 0.667 | 0.675 | 0.586 | 0.538 | 0.814 | 0.567 | 0.303 | 0.878 | 0.810 |
|  | Mis^d^ | NA | 0.130 | 0.116 | 0.114 | 0.093 | 0.075 | 0.030 | 0.035 | 0.054 | 0.079 | 0.089 | 0.082 | 0.120 | 0.148 | 0.112 | 0.203 | 0.333 | 0.052 | 0.068 |
| SCIC_2_ | Freq | 0 | 2 | 3 | 8 | 19 | 47 | 534 | 177 | 108 | 51 | 17 | 16 | 11 | 2 | 0 | 1 | 1 | 997 | 997 |
|  | Sen | NA | 0.100 | 0.200 | 0.288 | 0.495 | 0.543 | 0.830 | 0.894 | 0.902 | 0.894 | 0.906 | 0.944 | 0.918 | 1.000 | NA | 1.000 | 1.000 | 0.829 | 0.836 |
|  | PPV | NA | 1.000 | 1.000 | 0.969 | 0.992 | 0.970 | 0.981 | 0.877 | 0.778 | 0.686 | 0.685 | 0.713 | 0.625 | 0.488 | NA | 0.345 | 0.303 | 0.909 | 0.810 |
|  | Mis | NA | 0.130 | 0.116 | 0.105 | 0.074 | 0.069 | 0.027 | 0.033 | 0.052 | 0.076 | 0.078 | 0.071 | 0.104 | 0.152 | NA | 0.275 | 0.333 | 0.041 | 0.068 |
| Gini | Freq | 0 | 1 | 1 | 1 | 7 | 6 | 403 | 173 | 132 | 69 | 32 | 49 | 70 | 39 | 12 | 2 | 0 | 997 | 997 |
|  | Sen | NA | 0.200 | 0.200 | 0.200 | 0.429 | 0.433 | 0.816 | 0.894 | 0.889 | 0.926 | 0.938 | 0.941 | 0.969 | 0.997 | 0.975 | 1.000 | NA | 0.870 | 0.836 |
|  | PPV | NA | 1.000 | 1.000 | 1.000 | 0.913 | 1.000 | 0.987 | 0.874 | 0.781 | 0.682 | 0.625 | 0.659 | 0.558 | 0.486 | 0.450 | 0.358 | NA | 0.834 | 0.810 |
|  | Mis | NA | 0.116 | 0.116 | 0.116 | 0.089 | 0.082 | 0.028 | 0.034 | 0.052 | 0.074 | 0.095 | 0.085 | 0.121 | 0.159 | 0.197 | 0.261 | NA | 0.056 | 0.068 |
| Elbow | Freq | 0 | 1 | 5 | 11 | 28 | 44 | 659 | 162 | 66 | 18 | 1 | 0 | 2 | 0 | 0 | 0 | 0 | 997 | 997 |
|  | Sen | NA | 0.200 | 0.300 | 0.509 | 0.507 | 0.555 | 0.845 | 0.892 | 0.891 | 0.861 | 0.800 | NA | 0.750 | NA | NA | NA | NA | 0.826 | 0.836 |
|  | PPV | NA | 1.000 | 0.967 | 0.989 | 0.937 | 0.951 | 0.979 | 0.864 | 0.754 | 0.663 | 0.667 | NA | 0.417 | NA | NA | NA | NA | 0.936 | 0.810 |
|  | Mis | NA | 0.116 | 0.104 | 0.072 | 0.077 | 0.069 | 0.025 | 0.036 | 0.059 | 0.085 | 0.087 | NA | 0.188 | NA | NA | NA | NA | 0.035 | 0.068 |
| MCS  -P | Freq | 0 | 1 | 2 | 8 | 20 | 29 | 503 | 185 | 127 | 61 | 25 | 14 | 15 | 5 | 0 | 1 | 1 | 997 | 997 |
|  | Sen | NA | 0.200 | 0.350 | 0.463 | 0.505 | 0.559 | 0.832 | 0.882 | 0.887 | 0.907 | 0.900 | 0.943 | 0.900 | 1.000 | NA | 1.000 | 1.000 | 0.839 | 0.836 |
|  | PPV | NA | 1.000 | 0.917 | 0.984 | 0.961 | 0.976 | 0.981 | 0.872 | 0.776 | 0.687 | 0.654 | 0.701 | 0.571 | 0.512 | NA | 0.345 | 0.303 | 0.894 | 0.810 |
|  | Mis | NA | 0.116 | 0.101 | 0.080 | 0.075 | 0.066 | 0.027 | 0.036 | 0.053 | 0.074 | 0.092 | 0.077 | 0.129 | 0.142 | NA | 0.275 | 0.333 | 0.043 | 0.068 |
| MCHS  -P | Freq | 0 | 1 | 6 | 7 | 20 | 16 | 489 | 188 | 139 | 63 | 29 | 16 | 14 | 6 | 1 | 1 | 1 | 997 | 997 |
|  | Sen | NA | 0.200 | 0.283 | 0.443 | 0.490 | 0.513 | 0.845 | 0.884 | 0.894 | 0.913 | 0.955 | 0.944 | 0.879 | 0.950 | 1.000 | 1.000 | 1.000 | 0.850 | 0.836 |
|  | PPV | NA | 1.000 | 1.000 | 0.982 | 0.940 | 0.982 | 0.987 | 0.869 | 0.778 | 0.675 | 0.646 | 0.700 | 0.616 | 0.627 | 0.556 | 0.345 | 0.303 | 0.891 | 0.810 |
|  | Mis | NA | 0.116 | 0.104 | 0.083 | 0.078 | 0.072 | 0.024 | 0.036 | 0.052 | 0.077 | 0.087 | 0.080 | 0.118 | 0.106 | 0.116 | 0.275 | 0.333 | 0.042 | 0.068 |

^a^Freq: frequency. ^b^Sen: sensitivity. ^c^PPV: positive predictive value. ^d^Mis: misclassification.

Table A42. Ordinal model: Simulation results for true cluster model (E) with an alternative hypothesis (1) using elliptical windows.

|  |  | Maximum reported cluster size (MRCS) | | | | | | | | | | | | | | | | | | Default  Setting |
| --- | --- | --- | --- | --- | --- | --- | --- | --- | --- | --- | --- | --- | --- | --- | --- | --- | --- | --- | --- | --- |
|  |  | 1% | 2% | 3% | 4% | 5% | 6% | 8% | 10% | 12% | 15% | 20% | 25% | 30% | 35% | 40% | 45% | 50% | Overall |  |
| SCIC_1_ | Freq^a^ | 0 | 1 | 2 | 4 | 17 | 29 | 584 | 237 | 70 | 20 | 9 | 3 | 3 | 2 | 3 | 6 | 5 | 995 | 995 |
|  | Sen^b^ | NA | 0.100 | 0.200 | 0.200 | 0.394 | 0.431 | 0.824 | 0.897 | 0.896 | 0.915 | 0.867 | 0.733 | 0.767 | 0.700 | 0.900 | 0.617 | 0.880 | 0.824 | 0.850 |
|  | PPV^c^ | NA | 1.000 | 1.000 | 0.917 | 0.980 | 0.967 | 0.979 | 0.866 | 0.767 | 0.690 | 0.574 | 0.586 | 0.601 | 0.612 | 0.531 | 0.772 | 0.400 | 0.919 | 0.844 |
|  | Mis^d^ | NA | 0.130 | 0.116 | 0.120 | 0.089 | 0.085 | 0.028 | 0.035 | 0.054 | 0.072 | 0.111 | 0.135 | 0.150 | 0.152 | 0.184 | 0.116 | 0.296 | 0.040 | 0.060 |
| SCIC_2_ | Freq | 0 | 0 | 2 | 3 | 19 | 28 | 585 | 239 | 73 | 29 | 9 | 2 | 1 | 0 | 2 | 2 | 1 | 995 | 995 |
|  | Sen | NA | NA | 0.200 | 0.433 | 0.447 | 0.511 | 0.841 | 0.900 | 0.908 | 0.914 | 0.933 | 0.800 | 0.800 | NA | 0.900 | 0.700 | 0.700 | 0.843 | 0.850 |
|  | PPV | NA | NA | 1.000 | 1.000 | 0.957 | 0.949 | 0.980 | 0.867 | 0.768 | 0.668 | 0.601 | 0.692 | 0.381 | NA | 0.617 | 0.739 | 0.875 | 0.921 | 0.844 |
|  | Mis | NA | NA | 0.116 | 0.082 | 0.084 | 0.077 | 0.026 | 0.034 | 0.053 | 0.079 | 0.101 | 0.101 | 0.217 | NA | 0.152 | 0.080 | 0.058 | 0.036 | 0.060 |
| Gini | Freq | 0 | 0 | 0 | 3 | 12 | 13 | 398 | 284 | 127 | 81 | 24 | 8 | 5 | 6 | 8 | 12 | 14 | 995 | 995 |
|  | Sen | NA | NA | NA | 0.500 | 0.533 | 0.454 | 0.821 | 0.909 | 0.912 | 0.912 | 0.921 | 0.988 | 0.880 | 0.900 | 0.925 | 0.917 | 0.964 | 0.864 | 0.850 |
|  | PPV | NA | NA | NA | 1.000 | 0.925 | 0.958 | 0.981 | 0.872 | 0.774 | 0.679 | 0.579 | 0.511 | 0.676 | 0.409 | 0.345 | 0.339 | 0.296 | 0.857 | 0.844 |
|  | Mis | NA | NA | NA | 0.072 | 0.075 | 0.084 | 0.028 | 0.032 | 0.051 | 0.076 | 0.110 | 0.141 | 0.099 | 0.210 | 0.266 | 0.284 | 0.339 | 0.051 | 0.060 |
| Elbow | Freq | 0 | 0 | 4 | 22 | 45 | 40 | 647 | 179 | 45 | 11 | 2 | 0 | 0 | 0 | 0 | 0 | 0 | 995 | 995 |
|  | Sen | NA | NA | 0.375 | 0.595 | 0.627 | 0.565 | 0.854 | 0.892 | 0.873 | 0.882 | 0.850 | NA | NA | NA | NA | NA | NA | 0.832 | 0.850 |
|  | PPV | NA | NA | 1.000 | 0.979 | 0.931 | 0.920 | 0.978 | 0.861 | 0.723 | 0.647 | 0.572 | NA | NA | NA | NA | NA | NA | 0.937 | 0.844 |
|  | Mis | NA | NA | 0.091 | 0.061 | 0.062 | 0.073 | 0.024 | 0.036 | 0.068 | 0.088 | 0.116 | NA | NA | NA | NA | NA | NA | 0.034 | 0.060 |
| MCS  -P | Freq | 0 | 0 | 0 | 8 | 19 | 23 | 499 | 272 | 101 | 48 | 14 | 4 | 1 | 0 | 3 | 2 | 1 | 995 | 995 |
|  | Sen | NA | NA | NA | 0.525 | 0.558 | 0.522 | 0.834 | 0.910 | 0.913 | 0.925 | 0.957 | 1.000 | 1.000 | NA | 0.933 | 0.750 | 1.000 | 0.855 | 0.850 |
|  | PPV | NA | NA | NA | 0.979 | 0.933 | 0.932 | 0.979 | 0.869 | 0.772 | 0.678 | 0.593 | 0.521 | 0.667 | NA | 0.335 | 0.532 | 0.323 | 0.901 | 0.844 |
|  | Mis | NA | NA | NA | 0.071 | 0.071 | 0.078 | 0.027 | 0.033 | 0.051 | 0.076 | 0.105 | 0.138 | 0.072 | NA | 0.280 | 0.196 | 0.304 | 0.039 | 0.060 |
| MCHS  -P | Freq | 0 | 0 | 2 | 14 | 38 | 39 | 453 | 236 | 108 | 66 | 21 | 7 | 4 | 1 | 4 | 1 | 1 | 995 | 995 |
|  | Sen | NA | NA | 0.250 | 0.507 | 0.545 | 0.567 | 0.833 | 0.909 | 0.908 | 0.918 | 0.938 | 0.986 | 0.875 | 1.000 | 0.950 | 0.800 | 1.000 | 0.842 | 0.850 |
|  | PPV | NA | NA | 1.000 | 0.986 | 0.961 | 0.959 | 0.977 | 0.871 | 0.772 | 0.681 | 0.589 | 0.509 | 0.841 | 0.400 | 0.430 | 0.286 | 0.323 | 0.893 | 0.844 |
|  | Mis | NA | NA | 0.109 | 0.072 | 0.070 | 0.068 | 0.027 | 0.033 | 0.052 | 0.075 | 0.108 | 0.143 | 0.043 | 0.217 | 0.225 | 0.319 | 0.304 | 0.043 | 0.060 |

^a^Freq: frequency. ^b^Sen: sensitivity. ^c^PPV: positive predictive value. ^d^Mis: misclassification.

Table A43. Ordinal model: Simulation results for true cluster model (E) with an alternative hypothesis (2) using elliptical windows.

|  |  | Maximum reported cluster size (MRCS) | | | | | | | | | | | | | | | | | | Default  Setting |
| --- | --- | --- | --- | --- | --- | --- | --- | --- | --- | --- | --- | --- | --- | --- | --- | --- | --- | --- | --- | --- |
|  |  | 1% | 2% | 3% | 4% | 5% | 6% | 8% | 10% | 12% | 15% | 20% | 25% | 30% | 35% | 40% | 45% | 50% | Overall |  |
| SCIC_1_ | Freq^a^ | 0 | 2 | 4 | 16 | 40 | 53 | 509 | 191 | 80 | 38 | 12 | 4 | 3 | 4 | 7 | 3 | 8 | 974 | 974 |
|  | Sen^b^ | NA | 0.100 | 0.200 | 0.244 | 0.328 | 0.396 | 0.737 | 0.805 | 0.818 | 0.816 | 0.675 | 0.650 | 0.567 | 0.725 | 0.757 | 0.933 | 0.950 | 0.714 | 0.770 |
|  | PPV^c^ | NA | 1.000 | 1.000 | 0.906 | 0.973 | 0.936 | 0.965 | 0.837 | 0.755 | 0.633 | 0.496 | 0.473 | 0.508 | 0.405 | 0.509 | 0.315 | 0.348 | 0.886 | 0.805 |
|  | Mis^d^ | NA | 0.130 | 0.116 | 0.113 | 0.099 | 0.092 | 0.042 | 0.050 | 0.064 | 0.094 | 0.144 | 0.156 | 0.179 | 0.207 | 0.209 | 0.304 | 0.301 | 0.061 | 0.080 |
| SCIC_2_ | Freq | 0 | 2 | 2 | 11 | 24 | 44 | 508 | 213 | 93 | 44 | 19 | 4 | 1 | 3 | 1 | 0 | 5 | 974 | 974 |
|  | Sen | NA | 0.100 | 0.200 | 0.255 | 0.375 | 0.423 | 0.759 | 0.831 | 0.835 | 0.832 | 0.789 | 0.775 | 0.500 | 0.833 | 0.800 | NA | 0.940 | 0.754 | 0.770 |
|  | PPV | NA | 1.000 | 1.000 | 0.939 | 0.950 | 0.931 | 0.965 | 0.839 | 0.754 | 0.631 | 0.532 | 0.465 | 0.278 | 0.373 | 0.320 | NA | 0.381 | 0.884 | 0.805 |
|  | Mis | NA | 0.130 | 0.116 | 0.111 | 0.094 | 0.089 | 0.039 | 0.047 | 0.063 | 0.094 | 0.128 | 0.163 | 0.261 | 0.227 | 0.275 | NA | 0.275 | 0.055 | 0.080 |
| Gini | Freq | 0 | 0 | 1 | 3 | 17 | 26 | 367 | 239 | 122 | 80 | 38 | 14 | 10 | 5 | 10 | 10 | 32 | 974 | 974 |
|  | Sen | NA | NA | 0.200 | 0.267 | 0.365 | 0.404 | 0.731 | 0.838 | 0.840 | 0.853 | 0.818 | 0.836 | 0.790 | 0.840 | 0.900 | 0.920 | 0.922 | 0.780 | 0.770 |
|  | PPV | NA | NA | 1.000 | 0.889 | 0.941 | 0.936 | 0.965 | 0.851 | 0.757 | 0.650 | 0.541 | 0.457 | 0.388 | 0.353 | 0.347 | 0.437 | 0.303 | 0.817 | 0.805 |
|  | Mis | NA | NA | 0.116 | 0.111 | 0.095 | 0.091 | 0.043 | 0.044 | 0.062 | 0.087 | 0.125 | 0.166 | 0.216 | 0.246 | 0.261 | 0.220 | 0.331 | 0.073 | 0.080 |
| Elbow | Freq | 0 | 1 | 4 | 9 | 34 | 48 | 566 | 206 | 65 | 27 | 8 | 2 | 0 | 2 | 1 | 0 | 0 | 973 | 974 |
|  | Sen | NA | 0.100 | 0.350 | 0.378 | 0.447 | 0.442 | 0.767 | 0.829 | 0.808 | 0.733 | 0.788 | 0.800 | NA | 0.850 | 0.800 | NA | NA | 0.749 | 0.770 |
|  | PPV | NA | 1.000 | 1.000 | 0.963 | 0.930 | 0.919 | 0.961 | 0.831 | 0.731 | 0.588 | 0.509 | 0.475 | NA | 0.369 | 0.320 | NA | NA | 0.898 | 0.805 |
|  | Mis | NA | 0.130 | 0.094 | 0.092 | 0.086 | 0.088 | 0.038 | 0.049 | 0.070 | 0.111 | 0.138 | 0.159 | NA | 0.232 | 0.275 | NA | NA | 0.051 | 0.080 |
| MCS  -P | Freq | 0 | 0 | 2 | 5 | 19 | 32 | 444 | 231 | 117 | 68 | 29 | 10 | 2 | 2 | 3 | 1 | 9 | 974 | 974 |
|  | Sen | NA | NA | 0.350 | 0.240 | 0.363 | 0.416 | 0.749 | 0.842 | 0.843 | 0.838 | 0.828 | 0.780 | 0.600 | 0.800 | 0.867 | 0.800 | 0.933 | 0.771 | 0.770 |
|  | PPV | NA | NA | 1.000 | 0.867 | 0.943 | 0.931 | 0.965 | 0.847 | 0.755 | 0.637 | 0.542 | 0.447 | 0.314 | 0.372 | 0.347 | 0.667 | 0.335 | 0.858 | 0.805 |
|  | Mis | NA | NA | 0.094 | 0.116 | 0.095 | 0.090 | 0.040 | 0.044 | 0.062 | 0.091 | 0.124 | 0.171 | 0.246 | 0.225 | 0.256 | 0.087 | 0.312 | 0.058 | 0.080 |
| MCHS  -P | Freq | 0 | 0 | 2 | 12 | 29 | 38 | 432 | 220 | 107 | 68 | 26 | 16 | 7 | 3 | 3 | 2 | 9 | 974 | 974 |
|  | Sen | NA | NA | 0.350 | 0.375 | 0.445 | 0.447 | 0.750 | 0.837 | 0.837 | 0.849 | 0.796 | 0.856 | 0.829 | 0.833 | 0.867 | 0.850 | 0.933 | 0.766 | 0.770 |
|  | PPV | NA | NA | 1.000 | 0.911 | 0.942 | 0.944 | 0.963 | 0.849 | 0.752 | 0.648 | 0.535 | 0.522 | 0.390 | 0.373 | 0.347 | 0.742 | 0.335 | 0.858 | 0.805 |
|  | Mis | NA | NA | 0.094 | 0.095 | 0.084 | 0.084 | 0.040 | 0.045 | 0.063 | 0.087 | 0.129 | 0.146 | 0.219 | 0.227 | 0.256 | 0.065 | 0.312 | 0.060 | 0.080 |

^a^Freq: frequency. ^b^Sen: sensitivity. ^c^PPV: positive predictive value. ^d^Mis: misclassification.

Table A44. Ordinal model: Simulation results for true cluster model (E) with an alternative hypothesis (3) using elliptical windows.

|  |  | Maximum reported cluster size (MRCS) | | | | | | | | | | | | | | | | | | Default  Setting |
| --- | --- | --- | --- | --- | --- | --- | --- | --- | --- | --- | --- | --- | --- | --- | --- | --- | --- | --- | --- | --- |
|  |  | 1% | 2% | 3% | 4% | 5% | 6% | 8% | 10% | 12% | 15% | 20% | 25% | 30% | 35% | 40% | 45% | 50% | Overall |  |
| SCIC_1_ | Freq^a^ | 0 | 5 | 7 | 13 | 42 | 50 | 506 | 204 | 69 | 30 | 13 | 5 | 6 | 3 | 7 | 7 | 8 | 975 | 975 |
|  | Sen^b^ | NA | 0.100 | 0.171 | 0.215 | 0.307 | 0.402 | 0.725 | 0.795 | 0.784 | 0.780 | 0.631 | 0.680 | 0.617 | 0.467 | 0.843 | 0.829 | 0.788 | 0.696 | 0.747 |
|  | PPV^c^ | NA | 1.000 | 0.929 | 0.929 | 0.944 | 0.902 | 0.973 | 0.842 | 0.726 | 0.640 | 0.490 | 0.580 | 0.658 | 0.944 | 0.390 | 0.507 | 0.476 | 0.890 | 0.812 |
|  | Mis^d^ | NA | 0.130 | 0.122 | 0.117 | 0.103 | 0.093 | 0.043 | 0.050 | 0.072 | 0.093 | 0.148 | 0.133 | 0.140 | 0.082 | 0.236 | 0.205 | 0.254 | 0.062 | 0.080 |
| SCIC_2_ | Freq | 0 | 4 | 2 | 13 | 28 | 45 | 516 | 221 | 82 | 33 | 18 | 3 | 2 | 0 | 2 | 4 | 2 | 975 | 975 |
|  | Sen | NA | 0.100 | 0.200 | 0.238 | 0.350 | 0.456 | 0.745 | 0.806 | 0.829 | 0.785 | 0.806 | 0.867 | 0.850 | NA | 0.900 | 0.875 | 1.000 | 0.735 | 0.747 |
|  | PPV | NA | 1.000 | 1.000 | 0.955 | 0.944 | 0.896 | 0.970 | 0.843 | 0.734 | 0.638 | 0.522 | 0.583 | 0.609 | NA | 0.360 | 0.554 | 0.294 | 0.892 | 0.812 |
|  | Mis | NA | 0.130 | 0.116 | 0.113 | 0.098 | 0.087 | 0.040 | 0.049 | 0.066 | 0.093 | 0.133 | 0.116 | 0.123 | NA | 0.246 | 0.181 | 0.348 | 0.055 | 0.080 |
| Gini | Freq | 0 | 0 | 1 | 3 | 21 | 26 | 361 | 247 | 132 | 73 | 41 | 11 | 12 | 5 | 13 | 14 | 15 | 975 | 975 |
|  | Sen | NA | NA | 0.200 | 0.200 | 0.371 | 0.435 | 0.712 | 0.807 | 0.830 | 0.807 | 0.837 | 0.882 | 0.867 | 0.900 | 0.915 | 0.929 | 0.933 | 0.762 | 0.747 |
|  | PPV | NA | NA | 1.000 | 1.000 | 0.953 | 0.896 | 0.974 | 0.850 | 0.743 | 0.648 | 0.537 | 0.476 | 0.505 | 0.434 | 0.352 | 0.374 | 0.284 | 0.824 | 0.812 |
|  | Mis | NA | NA | 0.116 | 0.116 | 0.095 | 0.090 | 0.044 | 0.047 | 0.065 | 0.089 | 0.127 | 0.162 | 0.159 | 0.191 | 0.258 | 0.264 | 0.352 | 0.072 | 0.080 |
| Elbow | Freq | 0 | 1 | 4 | 15 | 40 | 50 | 580 | 196 | 61 | 17 | 9 | 1 | 0 | 0 | 1 | 0 | 0 | 975 | 975 |
|  | Sen | NA | 0.100 | 0.275 | 0.313 | 0.413 | 0.476 | 0.762 | 0.793 | 0.808 | 0.612 | 0.856 | 0.700 | NA | NA | 0.900 | NA | NA | 0.731 | 0.747 |
|  | PPV | NA | 1.000 | 1.000 | 0.961 | 0.929 | 0.891 | 0.971 | 0.832 | 0.717 | 0.568 | 0.517 | 0.500 | NA | NA | 0.360 | NA | NA | 0.909 | 0.812 |
|  | Mis | NA | 0.130 | 0.105 | 0.101 | 0.091 | 0.084 | 0.038 | 0.052 | 0.072 | 0.122 | 0.135 | 0.145 | NA | NA | 0.246 | NA | NA | 0.051 | 0.080 |
| MCS  -P | Freq | 0 | 1 | 2 | 6 | 29 | 32 | 448 | 231 | 103 | 62 | 36 | 5 | 3 | 3 | 7 | 3 | 4 | 975 | 975 |
|  | Sen | NA | 0.100 | 0.300 | 0.300 | 0.379 | 0.444 | 0.738 | 0.796 | 0.856 | 0.803 | 0.872 | 0.960 | 0.867 | 0.800 | 0.900 | 0.900 | 0.925 | 0.753 | 0.747 |
|  | PPV | NA | 1.000 | 1.000 | 1.000 | 0.938 | 0.893 | 0.975 | 0.844 | 0.744 | 0.642 | 0.559 | 0.538 | 0.542 | 0.391 | 0.360 | 0.472 | 0.270 | 0.865 | 0.812 |
|  | Mis | NA | 0.130 | 0.101 | 0.101 | 0.095 | 0.089 | 0.041 | 0.050 | 0.063 | 0.091 | 0.119 | 0.136 | 0.150 | 0.213 | 0.248 | 0.222 | 0.373 | 0.060 | 0.080 |
| MCHS  -P | Freq | 0 | 1 | 6 | 14 | 48 | 63 | 394 | 214 | 109 | 55 | 32 | 9 | 7 | 6 | 8 | 4 | 5 | 975 | 975 |
|  | Sen | NA | 0.100 | 0.250 | 0.350 | 0.408 | 0.484 | 0.722 | 0.796 | 0.835 | 0.800 | 0.838 | 0.978 | 0.786 | 0.733 | 0.888 | 0.775 | 0.900 | 0.725 | 0.747 |
|  | PPV | NA | 1.000 | 1.000 | 0.982 | 0.960 | 0.918 | 0.974 | 0.843 | 0.739 | 0.644 | 0.546 | 0.521 | 0.562 | 0.568 | 0.353 | 0.429 | 0.264 | 0.862 | 0.812 |
|  | Mis | NA | 0.130 | 0.109 | 0.095 | 0.088 | 0.081 | 0.043 | 0.050 | 0.065 | 0.090 | 0.125 | 0.140 | 0.143 | 0.169 | 0.254 | 0.261 | 0.377 | 0.065 | 0.080 |

^a^Freq: frequency. ^b^Sen: sensitivity. ^c^PPV: positive predictive value. ^d^Mis: misclassification.

Table A45. Ordinal model: Simulation results for true cluster model (E) with an alternative hypothesis (4) using elliptical windows.

|  |  | Maximum reported cluster size (MRCS) | | | | | | | | | | | | | | | | | | Default  Setting |
| --- | --- | --- | --- | --- | --- | --- | --- | --- | --- | --- | --- | --- | --- | --- | --- | --- | --- | --- | --- | --- |
|  |  | 1% | 2% | 3% | 4% | 5% | 6% | 8% | 10% | 12% | 15% | 20% | 25% | 30% | 35% | 40% | 45% | 50% | Overall |  |
| SCIC_1_ | Freq^a^ | 0 | 6 | 10 | 29 | 49 | 58 | 523 | 155 | 59 | 17 | 16 | 12 | 12 | 5 | 8 | 5 | 7 | 971 | 971 |
|  | Sen^b^ | NA | 0.117 | 0.180 | 0.234 | 0.343 | 0.412 | 0.700 | 0.763 | 0.753 | 0.735 | 0.625 | 0.592 | 0.650 | 0.680 | 0.900 | 0.760 | 0.829 | 0.655 | 0.717 |
|  | PPV^c^ | NA | 1.000 | 0.950 | 0.933 | 0.939 | 0.968 | 0.968 | 0.837 | 0.724 | 0.595 | 0.515 | 0.660 | 0.558 | 0.699 | 0.347 | 0.448 | 0.493 | 0.894 | 0.803 |
|  | Mis^d^ | NA | 0.128 | 0.120 | 0.114 | 0.098 | 0.087 | 0.047 | 0.055 | 0.076 | 0.107 | 0.141 | 0.132 | 0.159 | 0.128 | 0.261 | 0.217 | 0.269 | 0.068 | 0.090 |
| SCIC_2_ | Freq | 0 | 3 | 7 | 23 | 38 | 56 | 533 | 176 | 66 | 34 | 13 | 6 | 5 | 1 | 4 | 2 | 4 | 971 | 971 |
|  | Sen | NA | 0.133 | 0.200 | 0.257 | 0.342 | 0.455 | 0.720 | 0.791 | 0.773 | 0.838 | 0.623 | 0.683 | 0.680 | 0.900 | 0.850 | 1.000 | 0.975 | 0.695 | 0.717 |
|  | PPV | NA | 1.000 | 0.929 | 0.941 | 0.928 | 0.963 | 0.969 | 0.838 | 0.726 | 0.650 | 0.494 | 0.505 | 0.415 | 0.900 | 0.472 | 0.345 | 0.296 | 0.896 | 0.803 |
|  | Mis | NA | 0.126 | 0.118 | 0.111 | 0.100 | 0.082 | 0.044 | 0.052 | 0.074 | 0.087 | 0.152 | 0.169 | 0.203 | 0.029 | 0.217 | 0.275 | 0.341 | 0.061 | 0.090 |
| Gini | Freq | 0 | 1 | 2 | 7 | 22 | 27 | 399 | 203 | 119 | 62 | 38 | 10 | 13 | 8 | 13 | 22 | 25 | 971 | 971 |
|  | Sen | NA | 0.100 | 0.200 | 0.257 | 0.336 | 0.441 | 0.695 | 0.779 | 0.809 | 0.835 | 0.761 | 0.810 | 0.746 | 0.763 | 0.877 | 0.941 | 0.940 | 0.735 | 0.717 |
|  | PPV | NA | 1.000 | 1.000 | 1.000 | 0.909 | 0.969 | 0.970 | 0.840 | 0.747 | 0.651 | 0.520 | 0.517 | 0.411 | 0.349 | 0.384 | 0.350 | 0.316 | 0.821 | 0.803 |
|  | Mis | NA | 0.130 | 0.116 | 0.108 | 0.102 | 0.083 | 0.047 | 0.053 | 0.066 | 0.087 | 0.135 | 0.146 | 0.198 | 0.243 | 0.249 | 0.275 | 0.326 | 0.079 | 0.090 |
| Elbow | Freq | 0 | 3 | 5 | 28 | 47 | 61 | 588 | 157 | 50 | 17 | 10 | 2 | 1 | 0 | 1 | 0 | 0 | 970 | 971 |
|  | Sen | NA | 0.167 | 0.220 | 0.325 | 0.394 | 0.495 | 0.733 | 0.778 | 0.702 | 0.782 | 0.620 | 0.750 | 0.800 | NA | 0.800 | NA | NA | 0.691 | 0.717 |
|  | PPV | NA | 1.000 | 1.000 | 0.935 | 0.935 | 0.948 | 0.967 | 0.824 | 0.690 | 0.623 | 0.412 | 0.417 | 0.421 | NA | 0.308 | NA | NA | 0.912 | 0.803 |
|  | Mis | NA | 0.121 | 0.113 | 0.101 | 0.092 | 0.078 | 0.042 | 0.055 | 0.088 | 0.096 | 0.183 | 0.181 | 0.188 | NA | 0.290 | NA | NA | 0.057 | 0.090 |
| MCS  -P | Freq | 0 | 2 | 3 | 14 | 29 | 40 | 467 | 184 | 105 | 56 | 28 | 8 | 6 | 4 | 10 | 7 | 8 | 971 | 971 |
|  | Sen | NA | 0.200 | 0.233 | 0.257 | 0.338 | 0.458 | 0.719 | 0.799 | 0.810 | 0.834 | 0.739 | 0.638 | 0.767 | 0.675 | 0.880 | 1.000 | 0.925 | 0.725 | 0.717 |
|  | PPV | NA | 1.000 | 1.000 | 0.940 | 0.931 | 0.957 | 0.973 | 0.841 | 0.745 | 0.657 | 0.514 | 0.524 | 0.444 | 0.494 | 0.397 | 0.339 | 0.285 | 0.864 | 0.803 |
|  | Mis | NA | 0.116 | 0.111 | 0.111 | 0.100 | 0.082 | 0.044 | 0.050 | 0.067 | 0.085 | 0.140 | 0.161 | 0.186 | 0.181 | 0.243 | 0.284 | 0.348 | 0.066 | 0.090 |
| MCHS  -P | Freq | 0 | 3 | 5 | 21 | 26 | 35 | 426 | 190 | 118 | 63 | 36 | 8 | 7 | 5 | 10 | 8 | 10 | 971 | 971 |
|  | Sen | NA | 0.200 | 0.220 | 0.290 | 0.315 | 0.446 | 0.706 | 0.806 | 0.825 | 0.848 | 0.761 | 0.713 | 0.771 | 0.740 | 0.880 | 1.000 | 0.930 | 0.726 | 0.717 |
|  | PPV | NA | 1.000 | 1.000 | 0.937 | 0.927 | 0.957 | 0.971 | 0.842 | 0.746 | 0.663 | 0.522 | 0.423 | 0.485 | 0.495 | 0.397 | 0.362 | 0.356 | 0.852 | 0.803 |
|  | Mis | NA | 0.116 | 0.113 | 0.106 | 0.103 | 0.084 | 0.046 | 0.049 | 0.065 | 0.083 | 0.136 | 0.183 | 0.170 | 0.174 | 0.243 | 0.264 | 0.296 | 0.068 | 0.090 |

^a^Freq: frequency. ^b^Sen: sensitivity. ^c^PPV: positive predictive value. ^d^Mis: misclassification.

Table A46. Ordinal model: Simulation results for true cluster model (E) with an alternative hypothesis (5) using elliptical windows.

|  |  | Maximum reported cluster size (MRCS) | | | | | | | | | | | | | | | | | | Default  Setting |
| --- | --- | --- | --- | --- | --- | --- | --- | --- | --- | --- | --- | --- | --- | --- | --- | --- | --- | --- | --- | --- |
|  |  | 1% | 2% | 3% | 4% | 5% | 6% | 8% | 10% | 12% | 15% | 20% | 25% | 30% | 35% | 40% | 45% | 50% | Overall |  |
| SCIC_1_ | Freq^a^ | 0 | 1 | 1 | 10 | 30 | 44 | 540 | 236 | 65 | 31 | 12 | 10 | 1 | 1 | 0 | 4 | 6 | 992 | 992 |
|  | Sen^b^ | NA | 0.100 | 0.200 | 0.220 | 0.347 | 0.386 | 0.787 | 0.847 | 0.874 | 0.855 | 0.733 | 0.550 | 0.800 | 0.800 | NA | 0.750 | 0.817 | 0.768 | 0.808 |
|  | PPV^c^ | NA | 1.000 | 1.000 | 1.000 | 0.986 | 0.902 | 0.975 | 0.850 | 0.760 | 0.656 | 0.564 | 0.594 | 0.889 | 0.400 | NA | 0.595 | 0.455 | 0.905 | 0.832 |
|  | Mis^d^ | NA | 0.130 | 0.116 | 0.113 | 0.096 | 0.095 | 0.034 | 0.043 | 0.058 | 0.084 | 0.127 | 0.146 | 0.043 | 0.203 | NA | 0.196 | 0.266 | 0.049 | 0.065 |
| SCIC_2_ | Freq | 0 | 1 | 0 | 4 | 24 | 33 | 547 | 254 | 75 | 34 | 14 | 3 | 0 | 0 | 0 | 2 | 1 | 992 | 992 |
|  | Sen | NA | 0.100 | NA | 0.250 | 0.392 | 0.448 | 0.805 | 0.862 | 0.881 | 0.865 | 0.821 | 0.700 | NA | NA | NA | 0.800 | 0.800 | 0.802 | 0.808 |
|  | PPV | NA | 1.000 | NA | 1.000 | 0.934 | 0.894 | 0.974 | 0.851 | 0.765 | 0.661 | 0.548 | 0.454 | NA | NA | NA | 0.530 | 0.800 | 0.904 | 0.832 |
|  | Mis | NA | 0.130 | NA | 0.109 | 0.094 | 0.088 | 0.032 | 0.041 | 0.056 | 0.082 | 0.121 | 0.179 | NA | NA | NA | 0.210 | 0.058 | 0.044 | 0.065 |
| Gini | Freq | 0 | 0 | 1 | 6 | 18 | 20 | 362 | 290 | 134 | 83 | 31 | 9 | 4 | 3 | 5 | 11 | 15 | 992 | 992 |
|  | Sen | NA | NA | 0.300 | 0.333 | 0.406 | 0.495 | 0.798 | 0.855 | 0.870 | 0.870 | 0.845 | 0.878 | 0.825 | 0.867 | 0.900 | 0.900 | 0.940 | 0.820 | 0.808 |
|  | PPV | NA | NA | 1.000 | 1.000 | 0.909 | 0.900 | 0.975 | 0.855 | 0.768 | 0.662 | 0.558 | 0.479 | 0.448 | 0.481 | 0.352 | 0.352 | 0.302 | 0.842 | 0.832 |
|  | Mis | NA | NA | 0.101 | 0.097 | 0.094 | 0.082 | 0.032 | 0.041 | 0.057 | 0.082 | 0.117 | 0.159 | 0.178 | 0.184 | 0.255 | 0.271 | 0.343 | 0.058 | 0.065 |
| Elbow | Freq | 0 | 1 | 2 | 23 | 53 | 46 | 583 | 210 | 52 | 15 | 5 | 1 | 0 | 0 | 0 | 1 | 0 | 992 | 992 |
|  | Sen | NA | 0.100 | 0.300 | 0.487 | 0.555 | 0.530 | 0.822 | 0.862 | 0.846 | 0.727 | 0.860 | 0.500 | NA | NA | NA | 0.900 | NA | 0.793 | 0.808 |
|  | PPV | NA | 1.000 | 0.875 | 0.968 | 0.914 | 0.888 | 0.973 | 0.845 | 0.738 | 0.595 | 0.553 | 0.294 | NA | NA | NA | 0.281 | NA | 0.917 | 0.832 |
|  | Mis | NA | 0.130 | 0.109 | 0.078 | 0.073 | 0.078 | 0.029 | 0.042 | 0.065 | 0.107 | 0.119 | 0.246 | NA | NA | NA | 0.348 | NA | 0.042 | 0.065 |
| MCS  -P | Freq | 0 | 0 | 2 | 12 | 25 | 28 | 455 | 277 | 100 | 53 | 23 | 4 | 3 | 0 | 3 | 4 | 3 | 992 | 992 |
|  | Sen | NA | NA | 0.300 | 0.350 | 0.448 | 0.507 | 0.811 | 0.866 | 0.876 | 0.874 | 0.813 | 0.800 | 0.767 | NA | 0.900 | 0.825 | 0.933 | 0.812 | 0.808 |
|  | PPV | NA | NA | 0.875 | 1.000 | 0.918 | 0.908 | 0.977 | 0.853 | 0.762 | 0.654 | 0.544 | 0.411 | 0.433 | NA | 0.442 | 0.504 | 0.470 | 0.881 | 0.832 |
|  | Mis | NA | NA | 0.109 | 0.094 | 0.087 | 0.080 | 0.030 | 0.041 | 0.057 | 0.084 | 0.123 | 0.192 | 0.184 | NA | 0.222 | 0.192 | 0.242 | 0.048 | 0.065 |
| MCHS  -P | Freq | 0 | 0 | 5 | 18 | 39 | 50 | 409 | 244 | 108 | 65 | 31 | 7 | 3 | 3 | 3 | 4 | 3 | 992 | 992 |
|  | Sen | NA | NA | 0.260 | 0.428 | 0.490 | 0.548 | 0.807 | 0.862 | 0.884 | 0.872 | 0.858 | 0.886 | 0.767 | 0.900 | 0.967 | 0.875 | 0.933 | 0.802 | 0.808 |
|  | PPV | NA | NA | 0.950 | 0.983 | 0.932 | 0.922 | 0.974 | 0.857 | 0.765 | 0.655 | 0.560 | 0.464 | 0.389 | 0.697 | 0.416 | 0.563 | 0.470 | 0.874 | 0.832 |
|  | Mis | NA | NA | 0.110 | 0.085 | 0.080 | 0.073 | 0.031 | 0.040 | 0.056 | 0.084 | 0.116 | 0.170 | 0.208 | 0.106 | 0.227 | 0.174 | 0.242 | 0.051 | 0.065 |

^a^Freq: frequency. ^b^Sen: sensitivity. ^c^PPV: positive predictive value. ^d^Mis: misclassification.

Table A47. Ordinal model: Simulation results for true cluster model (E) with an alternative hypothesis (6) using elliptical windows.

|  |  | Maximum reported cluster size (MRCS) | | | | | | | | | | | | | | | | | | Default  Setting |
| --- | --- | --- | --- | --- | --- | --- | --- | --- | --- | --- | --- | --- | --- | --- | --- | --- | --- | --- | --- | --- |
|  |  | 1% | 2% | 3% | 4% | 5% | 6% | 8% | 10% | 12% | 15% | 20% | 25% | 30% | 35% | 40% | 45% | 50% | Overall |  |
| SCIC_1_ | Freq^a^ | 1 | 1 | 4 | 12 | 38 | 42 | 542 | 222 | 63 | 38 | 10 | 4 | 2 | 2 | 3 | 6 | 5 | 995 | 995 |
|  | Sen^b^ | 0.100 | 0.100 | 0.200 | 0.225 | 0.321 | 0.419 | 0.771 | 0.839 | 0.822 | 0.816 | 0.870 | 0.525 | 0.600 | 0.500 | 0.933 | 0.733 | 0.880 | 0.749 | 0.790 |
|  | PPV^c^ | 1.000 | 1.000 | 1.000 | 1.000 | 0.949 | 0.930 | 0.975 | 0.851 | 0.752 | 0.653 | 0.571 | 0.613 | 0.700 | 0.729 | 0.350 | 0.536 | 0.560 | 0.905 | 0.830 |
|  | Mis^d^ | 0.130 | 0.130 | 0.116 | 0.112 | 0.101 | 0.089 | 0.036 | 0.044 | 0.064 | 0.088 | 0.113 | 0.127 | 0.145 | 0.101 | 0.261 | 0.227 | 0.223 | 0.052 | 0.068 |
| SCIC_2_ | Freq | 0 | 0 | 0 | 9 | 29 | 29 | 547 | 231 | 81 | 44 | 16 | 5 | 1 | 0 | 1 | 1 | 1 | 995 | 995 |
|  | Sen | NA | NA | NA | 0.233 | 0.376 | 0.507 | 0.788 | 0.845 | 0.831 | 0.855 | 0.888 | 0.820 | 0.800 | NA | 1.000 | 0.900 | 0.900 | 0.785 | 0.790 |
|  | PPV | NA | NA | NA | 0.972 | 0.920 | 0.928 | 0.974 | 0.854 | 0.747 | 0.657 | 0.567 | 0.426 | 0.400 | NA | 0.357 | 0.321 | 0.900 | 0.900 | 0.830 |
|  | Mis | NA | NA | NA | 0.113 | 0.096 | 0.078 | 0.034 | 0.043 | 0.065 | 0.085 | 0.115 | 0.183 | 0.203 | NA | 0.261 | 0.290 | 0.029 | 0.047 | 0.068 |
| Gini | Freq | 0 | 0 | 0 | 4 | 21 | 19 | 375 | 255 | 143 | 88 | 32 | 12 | 10 | 3 | 8 | 14 | 11 | 995 | 995 |
|  | Sen | NA | NA | NA | 0.475 | 0.414 | 0.421 | 0.767 | 0.842 | 0.841 | 0.868 | 0.906 | 0.875 | 0.860 | 0.867 | 0.888 | 0.921 | 0.964 | 0.803 | 0.790 |
|  | PPV | NA | NA | NA | 1.000 | 0.913 | 0.939 | 0.972 | 0.855 | 0.755 | 0.666 | 0.559 | 0.463 | 0.517 | 0.356 | 0.381 | 0.431 | 0.350 | 0.837 | 0.830 |
|  | Mis | NA | NA | NA | 0.076 | 0.092 | 0.089 | 0.037 | 0.043 | 0.062 | 0.082 | 0.117 | 0.167 | 0.158 | 0.246 | 0.241 | 0.233 | 0.314 | 0.062 | 0.068 |
| Elbow | Freq | 0 | 0 | 1 | 16 | 47 | 43 | 598 | 201 | 63 | 17 | 7 | 1 | 0 | 0 | 1 | 0 | 0 | 995 | 995 |
|  | Sen | NA | NA | 0.500 | 0.369 | 0.481 | 0.542 | 0.805 | 0.847 | 0.798 | 0.782 | 0.814 | 0.600 | NA | NA | 1.000 | NA | NA | 0.779 | 0.790 |
|  | PPV | NA | NA | 1.000 | 0.984 | 0.924 | 0.915 | 0.973 | 0.845 | 0.734 | 0.613 | 0.486 | 0.316 | NA | NA | 0.357 | NA | NA | 0.917 | 0.830 |
|  | Mis | NA | NA | 0.072 | 0.092 | 0.082 | 0.075 | 0.031 | 0.044 | 0.071 | 0.101 | 0.151 | 0.246 | NA | NA | 0.261 | NA | NA | 0.044 | 0.068 |
| MCS  -P | Freq | 0 | 0 | 1 | 10 | 35 | 27 | 466 | 239 | 117 | 57 | 25 | 5 | 3 | 1 | 3 | 1 | 5 | 995 | 995 |
|  | Sen | NA | NA | 0.500 | 0.410 | 0.443 | 0.481 | 0.781 | 0.861 | 0.852 | 0.874 | 0.888 | 0.820 | 0.833 | 0.900 | 0.867 | 0.900 | 0.880 | 0.794 | 0.790 |
|  | PPV | NA | NA | 1.000 | 0.975 | 0.908 | 0.919 | 0.974 | 0.853 | 0.753 | 0.672 | 0.557 | 0.426 | 0.435 | 0.750 | 0.297 | 0.321 | 0.629 | 0.879 | 0.830 |
|  | Mis | NA | NA | 0.072 | 0.087 | 0.088 | 0.083 | 0.035 | 0.041 | 0.062 | 0.079 | 0.118 | 0.183 | 0.188 | 0.058 | 0.314 | 0.290 | 0.171 | 0.051 | 0.068 |
| MCHS  -P | Freq | 0 | 0 | 2 | 19 | 52 | 50 | 423 | 211 | 113 | 69 | 29 | 8 | 8 | 1 | 2 | 3 | 5 | 995 | 995 |
|  | Sen | NA | NA | 0.300 | 0.395 | 0.448 | 0.532 | 0.774 | 0.854 | 0.854 | 0.884 | 0.900 | 0.875 | 0.850 | 0.800 | 0.800 | 0.933 | 0.940 | 0.776 | 0.790 |
|  | PPV | NA | NA | 1.000 | 0.954 | 0.937 | 0.939 | 0.971 | 0.851 | 0.761 | 0.662 | 0.549 | 0.594 | 0.495 | 0.800 | 0.290 | 0.359 | 0.406 | 0.871 | 0.830 |
|  | Mis | NA | NA | 0.101 | 0.091 | 0.085 | 0.074 | 0.036 | 0.042 | 0.060 | 0.082 | 0.121 | 0.136 | 0.185 | 0.058 | 0.312 | 0.261 | 0.296 | 0.056 | 0.068 |

^a^Freq: frequency. ^b^Sen: sensitivity. ^c^PPV: positive predictive value. ^d^Mis: misclassification.

Table A48. Ordinal model: Simulation results for true cluster model (E) with an alternative hypothesis (7) using elliptical windows.

|  |  | Maximum reported cluster size (MRCS) | | | | | | | | | | | | | | | | | | Default  Setting |
| --- | --- | --- | --- | --- | --- | --- | --- | --- | --- | --- | --- | --- | --- | --- | --- | --- | --- | --- | --- | --- |
|  |  | 1% | 2% | 3% | 4% | 5% | 6% | 8% | 10% | 12% | 15% | 20% | 25% | 30% | 35% | 40% | 45% | 50% | Overall |  |
| SCIC_1_ | Freq^a^ | 0 | 2 | 5 | 7 | 33 | 51 | 566 | 207 | 71 | 29 | 12 | 3 | 3 | 0 | 2 | 3 | 3 | 997 | 997 |
|  | Sen^b^ | NA | 0.100 | 0.180 | 0.214 | 0.397 | 0.425 | 0.783 | 0.857 | 0.835 | 0.841 | 0.783 | 0.700 | 0.633 | NA | 0.700 | 0.500 | 0.900 | 0.763 | 0.812 |
|  | PPV^c^ | NA | 1.000 | 1.000 | 1.000 | 0.981 | 0.952 | 0.976 | 0.854 | 0.753 | 0.660 | 0.550 | 0.469 | 0.557 | NA | 0.656 | 0.833 | 0.634 | 0.915 | 0.832 |
|  | Mis^d^ | NA | 0.130 | 0.119 | 0.114 | 0.089 | 0.087 | 0.034 | 0.042 | 0.063 | 0.085 | 0.123 | 0.164 | 0.184 | NA | 0.203 | 0.097 | 0.150 | 0.048 | 0.063 |
| SCIC_2_ | Freq | 0 | 0 | 2 | 0 | 26 | 36 | 569 | 217 | 78 | 41 | 18 | 4 | 3 | 0 | 1 | 0 | 2 | 997 | 997 |
|  | Sen | NA | NA | 0.300 | NA | 0.427 | 0.486 | 0.804 | 0.865 | 0.853 | 0.849 | 0.850 | 0.825 | 0.767 | NA | 1.000 | NA | 0.850 | 0.802 | 0.812 |
|  | PPV | NA | NA | 1.000 | NA | 0.937 | 0.959 | 0.976 | 0.850 | 0.757 | 0.662 | 0.561 | 0.482 | 0.367 | NA | 0.313 | NA | 0.805 | 0.904 | 0.832 |
|  | Mis | NA | NA | 0.101 | NA | 0.088 | 0.078 | 0.031 | 0.041 | 0.060 | 0.084 | 0.118 | 0.163 | 0.222 | NA | 0.319 | NA | 0.051 | 0.044 | 0.063 |
| Gini | Freq | 0 | 0 | 0 | 7 | 13 | 18 | 362 | 269 | 156 | 88 | 38 | 11 | 10 | 6 | 2 | 7 | 10 | 997 | 997 |
|  | Sen | NA | NA | NA | 0.429 | 0.431 | 0.489 | 0.795 | 0.862 | 0.854 | 0.877 | 0.861 | 0.827 | 0.820 | 0.883 | 0.900 | 0.929 | 0.980 | 0.824 | 0.812 |
|  | PPV | NA | NA | NA | 0.952 | 0.895 | 0.946 | 0.975 | 0.861 | 0.758 | 0.660 | 0.564 | 0.449 | 0.429 | 0.382 | 0.353 | 0.437 | 0.355 | 0.839 | 0.832 |
|  | Mis | NA | NA | NA | 0.085 | 0.091 | 0.079 | 0.033 | 0.040 | 0.060 | 0.083 | 0.116 | 0.174 | 0.194 | 0.234 | 0.254 | 0.228 | 0.313 | 0.057 | 0.063 |
| Elbow | Freq | 0 | 0 | 1 | 24 | 50 | 48 | 615 | 174 | 53 | 21 | 7 | 2 | 2 | 0 | 0 | 0 | 0 | 997 | 997 |
|  | Sen | NA | NA | 0.400 | 0.450 | 0.576 | 0.542 | 0.824 | 0.847 | 0.830 | 0.800 | 0.857 | 0.900 | 0.700 | NA | NA | NA | NA | 0.793 | 0.812 |
|  | PPV | NA | NA | 1.000 | 0.975 | 0.914 | 0.947 | 0.973 | 0.842 | 0.747 | 0.621 | 0.516 | 0.460 | 0.346 | NA | NA | NA | NA | 0.921 | 0.832 |
|  | Mis | NA | NA | 0.087 | 0.082 | 0.070 | 0.072 | 0.029 | 0.045 | 0.064 | 0.097 | 0.135 | 0.174 | 0.232 | NA | NA | NA | NA | 0.042 | 0.063 |
| MCS  -P | Freq | 0 | 0 | 0 | 17 | 39 | 29 | 454 | 225 | 115 | 66 | 28 | 7 | 7 | 2 | 2 | 2 | 4 | 997 | 997 |
|  | Sen | NA | NA | NA | 0.447 | 0.492 | 0.534 | 0.806 | 0.856 | 0.851 | 0.879 | 0.850 | 0.914 | 0.857 | 0.700 | 0.850 | 0.900 | 0.925 | 0.804 | 0.812 |
|  | PPV | NA | NA | NA | 0.952 | 0.932 | 0.946 | 0.975 | 0.855 | 0.753 | 0.657 | 0.557 | 0.454 | 0.473 | 0.280 | 0.523 | 0.527 | 0.564 | 0.874 | 0.832 |
|  | Mis | NA | NA | NA | 0.084 | 0.080 | 0.072 | 0.031 | 0.042 | 0.061 | 0.084 | 0.119 | 0.172 | 0.174 | 0.297 | 0.181 | 0.181 | 0.178 | 0.051 | 0.063 |
| MCHS  -P | Freq | 0 | 0 | 3 | 19 | 30 | 30 | 427 | 223 | 130 | 70 | 35 | 8 | 9 | 5 | 1 | 3 | 4 | 997 | 997 |
|  | Sen | NA | NA | 0.300 | 0.447 | 0.493 | 0.533 | 0.811 | 0.873 | 0.852 | 0.874 | 0.869 | 0.925 | 0.822 | 0.860 | 0.800 | 0.933 | 0.925 | 0.812 | 0.812 |
|  | PPV | NA | NA | 1.000 | 0.969 | 0.910 | 0.958 | 0.972 | 0.864 | 0.751 | 0.654 | 0.563 | 0.490 | 0.494 | 0.374 | 0.296 | 0.590 | 0.564 | 0.866 | 0.832 |
|  | Mis | NA | NA | 0.101 | 0.082 | 0.080 | 0.071 | 0.031 | 0.038 | 0.062 | 0.085 | 0.116 | 0.156 | 0.172 | 0.241 | 0.304 | 0.140 | 0.178 | 0.052 | 0.063 |

^a^Freq: frequency. ^b^Sen: sensitivity. ^c^PPV: positive predictive value. ^d^Mis: misclassification.
